# CRISPR screening identifies DTX4 governing alveolar macrophage cholesterol efflux in pulmonary alveolar proteinosis

**DOI:** 10.1172/jci.insight.201129

**Published:** 2026-07-22

**Authors:** Zimu Wang, Jingwei Shi, Xu Ye, Xinye Xia, Huihui Zhu, Qi Li, Min Chen, Yichao Zhao, Yingwei Zhang, Mengshu Cao, Yonglong Xiao, Xinmei Huang

**Affiliations:** 1 Department of Respiratory and Critical Care Medicine, Nanjing Drum Tower Hospital Clinical College of Traditional Chinese and Western Medicine, Nanjing University of Chinese Medicine, Nanjing, China.; 2 Department of Thoracic Surgery, Nanjing Drum Tower Hospital Clinical College of Traditional Chinese and Western Medicine, Nanjing University of Chinese Medicine, Nanjing, China.; 3 Department of Respiratory and Critical Care Medicine, Nanjing First Hospital, Nanjing University of Chinese Medicine, Nanjing, China.; 4 School of Integrated Chinese and Western Medicine, Nanjing University of Chinese Medicine, Nanjing, China.

**Keywords:** Metabolism, Pulmonology, Cholesterol, Macrophages, Respiration

## Abstract

Pulmonary alveolar proteinosis (PAP) is a rare pulmonary syndrome characterized by impaired surfactant clearance, driven by dysfunctional cholesterol efflux in alveolar macrophages (AMs). However, the molecular determinants governing AM cholesterol homeostasis remain incompletely defined. Here, through a genome-wide CRISPR screen in foamy macrophages and bulk RNA sequencing of AMs from PAP patients, we identify DTX4 as a pivotal regulator of cholesterol efflux in AMs. In mice, AAV-mediated silencing of *DTX4* led to excessive AM lipid accumulation, exacerbated proteinosis, increased lung opacities, and deteriorated pulmonary function. Similarly, DTX4 depletion in primary AMs impaired cholesterol efflux and promoted intracellular lipid deposition. Conversely, AM-specific overexpression of DTX4 in the *Csf2ra^–/–^* PAP model markedly alleviated lipid accumulation, mitigated alveolar proteinosis, restored lung densities, and rescued pulmonary function. Mechanistically, DTX4 stabilizes the GM-CSF receptor via an E3-independent interaction to sustain JAK2/STAT5 signaling, which reciprocally maintains *DTX4* transcription. This positive-feedback loop drives PPARγ expression, and its disruption in PAP impairs cholesterol efflux, a defect partially reversible by ectopic PPARγ expression. Collectively, our findings identify DTX4 as a central orchestrator of AM cholesterol efflux and surfactant homeostasis, positioning it as a promising therapeutic target for PAP.

## Abstract 2

We discovered a protein, DTX4, that helps lung immune cells clear cholesterol, offering a potential new target for treating the rare lung disease PAP.

## Graphical abstract

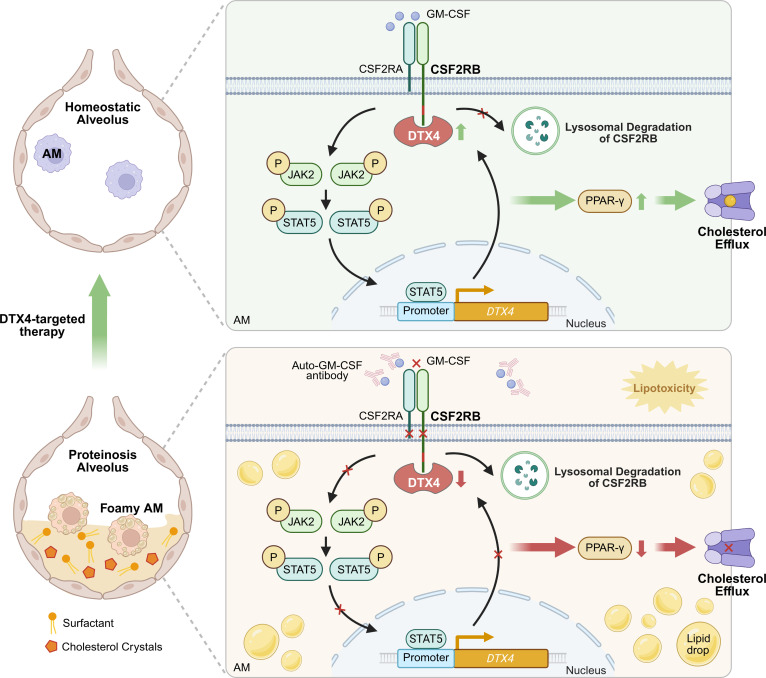

## Introduction

Pulmonary alveolar proteinosis (PAP) is a rare respiratory syndrome characterized by abnormal surfactant accumulation in the alveoli due to impaired clearance by dysfunctional alveolar macrophages (AMs), resulting in compromised gas exchange and respiratory function (1, 2). PAP is classified into 4 main categories: primary (90% of cases), secondary (4% of cases), congenital (1% of cases), and unclassifiable (2). Primary PAP is further stratified into autoimmune and hereditary forms, with the former driven by elevated levels of anti–GM-CSF autoantibodies and the latter arising from pathogenic mutations in the GM-CSF receptor gene *CSF2RA* or *CSF2RB* (3).

While the pathogenesis of PAP is mechanistically heterogeneous, impaired cholesterol homeostasis within AMs has been recognized as a key feature contributing to disease development, particularly in autoimmune and hereditary forms (4, 5). In PAP associated with impaired GM-CSF signaling, AMs exhibit a diminished expression of peroxisome proliferator–activated receptor-γ (PPARγ) and the cholesterol transporter ATP-binding cassette subfamily G member 1 (ABCG1), leading to a profound reduction in cholesterol efflux (6–8). Consequently, esterified cholesterol accumulates within intracytoplasmic lipid droplets, driving the formation of foamy AMs (2, 5). This lipid overload disrupts the uptake and clearance of pulmonary surfactant, exacerbating alveolar dysfunction and contributing to progressive respiratory insufficiency (2, 6, 9–12).

Currently, whole-lung lavage remains the standard therapy for primary PAP; however, its invasive nature and lack of disease-modifying potential limit its long-term efficacy (13). Emerging pharmacological strategies, such as PPARγ agonists and statins, have demonstrated potential in enhancing cholesterol clearance in AMs, underscoring lipid homeostasis as a promising therapeutic target for PAP (7, 11, 14). However, the molecular regulators and mechanisms governing lipid homeostasis remain incompletely understood, highlighting the need for a comprehensive and unbiased analysis of key factors required for cholesterol efflux in AMs.

To address this gap, we integrated bulk RNA sequencing (RNA-seq) analysis of AMs from the bronchoalveolar lavage fluid (BALF) of PAP patients and control donors, with a genome-wide CRISPR activation screen for cholesterol efflux in foamy macrophages derived from THP-1 cells. This approach identified DTX4 as a key regulator of cholesterol efflux in foamy macrophages. Furthermore, we uncovered a previously unrecognized mechanism by which DTX4 governs lipid homeostasis in AMs following cholesterol loading and demonstrated its essential role in DTX4-mediated cholesterol efflux both in vivo in mice and in vitro in primary AMs. Our study also establishes a broadly applicable platform for genome-wide screening of complex functional phenotypes in foamy macrophages, providing an unbiased and scalable framework for the discovery of novel regulatory pathways in macrophage lipid metabolism.

## Results

### The disruption of AM cholesterol efflux and surfactant clearance in PAP.

Alveolar macrophages (AMs) are central to the disruption of surfactant homeostasis in PAP; impaired lipid clearance, particularly of cholesterol, within alveoli is linked to defects in their scavenging function. This dysfunction is primarily attributed to impaired GM-CSF signaling or other mechanisms that hinder cholesterol efflux from AMs. PAP comprises heterogeneous subtypes with distinct etiologies: primary PAP results from disrupted GM-CSF signaling, due to either autoantibodies (autoimmune PAP) or mutations in GM-CSF receptor genes (hereditary PAP); congenital PAP arises from mutations in genes regulating surfactant metabolism or lipid transport (e.g., *ABCA1*, *ABCG1*); and secondary PAP is associated with conditions such as hematologic malignancies, infections, or inhalational exposures that impair AM number or function (Supplemental Figure 1A; supplemental material available online with this article; https://doi.org/10.1172/jci.insight.201129DS1). To confirm previously reported lipid metabolic alterations in PAP, we analyzed AMs from 3 PAP patients and 3 age-matched control donors (Supplemental Table 1). PAP patients exhibited markedly increased BALF turbidity, a robust indicator of surfactant accumulation and disease severity (15) (Supplemental Figure 1B), along with elevated cholesterol-to-phospholipid ratios in BALF (Supplemental Figure 1C). Consistently, levels of free cholesterol, cholesteryl esters, and total cholesterol were significantly increased in PAP-AMs (Supplemental Figure 1D). Importantly, cholesterol efflux from PAP-AMs was impaired in the presence of the cholesterol acceptor apolipoprotein A-I (apoA-I), indicating a defect in reverse cholesterol transport (Supplemental Figure 1E). Morphologically, PAP-AMs displayed a characteristic foamy appearance, with enlarged cell size, cytoplasmic lipid droplet accumulation, and strong lipid staining as evidenced by Oil Red O or BODIPY 493/503 positivity (Supplemental Figure 1, F and G). Furthermore, flow cytometric analysis of *Csf2ra^–/–^* PAP mouse lungs revealed that within the macrophage population (CD45^+^Ly6G^–^CD64^+^MerTK^+^), approximately 77% of the BODIPY^hi^ cells were tissue-resident AMs (TR-AMs; Siglec-F^hi^CD11b^lo^) rather than monocyte-derived macrophages (mo-AMs; Siglec-F^lo^CD11b^hi^) (16) (Supplemental Figure 1, H and I). Consistent with earlier studies (11), our data confirm that impaired cholesterol efflux drives the lipid accumulation and foamy transformation in AMs, establishing them as the central effector cells in the dysregulated lipid metabolism of PAP pathogenesis.

### Bulk RNA-seq identifies key regulators of PAP.

**Figure 1:**
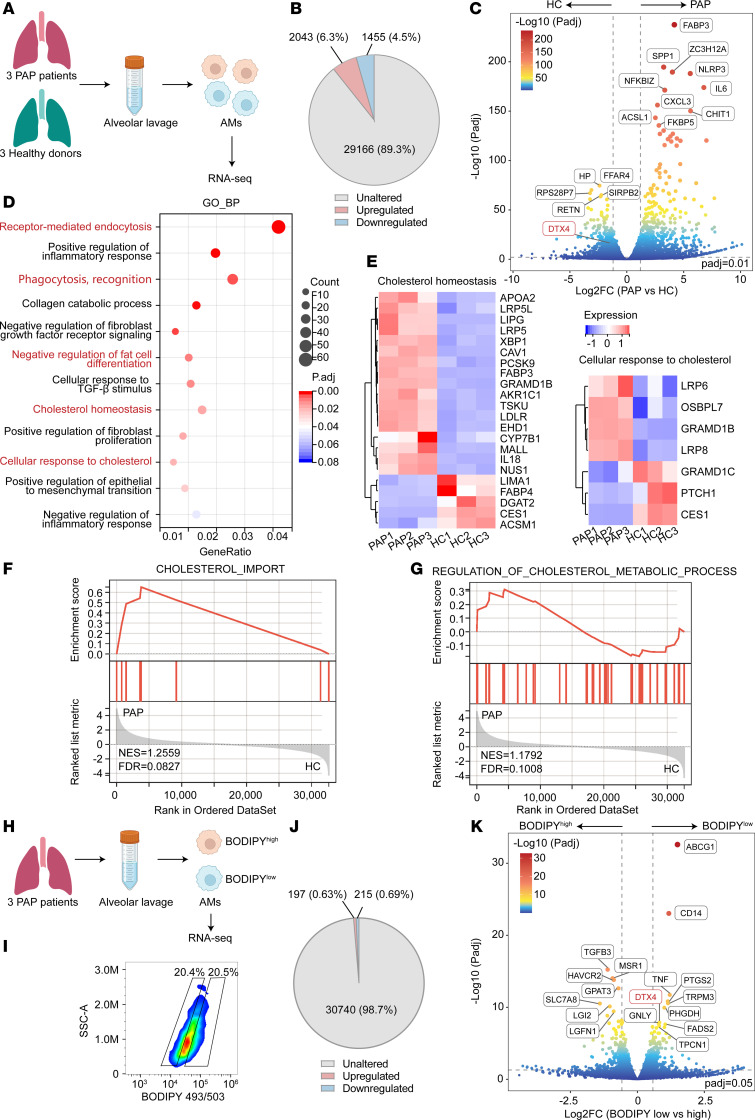
Transcriptomic analysis of AMs in PAP. (**A**) Schematic overview of AM isolation and RNA-seq workflow from autoimmune PAP (aPAP) patients and control donors (*n* = 3). (**B**) Pie chart showing the distribution of differentiallyexpressed genes (DEGs) identified by RNA-seq. (**C**) Volcano plot of DEGs between PAP and control AMs; adjusted *P* values (Padj) were used to determine significance. (**D**) Gene Ontology (GO) enrichment analysis of DEGs, presented as a bubble plot. (**E**) Heatmap showing expression levels of genes involved in cholesterol homeostasis and cholesterol response in PAP versus control AMs. (**F** and **G**) Gene set enrichment analysis (GSEA) demonstrating altered expression of gene sets related to cholesterol transport (**F**) and metabolism (**G**) in PAP. (**H** and **I**) Schematic of AM sorting based on BODIPY fluorescence (BODIPY^hi^ and BODIPY^lo^) from aPAP patients followed by RNA-seq (*n* = 3). (**J**) Pie chart of DEGs identified between BODIPY^hi^ and BODIPY^lo^ AMs. (**K**) Volcano plot of DEGs between BODIPY^hi^ and BODIPY^lo^ AMs; significance was determined using adjusted *P* values (Padj).

To identify key genes involved in the pathogenesis of PAP, we performed transcriptomic profiling of AMs (CD45^+^CD169^+^HLA-DR^+^) isolated from the BALF of PAP patients and age-matched control donors (Figure 1A and Supplemental Figure 2, A and B). All samples were analyzed in biological duplicates, with high intra-group correlation observed across replicates (Supplemental Figure 2C). Differential expression analysis identified 2,043 upregulated and 1,455 downregulated genes in PAP-AMs (Figure 1B). Hierarchical clustering and volcano plot visualization revealed a distinct transcriptional signature, with a subset of genes, including *DTX4*, markedly downregulated in PAP-AMs (Figure 1C and Supplemental Figure 2D). Gene Ontology analysis demonstrated significant enrichment of lipid homeostasis–related terms among differentially expressed genes, particularly those associated with cholesterol metabolism, transport, and response (Figure 1, D and E). Consistent with these findings, Kyoto Encyclopedia of Genes and Genomes (KEGG) pathway analysis and gene set enrichment analysis (GSEA) further corroborated the suppression of cholesterol transport and metabolism pathways in PAP-AMs (Figure 1, F and G, and Supplemental Figure 2, E–G).

To minimize inter-individual variability and better capture gene expression changes directly associated with lipid accumulation, we sorted viable single CD45^+^CD169^+^HLA-DR^+^ PAP-AMs into foamy (BODIPY^hi^) and non-foamy (BODIPY^lo^) populations for RNA-seq analysis (Figure 1, H and I, and Supplemental Figure 2, A and B). Comparative transcriptomic analysis of BODIPY^lo^ non-foamy AMs revealed 197 upregulated and 215 downregulated genes, involving multiple lipid metabolism–related pathways (Figure 1J and Supplemental Figure 3, A–D). Notably, *DTX4*, previously identified as downregulated in bulk PAP-AMs, was among the upregulated genes in BODIPY^lo^ AMs, along with *ABCG1*, a well-established mediator of cholesterol efflux (Figure 1K). Although expression of specific markers such as *FABP4* and *CD14* varied between groups (e.g., elevated in healthy controls or BODIPY^lo^ populations), other canonical markers used to distinguish monocytes from macrophages or AMs from interstitial macrophages showed no significant or consistent differences (Supplemental Figure 3, E and F). These findings suggest that the observed transcriptomic alterations primarily reflect the lipid metabolic status of the cells rather than distinct cell lineage or subpopulations. Collectively, our data point to a dysregulated gene network governing cholesterol homeostasis and foam cell formation in PAP-AMs. However, they are unable to define which genes drive cholesterol efflux under conditions of excessive lipid accumulation.

### Genome-wide CRISPR screen identifies critical determinants for AM cholesterol efflux.

**Figure 2:**
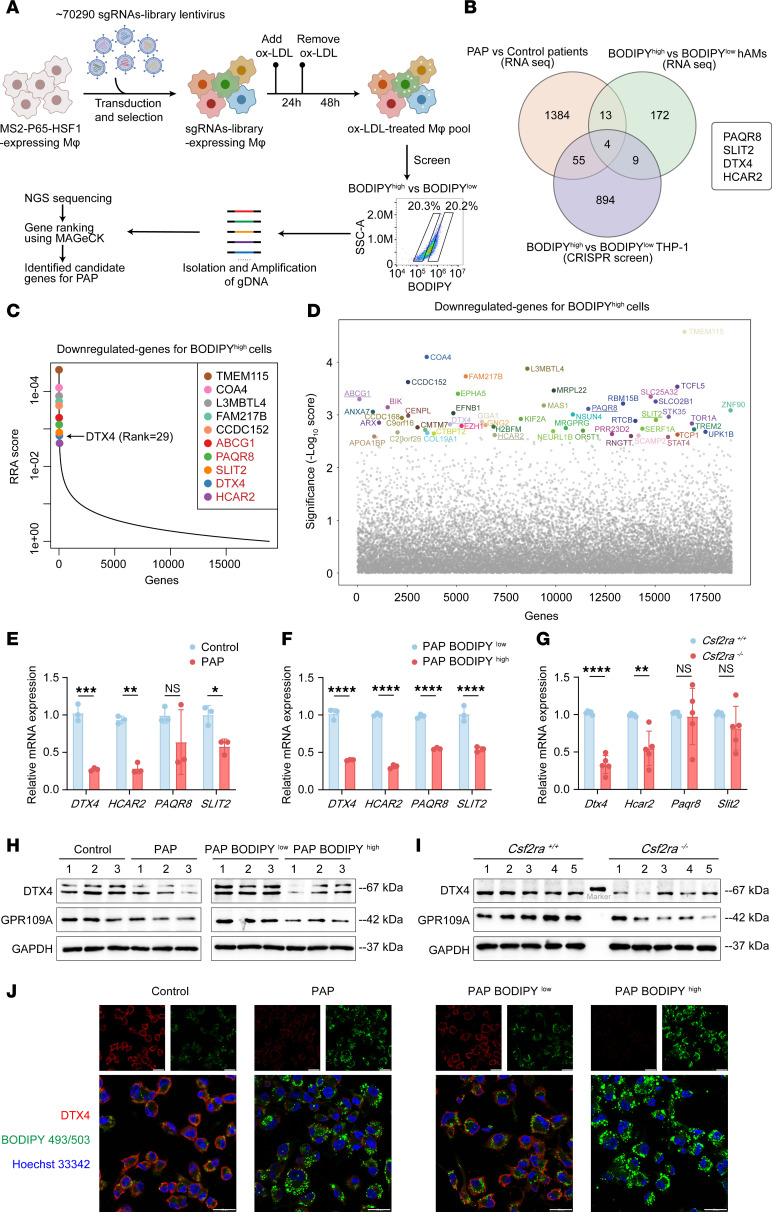
CRISPR screen identifies regulators of cholesterol efflux in AMs. (**A**) Schematic of the CRISPR activation screen workflow. (**B**) Venn diagram showing the overlap of downregulated genes from 3 sequencing datasets. (**C**) Hockey stick plot showing top downregulated genes in BODIPY^hi^ AMs identified by MAGeCK analysis. (**D**) Scatterplot showing the significance of robust rank aggregation (RRA) scores for each gene in BODIPY^lo^ versus BODIPY^hi^ AMs. (**E**–**G**) mRNA expression levels of indicated genes across different comparison groups (**E** and **F**, *n* = 3; **G**, *n* = 5). (**H** and **I**) Protein expression levels of indicated targets across comparison groups (**H**, *n* = 3; **I**, *n* = 5). (**J**) Confocal images showing DTX4 (red) and neutral lipids (BODIPY 493/503, green) in different groups. Nuclei were stained with Hoechst 33342. Scale bars: 30 μm. Statistical comparisons were performed using a 2-tailed unpaired *t* test; *P* values are indicated (*P < 0.05, **P < 0.01, ***P < 0.001, ****P < 0.0001).

To functionally validate and extend RNA-seq findings by identifying causal regulators of cholesterol efflux in foamy AMs, we implemented a genome-wide CRISPR activation screen using the SAM pooled library targeting promoter regions of 23,430 human genes (17). THP-1 cells transduced with MS2-P65-HSF1 activators and the library were loaded with oxidized LDL for 24 hours to induce foam cell formation (18), followed by a 48-hour cholesterol efflux chase upon oxidized LDL removal. Cells were then sorted into BODIPY^hi^ and BODIPY^lo^ populations and subjected to next-generation sequencing to identify cholesterol efflux regulators (Figure 2A and Supplemental Figure 4A). We hypothesized that activation of cholesterol efflux genes would increase the proportion of BODIPY^lo^ cells and enrich corresponding gRNAs. After validating the screen quality and confirming similar gRNA distributions between BODIPY^hi^ and BODIPY^lo^ populations (Supplemental Figure 4B), we applied the MAGeCK algorithm to identify the top 5% differentially enriched genes (19) (Supplemental Figure 4C). By intersecting genes downregulated in BODIPY^hi^ cells with RNA-seq data, we identified 4 candidates potentially involved in cholesterol efflux and PAP pathogenesis: *PAQR8*, *SLIT2*, *DTX4*, and *HCAR2* (Figure 2B). These genes ranked among the most significantly downregulated (Figure 2, C and D, and Supplemental Figure 4, D–F), with *DTX4* showing the largest log_2_ fold change (Supplemental Figure 4, D and G). Notably, the known cholesterol transporter *ABCG1* was downregulated in BODIPY^hi^ cells, supporting the validity of the screen (Figure 2, C and D). We also identified 3 genes, *GJA1*, *PUS10*, and *MSH5*, that were upregulated both in BODIPY^hi^ cells and in PAP samples, indicating potential involvement in disease-related lipid dysregulation (Supplemental Figure 4H).

To validate the CRISPR screen, we examined AMs from PAP patients and control donors, BODIPY^hi^ and BODIPY^lo^ PAP-AMs, and AMs from *Csf2ra^–/–^* PAP mice and *Csf2ra^+/+^* controls (16). Among the candidate genes, only *DTX4* and *HCAR2* showed consistent and significant mRNA differences across all three comparisons (Figure 2, E–G). Protein-level analyses confirmed these trends, with DTX4 exhibiting more pronounced downregulation (Figure 2, H and I, and Supplemental Figure 5, A–C). Importantly, BODIPY staining of intracellular lipid droplets revealed that DTX4 expression was markedly reduced in PAP-AMs, with further suppression observed in the BODIPY^hi^ subset, which also displayed increased lipid accumulation (Figure 2J). Together, these results support a potential role for *DTX4* and *HCAR2* in regulating cholesterol efflux in AMs and implicate its downregulation in PAP pathogenesis.

### DTX4 is essential for AM cholesterol efflux in vitro.

**Figure 3:**
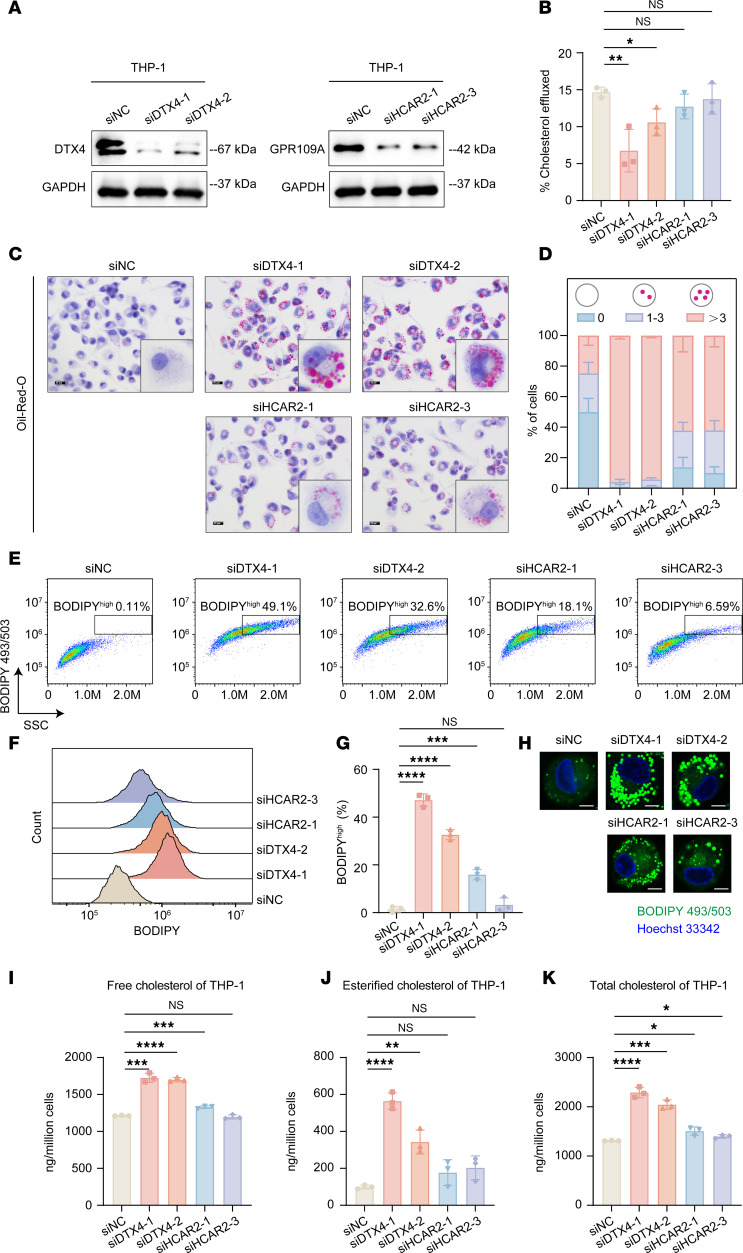
DTX4 knockdown impairs cholesterol efflux in THP-1–derived macrophages in vitro. (**A**) Western blot analysis of indicated proteins in differently treated THP-1–derived macrophages. GAPDH was used as a loading control. (**B**) Quantification of cholesterol efflux using the BODIPY-cholesterol assay (*n* = 3 biological replicates). (**C** and **D**) Representative images (**C**) and quantification (**D**) of Oil Red O staining showing intracellular lipid droplet accumulation under different treatment conditions (*n* = 3 biological replicates). Scale bars: 20 μm. (**E**–**G**) Flow cytometry analysis showing BODIPY fluorescence in THP-1–derived macrophages across treatment groups, including density plots (**E**), ridgeline plot (**F**), and quantification of BODIPY^hi^ cell proportions (**G**) (*n* = 3 biological replicates). (**H**) Confocal images of THP-1–derived macrophages stained with BODIPY 493/503 (green) to visualize neutral lipids (*n* = 3 biological replicates); nuclei were stained with Hoechst 33342 (blue). Scale bars: 5 μm. (**I**–**K**) Quantification of intracellular free cholesterol (**I**), cholesteryl esters (**J**), and total cholesterol (**K**) in THP-1–derived macrophages under different treatments (*n* = 3 biological replicates). Statistical analysis was performed using 1-way ANOVA followed by Tukey’s post hoc test for multiple comparisons (*P < 0.05, **P < 0.01, ***P < 0.001, ****P < 0.0001).

To further delineate the functional roles of the candidate genes in regulating cholesterol efflux and lipid homeostasis in AMs, we performed siRNA-mediated knockdown of *DTX4* and *HCAR2* (encoding GRP109A) in both THP-1–derived macrophages (Figure 3A and Supplemental Figure 5, D and E) and primary murine AMs (Supplemental Figure 6, A and B). Cells were subsequently labeled with BODIPY-cholesterol, and cholesterol efflux to apoA-I was quantified (20). Knockdown of DTX4 resulted in a significant reduction in cholesterol efflux compared with negative control (NC), whereas HCAR2 silencing had no appreciable effect (Figure 3B and Supplemental Figure 6C). Given that impaired cholesterol efflux is a key driver of foam cell formation, we next assessed intracellular lipid accumulation. Oil Red O staining revealed a marked increase in lipid droplet content following DTX4 knockdown in THP-1 cells, while HCAR2 silencing led to only a modest increase (Figure 3, C and D). Similar trends were observed in primary mouse AMs (Supplemental Figure 6, D and E). Consistent with these findings, flow cytometric analysis following BODIPY 493/503 staining demonstrated a significant increase in the proportion of BODIPY^hi^ foamy cells in both THP-1 and murine AMs upon DTX4 depletion, indicative of elevated intracellular neutral lipid accumulation (Figure 3, E–H, and Supplemental Figure 6, F–H). In contrast, HCAR2 knockdown did not produce a comparable effect (Figure 3, E–H, and Supplemental Figure 6, F–H). Moreover, biochemical assays revealed that DTX4 silencing significantly elevated intracellular levels of free cholesterol, cholesteryl esters, and total cholesterol in both cell types (Figure 3, I–K, and Supplemental Figure 6, I–K). Collectively, these data identify DTX4 as a critical regulator of cholesterol efflux and lipid homeostasis in AMs, and implicate its deficiency in promoting foam cell formation, a hallmark of PAP pathogenesis.

### DTX4 deficiency in AM triggers PAP development in vivo.

**Figure 4:**
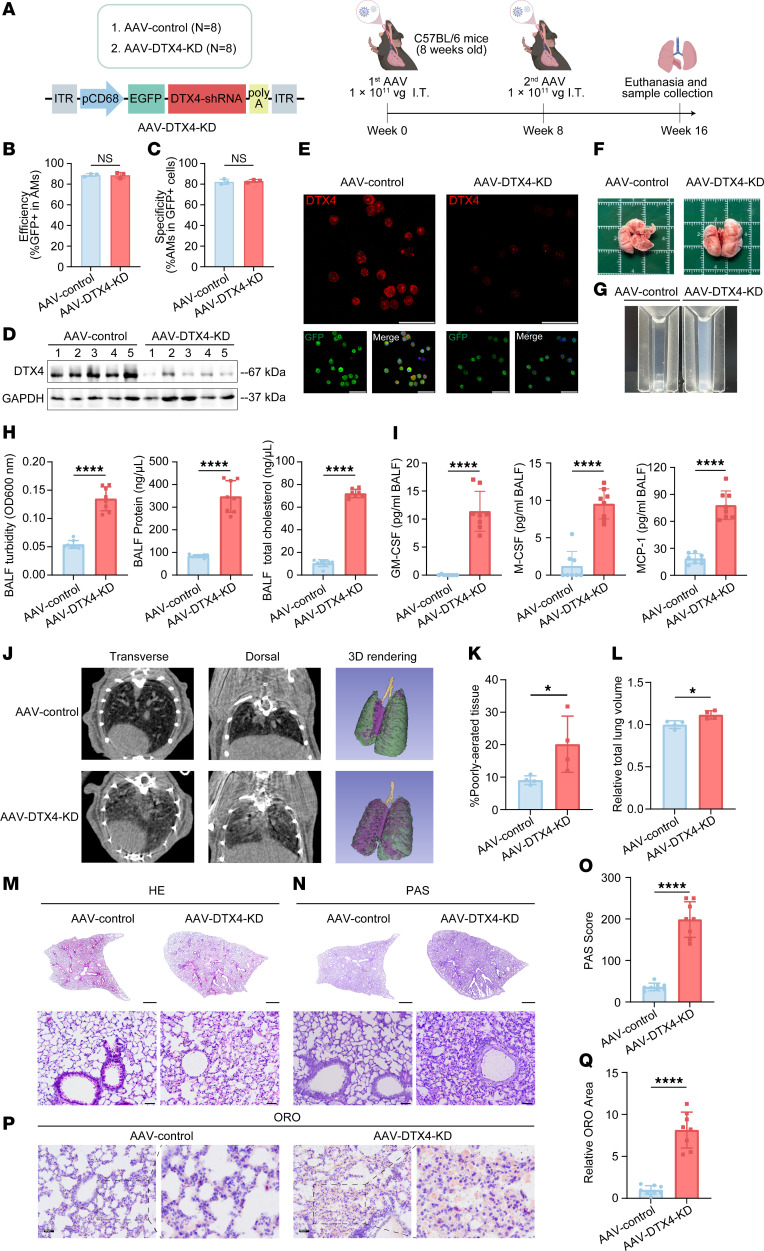
AM-specific DTX4 knockdown impairs pulmonary cholesterol clearance and lung function. (**A**) Schematic showing the design of the AAV vector (left) and the strategy for AM-specific knockdown of DTX4 in vivo (right). (**B** and **C**) Quantification of AAV transduction efficiency (**B**) and targeting specificity (**C**) in AMs by flow cytometry (*n* = 3). (**D**) Western blot analysis of indicated protein in lung tissue; GAPDH was used as loading control (*n* = 5). (**E**) Representative images of DTX4 (red) and GFP (green) expression in the lung; nuclei were stained with Hoechst (blue). Scale bars: 50 μm. (**F**) Gross lung morphology of mice from different groups. (**G**) Representative images of BALF collected from each group. (**H**) Quantification of BALF turbidity, total protein concentration, and total cholesterol levels across groups (*n* = 8). (**I**) Quantification of GM-CSF, M-CSF, and MCP-1 levels in BALF by ELISA (*n* = 8). (**J**) Representative micro-CT images of the lungs from each group, including transverse, dorsal, and 3D reconstructed views. (**K** and **L**) Quantification of poorly aerated lung tissue (**K**) and relative total lung volume (**L**) based on micro-CT analysis (*n* = 4). (**M**) H&E staining of lung sections showing tissue structure (*n* = 8). Scale bars: 1 mm (top), 50 μm (bottom). (**N** and **O**) PAS staining of lung sections with representative images (**N**) and quantification (**O**) (*n* = 8). Scale bars: 1 mm (top), 50 μm (bottom). (**P** and **Q**) Oil Red O staining of lung sections with representative images (**P**) and quantification (**Q**) (*n* = 8). Scale bars: 100 μm. Statistical comparisons were performed using a 2-tailed unpaired *t* test; *P* values are indicated (*P < 0.05, ****P < 0.0001).

To investigate whether DTX4 deficiency under physiological conditions is sufficient to induce PAP in mice, we intratracheally administered an adeno-associated virus (AAV) encoding a DTX4-targeting shRNA or a non-targeting control (1 × 10^11^ viral genomes [vg] per dose, 2 doses 8 weeks apart) into 8-week-old C57BL/6 mice, followed by imaging and histopathological analyses 16 weeks after the initial injection (Figure 4A). An AAV vector driven by the pro-CD68 promoter and carrying an EGFP reporter enabled AM-specific delivery (21). Flow cytometry showed efficient transduction of AMs (88% GFP^+^ in AMs) and high targeting specificity (83% of GFP^+^ cells were AMs), as determined by CD45^+^CD11c^+^Siglec-F^+^ gating (Figure 4, B and C, and Supplemental Figure 7, A and B). DTX4 knockdown was confirmed by Western blot and immunofluorescence in primary AMs isolated from the lung tissues of model mice (Figure 4, D and E, and Supplemental Figure 7C). Throughout the observation period, DTX4-deficient mice showed a decrease in body weight with no effect on survival (Supplemental Figure 7D), a phenotype also observed in multiple established PAP mouse models (22, 23). Notably, DTX4-deficient mice exhibited gross pulmonary enlargement with a milky-white appearance (Figure 4F), along with increased turbidity, protein concentration, and total cholesterol levels in BALF (Figure 4, G and H). Interestingly, the expression of the hPAP BALF biomarkers GM-CSF, M-CSF, and MCP-1 was also elevated in the DTX4-knockdown group (Figure 4I), suggesting that DTX4 deficiency may be linked to dysregulation of GM-CSF signaling and a compensatory cytokine response. No statistically significant reduction in the proportion of AMs was observed in the knockdown group during the observation period (Supplemental Figure 7E).

Micro-CT imaging revealed bilateral, symmetrical ground-glass and patchy high-density opacities, consistent with radiologic features of PAP (Figure 4J). Three-dimensional reconstruction further demonstrated increased total lung volume and a higher proportion of poorly aerated tissue in the DTX4-knockdown group (Figure 4, K and L). Moreover, histological analysis showed preserved alveolar architecture with extensive accumulation of eosinophilic, acellular material within the alveolar spaces, in the absence of marked interstitial inflammation or fibrosis (Figure 4M). Importantly, the intra-alveolar deposits were strongly positive for both periodic acid–Schiff (PAS) and Oil Red O (ORO) staining, indicating the presence of glycoprotein and neutral lipid components, respectively (Figure 4, N–Q). In line with this, ORO^+^ lipid-laden AMs were observed (Figure 4P). Collectively, these findings demonstrate that AM-specific DTX4 deficiency is sufficient to drive PAP-like pathology in mice under homeostatic conditions.

### DTX4 overexpression in AM mitigates PAP progression.

**Figure 5:**
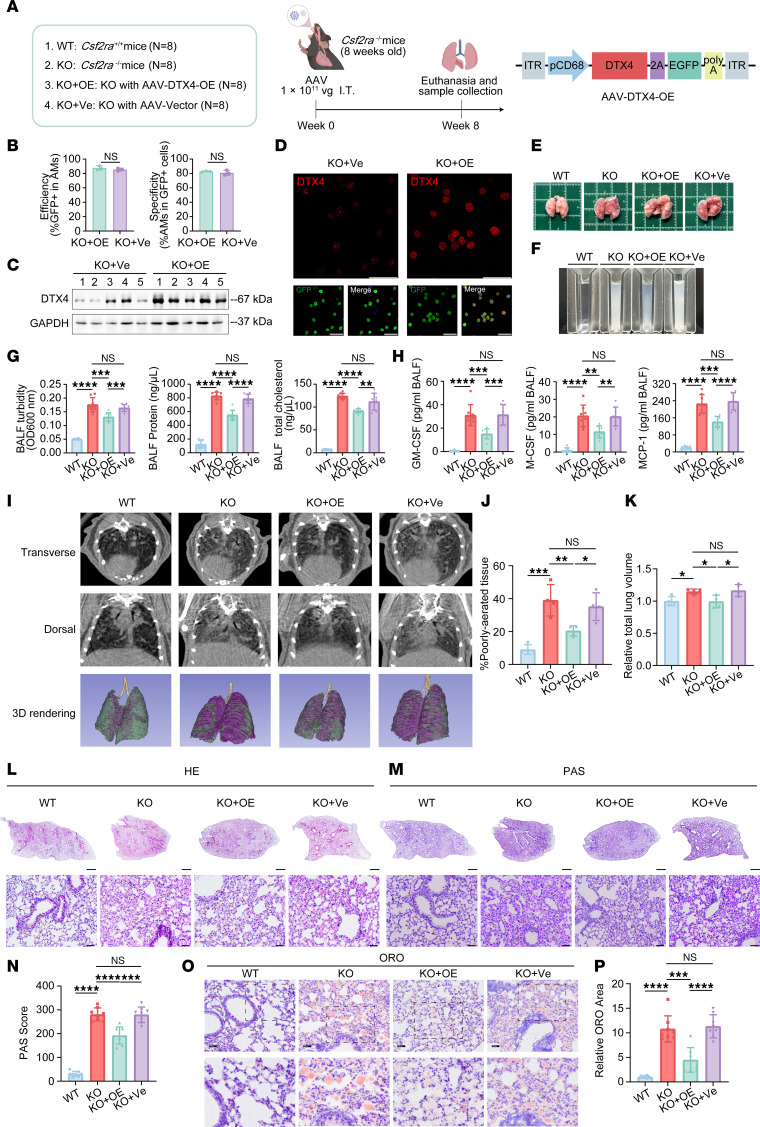
AM-specific DTX4 overexpression ameliorates PAP phenotype in *Csf2ra^–/–^* mice. (**A**) Schematic showing the strategy for AM-specific overexpression of DTX4 in vivo (left) and the design of the AAV vector (right). (**B**) Quantification of AAV transduction efficiency and targeting specificity in AMs by flow cytometry (*n* = 3). (**C**) Western blot analysis of indicated protein in lung tissue (*n* = 5); GAPDH was used as loading control. (**D**) Representative images of DTX4 (red) and GFP (green) expression in the lung; nuclei were stained with Hoechst (blue). Scale bars: 50 μm. (**E**) Gross lung morphology of mice from different groups. (**F**) Representative images of BALF collected. (**G**) Quantification of BALF turbidity, total protein concentration, and total cholesterol levels across groups (*n* = 8). (**H**) Quantification of GM-CSF, M-CSF, and MCP-1 levels in BALF by ELISA (*n* = 8). (**I**) Representative micro-CT images of the lungs from each group, including transverse, dorsal, and 3D reconstructed views. (**J** and **K**) Quantification of poorly aerated lung tissue (**J**) and relative total lung volume (**K**) based on micro-CT analysis (*n* = 4). (**L**) H&E staining of lung sections showing tissue structure (*n* = 8). Scale bars: 1 mm (top), 50 μm (bottom). (**M** and **N**) PAS staining of lung sections with representative images (**M**) and quantification (**N**) (*n* = 8). Scale bars: 1 mm (top), 50 μm (bottom). (**O** and **P**) Oil Red O staining of lung sections with representative images (**O**) and quantification (**P**) (*n* = 8). Scale bars: 100 μm. Statistical comparisons were made using 1-way ANOVA followed by Tukey’s post hoc test for multiple comparisons, or a 2-tailed unpaired Student’s *t* test for comparisons between 2 groups (*P < 0.05, **P < 0.01, ***P < 0.001, ****P < 0.0001).

Next, we wanted to test whether DTX4 can be targeted therapeutically in established PAP lesions. To this end, we used a spontaneous PAP mouse model generated by CRISPR-mediated knockout of *Csf2ra* (*Csf2ra^–/–^*, KO), which develops PAP symptoms by 8 weeks of age (16). At this stage, mice received intratracheal administration of a pro-CD68 promoter–driven AAV encoding DTX4 or an empty vector control, followed by evaluation 8 weeks after treatment through imaging and histopathological analyses (Figure 5A). Flow cytometry showed efficient transduction of AMs in AAV-DTX4–treated mice (88% GFP^+^ in AMs) and high targeting specificity (83% of GFP^+^ cells were AMs) (Figure 5B). DTX4 overexpression (OE) in AMs was confirmed by Western blot and immunofluorescence (Figure 5, C and D, and Supplemental Figure 8A). During the observation period, DTX4 overexpression mitigated weight loss in KO mice without affecting survival (Supplemental Figure 8B). Hallmark pathological features of PAP, including lung enlargement, increased BALF turbidity, elevated protein concentration, and total cholesterol levels, were significantly alleviated in the DTX4-OE group, whereas vector-treated controls showed no improvement (Figure 5, E–G). In addition, DTX4 overexpression reduced the elevated levels of GM-CSF, M-CSF, and MCP-1 observed in BALF of KO mice (Figure 5H). DTX4 overexpression also partially restored the reduction in AM proportions observed in KO mice (Supplemental Figure 8C). Notably, DTX4 knockdown did not impair the migratory capacity of murine AMs or THP-1 cells (Supplemental Figure 8D), nor did it affect cell viability or lineage markers (Supplemental Figure 8, E and F). These findings indicate that the therapeutic benefit of DTX4 restoration is primarily attributable to the correction of lipid metabolic dysregulation, independent of changes in cell survival, migratory capacity, or population shifts. In addition, micro-CT imaging showed resolution of diffuse reticular and patchy opacities in the lungs of DTX4-treated mice (Figure 5, I–K). Histological analysis revealed reduced accumulation of eosinophilic, acellular material within the alveolar spaces (Figure 5L). Although foamy AMs and granulocytes persisted, PAS staining revealed a marked reduction in glycoprotein accumulation (Figure 5, M and N), while ORO staining showed decreased extracellular lipid deposition in the DTX4-OE group (Figure 5, O and P), indicating effective attenuation of surfactant components. Taken together, these findings demonstrate that reconstitution of DTX4 expression can effectively ameliorate PAP pathology, even after disease onset.

### DTX4 stabilizes the GM-CSF receptor via a non-canonical positive-feedback loop.

**Figure 6:**
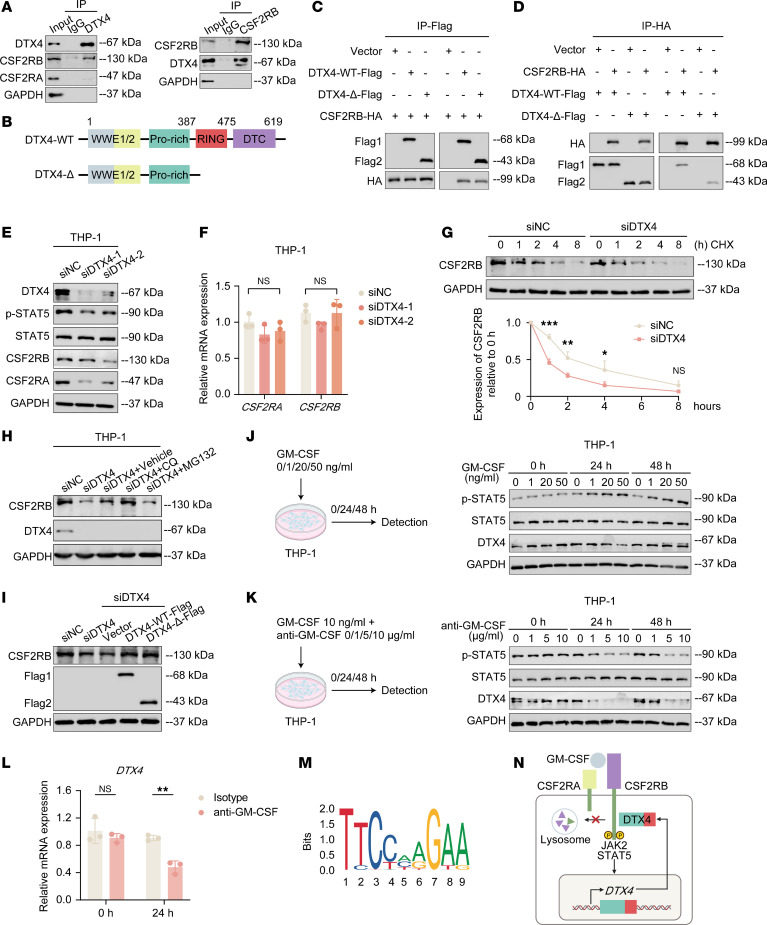
DTX4 stabilizes CSF2RB as an E3-independent scaffold within a positive-feedback loop. (**A**) Endogenous co-immunoprecipitation (co-IP) of DTX4 and CSF2RB in THP-1 cells. IgG served as an isotype control. (**B**) Schematic representation of wild-type DTX4 and the RING domain deletion mutant (DTX4-Δ). (**C** and **D**) Co-IP analysis in HEK293T cells cotransfected with HA-CSF2RB and FLAG–DTX4-WT or FLAG–DTX4-Δ. Interactions were assessed by immunoprecipitation with anti-FLAG (**C**) or anti-HA (**D**) antibodies followed by immunoblotting. (**E** and **F**) Western blot analysis of signaling proteins (**E**) and RT-PCR quantification (**F**) of indicated protein or genes in DTX4-knockdown (DTX4-KD) THP-1 cells (*n* = 3 biological replicates). (**G**) Cycloheximide (CHX) chase assay showing CSF2RB protein turnover in control and DTX4-KD THP-1 cells. Representative blots (top) and quantification of half-life (bottom) are shown (*n* = 3 biological replicates). (**H** and **I**) Western blot analysis of indicated proteins in DTX4-KD THP-1 cells following treatment with chloroquine (CQ; 50 μM) or MG132 (20 μM) (**H**), or upon re-expression of DTX4-WT or DTX4-Δ (**I**). (**J**–**L**) Regulation of DTX4 by GM-CSF signaling. Western blot analysis of DTX4 expression in THP-1 cells following recombinant GM-CSF stimulation (**J**) or anti–GM-CSF neutralizing antibody treatment (**K**). RT-qPCR analysis of DTX4 mRNA upon GM-CSF neutralization (**L**) (*n* = 3 biological replicates). (**M**) Sequence logo of the high-confidence STAT5 binding motif within the *DTX4* promoter predicted by JASPAR. (**N**) Schematic model: DTX4 acts as an E3-independent scaffold to stabilize CSF2RB, sustaining JAK2/STAT5 signaling, which reciprocally drives DTX4 transcription, forming a positive-feedback loop. Statistical comparisons were made using 1-way ANOVA followed by Tukey’s post hoc test for multiple comparisons, or unpaired 2-tailed Student’s *t* test for comparisons between 2 groups (*P < 0.05, **P < 0.01, ***P < 0.001).

Canonically, DTX4 functions rely on its RING domain to mediate receptor trafficking and signaling activation (24). To elucidate its role in GM-CSF signaling, we first performed endogenous co-immunoprecipitation in THP-1 cells, revealing a physical interaction between DTX4 and the GM-CSF receptor complex, particularly the β subunit (CSF2RB) (Figure 6A). To map this interaction, we coexpressed HA-tagged CSF2RB with either FLAG-tagged wild-type DTX4 (DTX4-WT) or a RING domain deletion mutant (DTX4-Δ) in HEK293T cells (Figure 6B). Co-immunoprecipitation assays confirmed that both DTX4-WT and DTX4-Δ robustly bound to full-length CSF2RB (Figure 6, C and D), indicating that the interaction is independent of the E3 ligase activity–bearing RING domain.

Functionally, while DTX4 knockdown did not alter *CSF2RA* or *CSF2RB* mRNA levels, it substantially reduced their protein abundance and blunted downstream STAT5 phosphorylation (Figure 6, E and F, and Supplemental Figure 9, A–C). Cycloheximide chase assays further demonstrated a shortened half-life of CSF2RB protein in DTX4-deficient cells (Figure 6G). Notably, treatment with the lysosomal inhibitor chloroquine, but not the proteasome inhibitor MG132, reversed this reduction, suggesting that DTX4 protects CSF2RB from lysosomal degradation (Figure 6H and Supplemental Figure 9D). Crucially, re-expression of either DTX4-WT or the E3-dead DTX4-Δ in DTX4-knockdown cells was sufficient to rescue CSF2RB protein levels (Figure 6I and Supplemental Figure 9E). These findings establish a non-canonical mechanism whereby DTX4 acts as a scaffold protein to stabilize the GM-CSF receptor complex independent of its E3 ubiquitin ligase activity.

Given the downregulation of DTX4 in AMs from PAP patients and *Csf2ra^–/–^* mice (Figure 2, H and I), we investigated whether a regulatory relationship exists between GM-CSF signaling and DTX4 expression. While exogenous GM-CSF stimulation did not further induce DTX4 levels in THP-1 cells or AMs (Figure 6J and Supplemental Figure 10, A–C), blockade of GM-CSF signaling using a neutralizing antibody significantly suppressed DTX4 expression at both protein and mRNA levels (Figure 6, K and L, and Supplemental Figure 10, D–G). Consistent with transcriptional maintenance, bioinformatics analysis identified high-confidence STAT5 binding sites within the *DTX4* promoter (Figure 6M and Supplemental Figure 10H). Collectively, these data point to a positive-feedback loop: DTX4 acts as a non-canonical scaffold to stabilize CSF2RB, thereby sustaining the JAK2/STAT5 signaling axis, which in turn feeds back to maintain DTX4 transcription and expression (Figure 6N).

### Disruption of the DTX4/GM-CSF axis suppresses PPARγ and impairs cholesterol efflux in AM.

**Figure 7:**
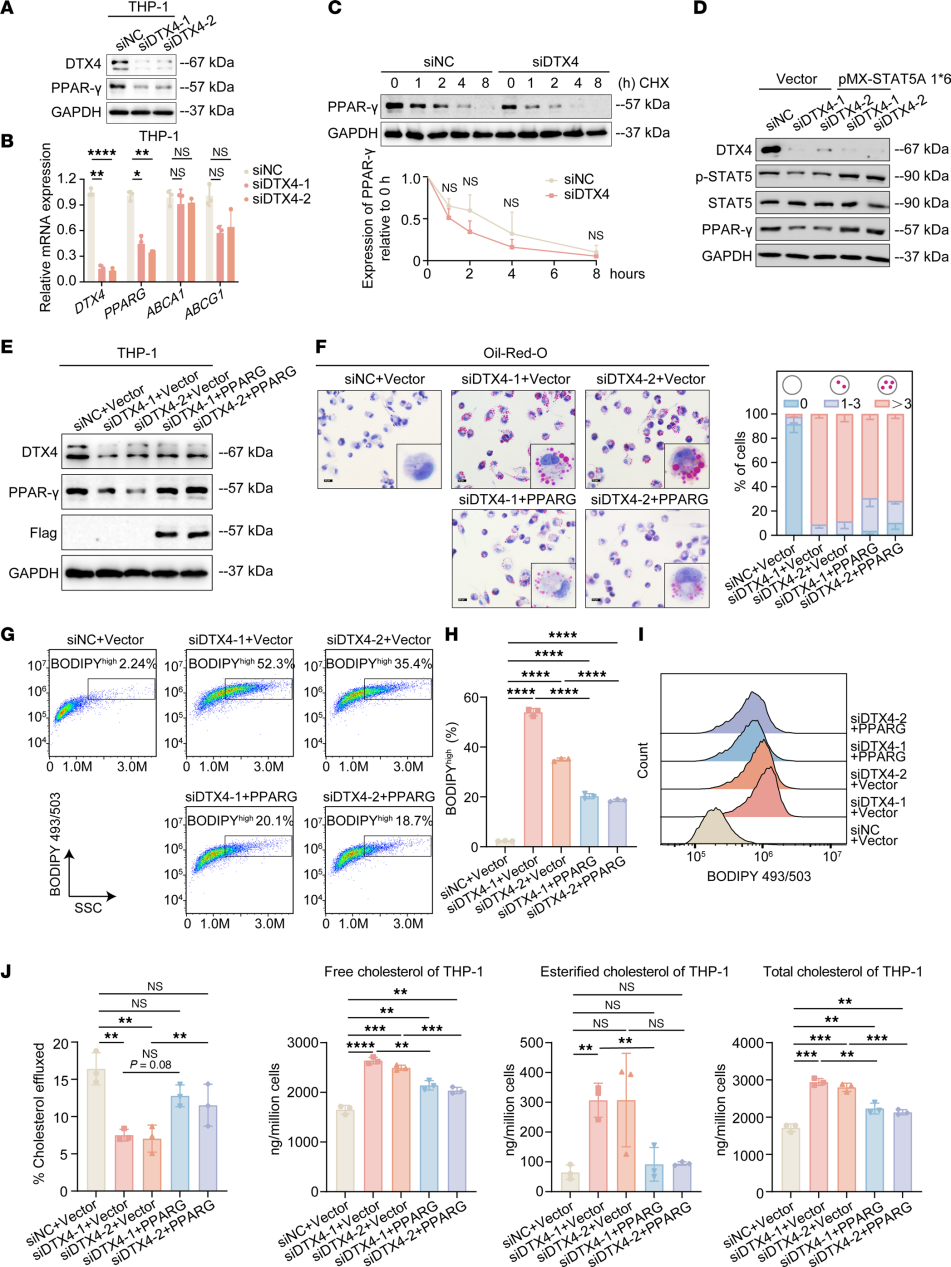
Disruption of the DTX4–GM-CSF loop drives PAP pathogenesis via PPARγ suppression. (**A** and **B**) Western blot (**A**) and RT-qPCR (**B**) analysis of PPARγ expression in DTX4-KD THP-1 cells (*n* = 3 biological replicates). (**C**) Cycloheximide (CHX) chase assay analyzing PPARγ protein stability in control and DTX4-KD THP-1 cells. Representative blots (top) and quantification of half-life (bottom) are shown (*n* = 3 biological replicates). (**D** and **E**) Western blot analysis of indicated proteins in DTX4-KD THP-1 cells rescued with constitutively active STAT5 (pMX-STAT5A1*6) (**D**) or ectopic PPARγ expression (**E**) (*n* = 3 biological replicates). (**F**–**I**) Assessment of lipid accumulation in DTX4-KD THP-1 cells following PPARγ overexpression. Shown are representative Oil Red O staining with quantification (**F**), and flow cytometric analysis of BODIPY 493/503 fluorescence presented as density plots (**G**), bar graphs (**H**), and ridgeline plots (**I**) (*n* = 3 biological replicates). Scale bars: 20 μm. (**J**) Analysis of cholesterol efflux capacity and intracellular cholesterol content in DTX4-KD THP-1 cells rescued with PPARγ overexpression (*n* = 3 biological replicates). Statistical analysis was performed using 1-way ANOVA followed by Tukey’s post hoc test for multiple comparisons (*P < 0.05, **P < 0.01, ***P < 0.001, ****P < 0.0001).

We next interrogated how the DTX4/GM-CSF axis governs cholesterol efflux. Our initial RNA-seq analysis enriched the PPAR pathway, a critical downstream effector of GM-CSF governing lipid regulation, in BODIPY^lo^ versus BODIPY^hi^ populations (Supplemental Figure 3C). Consistent with this, DTX4 silencing precipitated a marked reduction in PPARγ expression at both the mRNA and protein levels (Figure 7, A and B, and Supplemental Figure 11, A–C). This suppression was transcriptional, as DTX4 depletion did not alter PPARγ protein turnover (Figure 7C). Crucially, PPARγ levels were fully restored by re-expression of either wild-type or E3 ligase–dead DTX4 (Supplemental Figure 11, D and E). Furthermore, introduction of a constitutively active STAT5 mutant (STAT5A1*6) was sufficient to rescue PPARγ expression in DTX4-depleted cells (Figure 7D and Supplemental Figure 11F). Thus, DTX4 maintains PPARγ expression transcriptionally via the GM-CSF/STAT5 axis, independent of its E3 ligase activity.

To delineate the functional significance of this axis, we assessed whether restoring PPARγ could rescue the lipid defects caused by DTX4 loss. Indeed, ectopic expression of PPARγ significantly attenuated lipid droplet accumulation (Figure 7, E–I, and Supplemental Figure 11G) and partially restored cholesterol efflux capacity (Figure 7J) in DTX4-deficient THP-1 cells. Intriguingly, pharmacological activation of PPARγ using rosiglitazone was less effective than genetic overexpression in mitigating lipid accumulation (Supplemental Figure 11, H–M) and restoring cholesterol efflux (Supplemental Figure 11, N and O). This likely reflects the profound depletion of the PPARγ protein pool, rendering pharmacological activation inefficient and underscoring the therapeutic value of targeting upstream DTX4. Together, our results identify DTX4 as a central molecular hub whose downregulation in PAP disrupts a GM-CSF–dependent positive-feedback loop, extinguishing downstream STAT5 signaling and PPARγ expression, thereby abrogating cholesterol efflux and driving alveolar proteinosis.

## Discussion

Hypothesis-driven approaches have identified numerous regulators of functional shifts in foamy AMs in PAP, including GM-CSF signaling, PPARγ, and PU.1 (3, 25, 26). Building on the landmark success of GM-CSF therapy for autoimmune PAP, validation of novel therapeutic targets for other PAP subtypes remains a critical priority (27, 28). While genetic screening has been applied to investigate lipid uptake in foamy macrophages in atherosclerosis (18, 29), systematic studies exploring cholesterol efflux in PAP AMs have yet to be conducted. We approached this by integrating bulk RNA-seq data from PAP patients with CRISPR screens targeting cholesterol efflux. While we acknowledge that the rarity of PAP restricted our transcriptomic cohort size, this approach helped mitigate sample size limitations, facilitating the robust identification of conserved lipid regulators. Our analysis identified many known regulators of lipid homeostasis, illuminating the key genes essential for cholesterol metabolism and transport in PAP. Notably, we identified and validated DTX4 as a key regulator of cholesterol efflux in foamy AMs, demonstrating its functional role through both loss- and gain-of-function assays in vitro and in vivo.

DTX4 is a member of the Deltex family of E3 ubiquitin ligases, characterized by a conserved RING domain that mediates substrate ubiquitination and proteasomal degradation (30). Functionally, DTX4 plays a pivotal role in Notch signaling regulation (24) and has been implicated in diverse biological processes, including adipogenesis (31), immune modulation (32), and oncogenesis (30, 33). Emerging evidence highlights its involvement in lipid metabolism, where DTX4 modulates triglyceride and cholesterol homeostasis in bovine mammary epithelial cells via interaction with microRNA-485 (34). Specifically, microRNA-485 suppresses DTX4 expression, leading to increased triglyceride accumulation and reduced cholesterol content, suggesting a regulatory axis in milk fat synthesis (34). In thyroid cancer, DTX4 promotes tumor progression by upregulating stearoyl-CoA desaturase 1 (SCD), a key enzyme in lipid biosynthesis, underscoring its oncogenic potential (33). Moreover, its downregulation has been linked to disrupted lipid storage, leading to excessive accumulation of lysophosphatidic acid and free fatty acids (35). Our study elucidates a role for DTX4 in PAP, where it modulates lipid homeostasis in AMs. We demonstrate that DTX4 deletion impairs cholesterol efflux, leading to intracellular lipid droplet accumulation and pathological protein deposition in lung tissue. Conversely, DTX4 overexpression in PAP model mice alleviates these abnormalities. These findings not only align with recent reports on DTX4’s role in lipid metabolism but also underscore its distinct function in disease-specific lipid dysregulation.

Mechanistically, our study identifies DTX4 as a critical stabilizer of GM-CSF signaling integrity. Emerging evidence from *Csf2ra*- or GM-CSF–knockout mice has shown that impaired GM-CSF signaling leads to widespread downregulation of lipid regulatory networks at both the transcriptional and functional levels (16, 36). Recent research also suggests that GM-CSF signaling stabilizes the GM-CSF receptor complex at the protein level through proteostasis-related pathways (37). These insights align with our findings, where DTX4 acts as a non-canonical, E3-independent scaffold that physically interacts with CSF2RB to avert its lysosomal degradation. This stabilization of the receptor complex is pivotal for sustaining the downstream JAK2/STAT5 signaling axis, which, in a reciprocal manner, drives *DTX4* transcription. This regulatory architecture establishes a self-reinforcing positive-feedback loop that is essential for maintaining AM homeostasis.

In both human and murine PAP AMs, PPARγ expression is diminished as a result of disrupted GM-CSF signaling, prompting clinical trials of PPARγ agonists like rosiglitazone in autoimmune PAP patients (8, 14, 38–40). Consistent with this, prior research has shown that DTX4 knockdown suppresses PPARγ expression, impairing lipid droplet formation and adipogenic differentiation in 3T3-L1 preadipocytes (31). However, contrasting findings exist: in mouse models of alcoholic fatty liver disease, DTX4 expression is reduced, yet DTX4 knockdown promotes PPARγ expression and intracellular lipid droplet accumulation in HepG2 cells (41). Aligning with the specific context of AM biology, our results demonstrate that DTX4 deficiency precipitates PPARγ downregulation and lipid overload via the collapse of the GM-CSF signaling axis. Mechanistically, we clarified the hierarchy of this regulation. Although DTX4 facilitates the degradation of substrates like TBK1 via its canonical E3 ligase activity (32, 42), we found that DTX4 depletion did not alter PPARγ protein turnover, corroborating previous reports that DTX4 is not a direct E3 ligase for PPARγ (41). Importantly, while ectopic PPARγ expression partially rescued cholesterol efflux in DTX4-deficient cells, pharmacological activation using rosiglitazone proved significantly less effective. This disparity likely reflects the profound depletion of the PPARγ protein pool in the absence of DTX4, identifying DTX4 as a superior upstream therapeutic target compared with residual downstream effectors. Nevertheless, the partial rescue by PPARγ implies that DTX4 likely governs AM function via additional pathways. Future multiomics profiling of DTX4-modulated macrophages is warranted to fully map this downstream landscape and define its broader role in lipid homeostasis. Crucially, linking these molecular discoveries to the cellular level through deeper immunophenotyping with a broader array of markers will be essential to understand the impact of DTX4 across distinct macrophage subsets and their functional states.

Our screen has identified several highly ranked genes, including *TMEM115*, *COA4*, *L3MBTL4*, *FAM217B*, *CCDC152*, *ABCG1*, *HCAR2*, *PAQR8*, and *SLIT2*, which may play critical roles in AM function. Among these, ABCG1 is a well-established driver of cholesterol efflux (43, 44), validating the effectiveness of our screen model in identifying key molecules involved in AM cholesterol outflow. TMEM115 and COA4 are implicated in membrane trafficking and mitochondrial function (45, 46), respectively, while L3MBTL4 and FAM217B are associated with epigenetic regulation and cellular homeostasis (47, 48). CCDC152 may contribute to cytoskeletal organization (49), and HCAR2, PAQR8, and SLIT2 are linked to lipid metabolism and signaling pathways (50–52). To further uncover genes with specific relevance to AM function and PAP pathogenesis, we integrated our screen results with RNA-seq data. Intriguingly, *HCAR2*, *PAQR8*, and *SLIT2* consistently emerged across 3 differential gene sets, suggesting their potential as disease-specific mediators of AM cholesterol homeostasis and PAP progression. However, subsequent validation experiments confirmed that DTX4 exhibited the most significant expression changes and functional relevance. Additionally, a group of genes upregulated in PAP, particularly *GJA1*, *PUS10*, and *MSH5*, warrant further exploration for their potential roles in disease mechanisms.

### Conclusion.

In summary, our study identifies DTX4 as a key driver of cholesterol efflux in AMs, addressing a critical gap in PAP pathogenesis. Conditional deletion of DTX4 in AMs precipitated PAP progression, whereas restoring DTX4 in established lesions effectively reduced lipid burden. Mechanistically, DTX4 functions as an E3-independent scaffold to stabilize the GM-CSF receptor, thereby sustaining a positive-feedback loop that drives PPARγ expression. Disruption of this axis in PAP extinguishes downstream signaling and impairs cholesterol efflux. These findings establish DTX4 as a central node in AM lipid homeostasis and a promising therapeutic target for PAP and related disorders of cholesterol dysregulation.

## Methods

### Sex as a biological variable.

In the human and murine experiments, males and females were included.

### Animal model and treatment.

*Csf2ra* gene–deficient (*Csf2ra^–/–^*) mice (strain NO. S-KO-16939) and C57BL6/J wild-type mice were purchased from Cyagen (Suzhou, China). All mice were bred and housed in specific pathogen–free animal facilities maintained by the Nanjing First Hospital Experimental Animal Center. The facilities were kept at 22°C with a 12-hour light/12-hour dark cycle. Experiments were conducted using an equal number of male and female *Csf2ra^–/–^* mice or *Csf2ra^+/+^* littermate controls. The primers for genotyped PCR are listed in Supplemental Table 2.

For AAV-mediated DTX4 overexpression in AMs, *Csf2ra^–/–^* mice at 8 weeks of age were intratracheally given 1 × 10^11^ vg/50 μL of pAAV-pro-CD68-DTX4-EGFP or control vector. For AAV-mediated DTX4 knockdown, male C57BL6/J mice at 8 and 16 weeks of age were intratracheally given 1 × 10^11^ vg/50 μL of pAAV-pro-CD68-EGFP-shDTX4 or control shRNA negative control (shNC) viruses. The AAV vectors were constructed and packaged by GenePharma (Suzhou, China) and Corues Biotechnology (Nanjing, China) at a final titer of about 1 × 10^13^ vg/mL. The pro-CD68 promoter was used to drive AM-specific expression of DTX4 or shRNA constructs, and an EGFP reporter was included to enable cell tracking (21). For intratracheal administration, mice were anesthetized with tribromoethanol and positioned at a 45° incline, and AAV vectors were delivered into the trachea under direct visualization using a fiber-optic light source. Mice were held upright for 2 minutes to facilitate uniform pulmonary distribution.

All mice were euthanized at the experimental endpoint (16 weeks of age for the OE group and 24 weeks of age for the knockdown group) for tissue collection and subsequent analysis. Lungs were inflation-fixed via the trachea with 4% paraformaldehyde (PFA) in PBS at a constant pressure of 25 cm H_2_O, followed by tracheal ligation, excision, and overnight fixation in 4% PFA at 4°C.

### Cell culture.

The human monocytic cell line THP-1 (ATCC TIB-202, RRID: CVCL_0006) was obtained from the American Type Culture Collection (ATCC). The cell line was routinely tested and confirmed to be free of mycoplasma contamination. The cells were cultured in RPMI 1640 (Gibco) supplemented with 10% heat-inactivated fetal bovine serum (FBS; HyClone) and 1% penicillin-streptomycin (PS; HyClone) and maintained in a humidified incubator with 5% CO_2_ at 37°C. Differentiation of THP-1 cells into macrophages was induced by treatment of the cells with 100 ng/mL phorbol 12-myristate 13-acetate (PMA; catalog P1585, Sigma-Aldrich).

### BALF collection and AM culture.

Human bronchoalveolar lavage fluid (BALF) and alveolar macrophages (hAMs) were obtained from patients undergoing flexible bronchoscopy for unexplained chronic cough or from patients with PAP receiving whole-lung lavage. Murine BALF and alveolar macrophages (mAMs) were obtained by bronchoalveolar lavage with 1 mL of cold saline, repeated 5 times. BALF was filtered through a 200 μm cell strainer and centrifuged at 250*g* for 10 minutes, after which the cellular pellet was resuspended in culture medium and allowed to adhere to tissue culture plastic for 2 hours to isolate AMs. hAMs were maintained in Dulbecco’s modified Eagle medium (DMEM; Gibco) supplemented with 10% FBS, 1% PS, 1 mM sodium pyruvate, 10 mM HEPES, and 20 ng/mL recombinant human GM-CSF (Amoytop). mAMs were cultured in DMEM supplemented with 10% FBS, 1% PS, 30 ng/mL recombinant mouse GM-CSF (catalog 415-ML, R&D Systems), 10 ng/mL transforming growth factor-β1 (catalog 7666-MB, R&D Systems), and 1 μM rosiglitazone (catalog R2408, Sigma-Aldrich) to enable long-term in vitro maintenance and expansion (53).

### Transfection.

For THP-1 cell transfection, cells were treated with 100 ng/mL PMA, followed by transfection with siRNA or plasmid using Lipofectamine 3000 (catalog L-3000015, Thermo Fisher Scientific) according to the manufacturer’s protocol. For mAM transfection, cells were resuspended in Opti-MEM (catalog 31985070, Gibco) and adjusted to a density of 6 × 10^6^ cells/mL. Three hundred microliters of cell suspension was transferred to a 0.2 cm electroporation cuvette and transfected using the Gene Pulser Xcell Electroporation System (Bio-Rad) at 125 V and 20 mA. The siRNA for DTX4 or HCAR2 knockdown and the plasmid for PPARG overexpression were designed and synthesized by GenePharma. Expression vectors for FLAG-tagged DTX4-WT, FLAG-tagged DTX4-Δ, HA-tagged CSF2RB, and pMX-STAT5A1*6 were obtained from Applied Biological Materials.

### Oil Red O staining.

Cells were stained using the Oil Red O staining kit (catalog C0157, Beyotime) following the manufacturer’s instructions. Cells were fixed with 4% PFA for 10 minutes and washed twice with 1× PBS. Subsequently, cells were immersed in staining wash solution for 20 seconds, and incubated in Oil Red O working solution for 20 minutes. The stained cells were rinsed with staining wash solution for 30 seconds and washed twice with 1× PBS. Then cells were treated with hematoxylin staining solution (catalog C0107, Beyotime) for 1 minute, and washed twice with 1× PBS. Cells were coverslipped using mounting solution (catalog P0126, Beyotime) and subjected to microscopic observation and imaging.

### Immunostaining.

Cells were cultured on glass coverslips, fixed with 4% PFA for 15 minutes at room temperature, and subsequently blocked with PBS containing 10% goat serum for 30 minutes. Primary antibodies against DTX4 were applied at a 1:200 dilution and incubated overnight at 4°C. Fluorescently conjugated secondary antibodies were applied at a 1:100 dilution for 1 hour at room temperature. Lipid droplets were visualized using 10 μM BODIPY 493/503 (1:1,000; catalog HY-W090090, MedChemExpress) for 30 minutes at room temperature. Nuclei were counterstained with Hoechst 33342 (1:100; catalog C0031, Solarbio) for 5 minutes. Imaging was performed on a STELLARIS 5 Confocal Microscope (Leica), and images were processed using LAS X software. The antibodies used for immunostaining are listed in Supplemental Table 3.

### Lipid analysis.

The Amplex Red Cholesterol and Cholesteryl Ester Assay Kit (catalog S0211, Beyotime) and the Phospholipids Assay Kit (catalog M1243L96, Mlbio) were used to quantify cellular or BALF levels of free cholesterol, cholesteryl esters, total cholesterol, and phospholipids, following the manufacturers’ protocols.

### Cell migration assay.

Cell migration was assessed using 24-well Transwell inserts with 8.0-μm-pore-size PET membranes (catalog 353097, Falcon). mAMs or differentiated THP-1 cells (1.5 × 10^5^ cells in 250 μL serum-free medium) were seeded into the upper chamber, while 750 μL medium supplemented with 15% FBS was added to the lower chamber as a chemoattractant. After incubation for 30 hours at 37°C, non-migrating cells were removed, and migrating cells on the lower surface were fixed with 4% PFA, stained with 0.1% crystal violet, and quantified by microscopy.

### BODIPY-cholesterol efflux assay.

The preparation of 10× BODIPY-cholesterol and the subsequent cholesterol efflux assay were performed with minor modifications to a previously described protocol (20). In brief, cells were labeled with BODIPY-cholesterol (catalog HY-125746, MedChemExpress) by incubation in labeling medium (DMEM) containing 1× BODIPY-cholesterol and 2 μg/mL Sandoz (catalog S9318, Sigma-Aldrich), an acetylcholinesterase inhibitor, for 1 hour. After the labeling step, cells were washed and equilibrated in DMEM supplemented with 0.2% bovine serum albumin, 2 μg/mL Sandoz, and 0.3 mM cyclic adenosine 3′,5′-monophosphate (cAMP; catalog C8988, Sigma-Aldrich) for 16 hours. Subsequently, the cells were washed again and incubated in phenol red–free DMEM containing 2 μg/mL Sandoz and 10 μg/mL ApoA1 (catalog 118-30-1047S, Fitzgerald), which served as cholesterol acceptors, for 4 hours. Efflux medium and cell lysates were collected, and fluorescence intensity was quantified using a TECAN Spark microplate reader (excitation wavelength 482 nm, emission wavelength 515 nm). Baseline-corrected cholesterol efflux was calculated as the ratio of fluorescence in the efflux medium to the total fluorescence.

### Bulk RNA-seq collection and analysis.

Total RNA was isolated from AMs using TRIzol reagent (Invitrogen), followed by DNase I (Takara) treatment to remove genomic DNA contamination. RNA integrity and concentration were assessed using an Agilent 2100 Bioanalyzer (Agilent Technologies). Sequencing libraries were prepared from 1.5 μg of total RNA using the NEBNext Ultra RNA Library Prep Kit for Illumina (New England Biolabs) according to the manufacturer’s protocol. Libraries were sequenced on an Illumina NovaSeq 6000 platform (Annoroad Gene Technology) to generate 150 bp paired-end reads. Clean reads were aligned to the reference genome using STAR aligner (https://github.com/alexdobin/STAR). SNP calling was performed using GATK3 (Broad Institute; https://gatk.broadinstitute.org/), while gene expression quantification was conducted using HTSeq (v0.5.4; https://htseq.readthedocs.io/). Transcript abundance was normalized as FPKM (fragments per kilobase of transcript per million mapped reads). Differential expression analysis was performed using DESeq2 with an adjusted *P* value threshold of 0.05. Functional enrichment analysis was conducted through gene set enrichment analysis (GSEA) using the Molecular Signatures Database (MSigDB), with Gene Ontology terms analyzed across biological processes, molecular functions, and cellular components (10,000 permutations, FDR-corrected). The analysis scripts are available at the following GitHub repository: https://github.com/huangxm2017/RNAseq-DEG-Analysis

### CRISPR screen.

We followed previously published protocols of library preparation using the CRISPR activation pooled library SAM (Synergistic Activation Mediator) v2 (catalog 1000000078, Addgene), as developed by the Zhang laboratory, with slight modifications (17, 54). For viral transduction, duplicate samples, each containing 1.2 × 10^8^ MS2-P65-HSF1-expressing THP-1 cells, were infected at an MOI of 0.3. Transduced cells were selected using 8 μg/mL blasticidin for 7 days and then cultured for an additional 7 days before screening. To identify cholesterol efflux genes, THP-1 cells were treated with 100 μg/mL oxidized LDL (catalog 20605ES10, Yeasen) for 24 hours, followed by a 48-hour chase. Cells were sorted by BODIPY 493/503 staining, and genomic DNA from the top and bottom 20% of labeled cells was extracted, amplified, and sequenced on an Illumina NovaSeq. sgRNA spacer sequences were mapped using count_spacers.py, and normalized read counts were analyzed with MAGeCK (19). The analysis scripts are available at https://github.com/huangxm2017/CRISPRa-screen-analysis

### Co-immunoprecipitation.

Cells were lysed in NP-40 lysis buffer (catalog KGP5206, Keygen Biotech) supplemented with protease and phosphatase inhibitor cocktails. Lysates were clarified by centrifugation, and 1 mg of total protein was incubated overnight at 4°C with 2 μg of primary antibody or IgG control with rotation. Immune complexes were captured using 25 μL of rProtein G Magarose Beads (catalog SM004001, Smart-Lifesciences) for 3 hours at 4°C. Beads were washed 6 times with lysis buffer, and bound proteins were eluted by boiling in SDS-PAGE sample buffer for subsequent Western blot analysis.

### Western blot.

For Western blot analysis, proteins were extracted from cells using cell lysis buffer (catalog KGP2100, Keygen Biotech). Aliquots of protein extracts were separated on 12% SDS-PAGE gels before transfer onto a polyvinylidene fluoride membrane. Membranes were blocked in Tris-buffered saline with Tween-20 (TBST) containing 5% nonfat milk for 1 hour and incubated with primary antibodies overnight at 4°C, followed by incubation with the peroxidase-coupled secondary antibodies for 1 hour at room temperature. Signal detection was performed using the Tanon 5200 chemiluminescence imaging system, and resulting images were analyzed using ImageJ (NIH). The antibodies used for Western blot are listed in Supplemental Table 3.

### RT-qPCR.

Total RNA was isolated using TRIzol reagent, and cDNA synthesis was performed with the HiScript II Reverse Transcriptase kit (R323-01, Vazyme). qPCR was conducted using ChamQ Universal SYBR qPCR Master Mix (Q711-02, Vazyme) on a QuantStudio 6 Flex Real-Time PCR System. Gene expression levels were normalized to β-actin and calculated using the 2^–ΔΔCt^ method. Primer sequences are listed in Supplemental Table 2.

### Flow cytometry analysis and cell sorting.

For flow analysis, THP-1 cells, hAMs, and mAMs were harvested and resuspended at a density of 1 × 10^6^ cells per sample. Cells were stained with fluorophore-conjugated antibodies (0.5 μg per 10^6^ cells in 100 μL) and/or BODIPY 493/503 (1:1,000) for 30 minutes on ice. After staining, cells were washed 3 times with 1 mL PBS. Fluorescence signals were acquired using a CytoFLEX flow cytometer (Beckman Coulter). Data were analyzed using FlowJo software (v10.0). For BODIPY 493/503–based cell sorting, cells were processed on a FACSAria II sorter (BD Biosciences). Positivity percentages were determined from biological triplicates. The antibodies used for FACS are listed in Supplemental Table 3.

### Statistics.

The data are presented as mean ± SD. Statistical comparisons were made using 1-way ANOVA followed by Tukey’s post hoc test for multiple comparisons, or a 2-tailed unpaired Student’s *t* test for comparisons between 2 groups. *P* < 0.05 was considered statistically significant. All statistical analyses were performed using GraphPad Prism software (version 9) or R (version 4.2.3).

### Study approval.

This study was approved by the Institutional Review Board (IRB) of Nanjing Drum Tower Hospital, Nanjing University Medical School (approval no. IRB 2025-0459-01). Human samples were obtained from deidentified, residual BALF collected during routine clinical procedures, with a waiver of written informed consent due to the use of anonymized samples and the minimal-risk nature of the research. Patients or the public were not involved in the design, conduct, reporting, or dissemination of this study. All animal experiments were approved by the Institutional Animal Care and Use Committee of Nanjing Drum Tower Hospital. A summary of clinical characteristics is provided in Supplemental Table 1.

### Data availability.

The data supporting the findings of this study are available within the article and its supplemental material. The raw sequencing data were deposited in the NCBI’s Gene Expression Omnibus (GEO) database (accession nos. GSE299689, GSE299693, and GSE299696). The underlying values for all data points shown in graphs are provided in the Supporting Data Values file.

## Author contributions

XH designed and supervised the study with support from YX. ZW, JS, XY, and XH performed the majority of the experiments and data acquisition, with contributions from XX, HZ, QL, M Chen, Y Zhao, Y Zhang, and M Cao. All authors contributed to the methodology. XH and ZW performed data analysis and visualization, with contributions from JS and XY. XH provided resources and funding. ZW, JS, and XY provided important intellectual contributions. XH wrote the original manuscript with contributions from ZW and JS. XH, ZW, JS, and XY reviewed and edited the manuscript, with input from all coauthors. ZW, JS, and XY contributed equally to this work. The authorship order among co–first authors was determined by their chronological involvement with the project.

## Conflict of interest

The authors have declared that no conflict of interest exists.

## Funding support

- National Natural Science Foundation of China (82400089 to XH).
- Natural Science Foundation of Jiangsu Province (BK20230140 to XH).

## Supplementary Material

Supplemental data

Unedited blot and gel images

Supporting data values

## Version 1 07/22/2026

Electronic publication

## References

[1] Salvaterra E, Campo I (2020). Pulmonary alveolar proteinosis: from classification to therapy. Breathe (Sheff).

[2] Trapnell BC (2019). Pulmonary alveolar proteinosis. Nat Rev Dis Primers.

[3] Huang X (2023). Alveolar macrophages in pulmonary alveolar proteinosis: origin, function, and therapeutic strategies. Front Immunol.

[4] Baker AD (2010). PPARgamma regulates the expression of cholesterol metabolism genes in alveolar macrophages. Biochem Biophys Res Commun.

[5] Dorr D (2022). C/EBPβ regulates lipid metabolism and Pparg isoform 2 expression in alveolar macrophages. Sci Immunol.

[6] Thomassen MJ (2007). ABCG1 is deficient in alveolar macrophages of GM-CSF knockout mice and patients with pulmonary alveolar proteinosis. J Lipid Res.

[7] Sallese A (2017). Targeting cholesterol homeostasis in lung diseases. Sci Rep.

[8] Malur A (2011). Restoration of PPARγ reverses lipid accumulation in alveolar macrophages of GM-CSF knockout mice. Am J Physiol Lung Cell Mol Physiol.

[9] Carey B, Trapnell BC (2010). The molecular basis of pulmonary alveolar proteinosis. Clin Immunol.

[10] Huang J (2024). Causal role of lipid metabolism in pulmonary alveolar proteinosis: an observational and mendelian randomisation study. Thorax.

[11] McCarthy C (2018). Statin as a novel pharmacotherapy of pulmonary alveolar proteinosis. Nat Commun.

[12] De Aguiar Vallim TQ (2017). ABCG1 regulates pulmonary surfactant metabolism in mice and men. J Lipid Res.

[13] Campo I (2016). Whole lung lavage therapy for pulmonary alveolar proteinosis: a global survey of current practices and procedures. Orphanet J Rare Dis.

[14] Dupin C (2020). Pioglitazone in pulmonary alveolar proteinosis: promising first clinical experience. Respir Med Res.

[15] Suzuki T (2014). Pulmonary macrophage transplantation therapy. Nature.

[16] Shima K (2022). A murine model of hereditary pulmonary alveolar proteinosis caused by homozygous Csf2ra gene disruption. Am J Physiol Lung Cell Mol Physiol.

[17] Joung J (2017). Genome-scale CRISPR-Cas9 knockout and transcriptional activation screening. Nat Protoc.

[18] Patterson MT (2023). Trem2 promotes foamy macrophage lipid uptake and survival in atherosclerosis. Nat Cardiovasc Res.

[19] Li W (2014). MAGeCK enables robust identification of essential genes from genome-scale CRISPR/Cas9 knockout screens. Genome Biol.

[20] Raulin AC (2023). An assay to evaluate the capacity of cholesterol acceptors using BODIPY-cholesterol in cells. STAR Protoc.

[21] Hou Y (2020). Epigenetic modulation of macrophage polarization prevents lumbar disc degeneration. Aging (Albany NY).

[22] Ferretti A (2016). Autoantibody-mediated pulmonary alveolar proteinosis in Rasgrp1-deficient mice. J Immunol.

[23] Li F (2022). Gene therapy of Csf2ra deficiency in mouse fetal monocyte precursors restores alveolar macrophage development and function. JCI Insight.

[24] Chastagner P (2017). Ligand-activated Notch undergoes DTX4-mediated ubiquitylation and bilateral endocytosis before ADAM10 processing. Sci Signal.

[25] Ouimet M, Marcel YL (2012). Regulation of lipid droplet cholesterol efflux from macrophage foam cells. Arterioscler Thromb Vasc Biol.

[26] Yan J, Horng T (2020). Lipid metabolism in regulation of macrophage functions. Trends Cell Biol.

[27] Antoniu SA (2020). Pharmacotherapy options in pulmonary alveolar proteinosis. Expert Opin Pharmacother.

[28] Kumar A (2018). Pulmonary alveolar proteinosis in adults: pathophysiology and clinical approach. Lancet Respir Med.

[29] Robichaud S (2021). Identification of novel lipid droplet factors that regulate lipophagy and cholesterol efflux in macrophage foam cells. Autophagy.

[30] Scalia P (2023). The DTX protein family: an emerging set of E3 ubiquitin ligases in cancer. Cells.

[31] Wang Z (2017). E3 ubiquitin ligase DTX4 is required for adipogenic differentiation in 3T3-L1 preadipocytes cell line. Biochem Biophys Res Commun.

[32] Cui J (2012). NLRP4 negatively regulates type I interferon signaling by targeting the kinase TBK1 for degradation via the ubiquitin ligase DTX4. Nat Immunol.

[33] Cui Y (2023). Deltex E3 ubiquitin ligase 4 promotes thyroid cancer progression through stearoyl-CoA desaturase 1. Funct Integr Genomics.

[34] Liu J (2021). MiR-485 targets the DTX4 gene to regulate milk fat synthesis in bovine mammary epithelial cells. Sci Rep.

[35] Nahalka J (2021). Theoretical analysis of S, M and N structural proteins by the protein-RNA recognition code leads to genes/proteins that are relevant to the SARS-CoV-2 life cycle and pathogenesis. Front Genet.

[36] Trapnell BC (2009). Pulmonary alveolar proteinosis, a primary immunodeficiency of impaired GM-CSF stimulation of macrophages. Curr Opin Immunol.

[37] Hansen G (2008). The structure of the GM-CSF receptor complex reveals a distinct mode of cytokine receptor activation. Cell.

[38] Woloszczak J (2024). A comprehensive outlook on pulmonary alveolar proteinosis-a review. Int J Mol Sci.

[39] Vis DC (2020). Reduction in alveolar macrophage size in refractory autoimmune pulmonary alveolar proteinosis after treatment with pioglitazone. J Bronchology Interv Pulmonol.

[40] Bonfield TL (2008). Peroxisome proliferator-activated receptor-gamma regulates the expression of alveolar macrophage macrophage colony-stimulating factor. J Immunol.

[41] https://ecommons.luc.edu/luc_diss/3934.

[42] Lu LF (2026). Fish CDK2 recruits Dtx4 to degrade TBK1 through ubiquitination in the antiviral response. Elife.

[43] Xu D (2022). Structure and transport mechanism of the human cholesterol transporter ABCG1. Cell Rep.

[44] Zou J (2022). Asprosin inhibits macrophage lipid accumulation and reduces atherosclerotic burden by up-regulating ABCA1 and ABCG1 expression via the p38/Elk-1 pathway. J Transl Med.

[45] Ong YS (2014). TMEM115 is an integral membrane protein of the Golgi complex involved in retrograde transport. J Cell Sci.

[46] Lambrechts RA (2019). CoA-dependent activation of mitochondrial acyl carrier protein links four neurodegenerative diseases. EMBO Mol Med.

[47] Liu X (2016). Genome wide association study identifies L3MBTL4 as a novel susceptibility gene for hypertension. Sci Rep.

[48] Kang K (2016). A genome-wide methylation approach identifies a new hypermethylated gene panel in ulcerative colitis. Int J Mol Sci.

[49] Harima R (2024). Ccdc152 is not necessary for male fertility, but contributes to maintaining sperm morphology. J Reprod Dev.

[51] Yusuf B (2021). The neurorepellent, Slit2, prevents macrophage lipid loading by inhibiting CD36-dependent binding and internalization of oxidized low-density lipoprotein. Sci Rep.

[52] Chen S (2023). PAQR8 promotes breast cancer recurrence and confers resistance to multiple therapies. Breast Cancer Res.

[53] Gorki AD (2022). Murine ex vivo cultured alveolar macrophages provide a novel tool to study tissue-resident macrophage behavior and function. Am J Respir Cell Mol Biol.

[54] Huang X (2023). A self-propagating c-Met-SOX2 axis drives cancer-derived IgG signaling that promotes lung cancer cell stemness. Cancer Res.

