# Supplementary material for: CRISPR screening identifies DTX4 governing alveolar macrophage cholesterol efflux in pulmonary alveolar proteinosis
Source: JCI Insight. 2026 Jul 22;11(14):e201129. doi: 10.1172/jci.insight.201129 (PMC13461153; doi:10.1172/jci.insight.201129)
Supplement: Supplementary file 1 [file jciinsight-11-201129-s008.pdf]

Supplementary Materials for

CRISPR Screening Identifies DTX4 Governing Alveolar Macrophage Cholesterol Efflux in Pulmonary Alveolar Proteinosis

Authors

Zimu Wang[1#], Jingwei Shi[3#], Xu Ye[4#], Xinye Xia[1], Huihui Zhu[1], Qi li[1], Min Chen[1], Yichao Zhao[1], Yingwei Zhang[1], Mengshu Cao[1], Yonglong Xiao[1], Xinmei Huang[1,2]*

Affiliations

1. Department of Respiratory and Critical Care Medicine, Nanjing Drum Tower Hospital Clinical College of Traditional Chinese and Western Medicine, Nanjing University of Chinese Medicine, Nanjing, 210000, China.

2. School of Integrated Chinese and Western Medicine, Nanjing University of Chinese Medicine, Nanjing, 210023, China.

3. Department of Thoracic Surgery, Nanjing Drum Tower Hospital Clinical College of Traditional Chinese and Western Medicine, Nanjing University of Chinese Medicine, Nanjing, 210000, China.

4. Department of Respiratory and Critical Care Medicine, Nanjing First Hospital, Nanjing University of Chinese Medicine, Nanjing, 210000, China.

***Corresponding author: Xinmei Huang** **Email: huangxm2017@pku.edu.cn**

Nanjing University of Chinese Medicine, No. 138 Xianlin Road, Nanjing, 210023, China

Tel: 86-18801183320, Fax: 86-025-83106666

This PDF file includes:

Supplementary Figures. 1 to 11

Supplementary Tables. 1 to 3

Supplementary figure 1

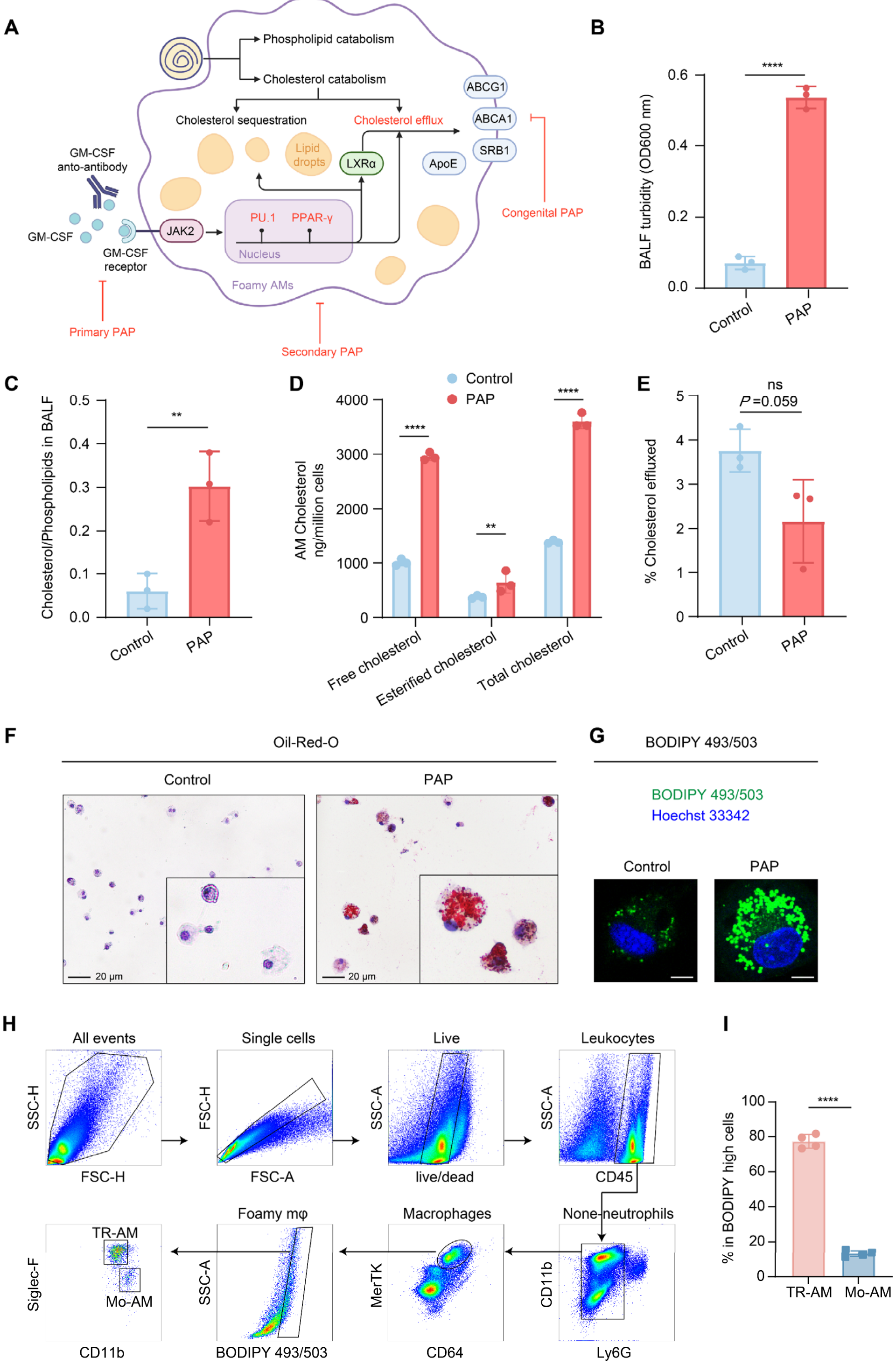

Supplementary Figure 1. Dysregulated lipid metabolism in PAP-AMs.

(A) Schematic illustration of AM alterations across different PAP subtypes. (B) Quantification of turbidity in BALF from PAP patients and control donors (n=3). (C) Cholesterol-to-phospholipid ratio in BALF samples from PAP patients compared to

control donors (n=3). (D) Intracellular cholesterol content in AMs isolated from PAP patients and control donors, as assessed by biochemical assays (n=3). (E) Quantification of cholesterol efflux from AMs using BODIPY-cholesterol assay (n=3). (F) Representative images of Oil Red O staining showing intracellular lipid droplet accumulation in AMs from PAP patients and control donors. Scale bar, 20 μm. (G) Confocal images of AMs stained with BODIPY 493/503 (green) to visualize neutral lipid droplets; nuclei were counterstained with Hoechst 33342 (blue). Scale bar, 5 μm. (H) Representative gating strategy showing the analysis of $BODIPY^{high}$ lipid-laden cells. Viable $CD45^{+}$ $Ly6G^{-}$ $CD64^{+}MerTK^{+}$ macrophages were first gated for $BODIPY^{high}$, followed by subset identification as TR-AMs ($Siglec\text{-}F^{high}CD11b^{low}$) or Mo-AMs ($Siglec\text{-}F^{low}CD11b^{high}$). (I) Quantification of the proportion of TR-AMs and Mo-AMs within the $BODIPY^{high}$ population (n = 4). Statistical comparisons were performed using a two-tailed unpaired t-test; p-values are indicated. (*$p < 0.05$, **$p < 0.01$, ***$p < 0.001$, ****$p < 0.0001$, ns, not significant)

Supplementary figure 2

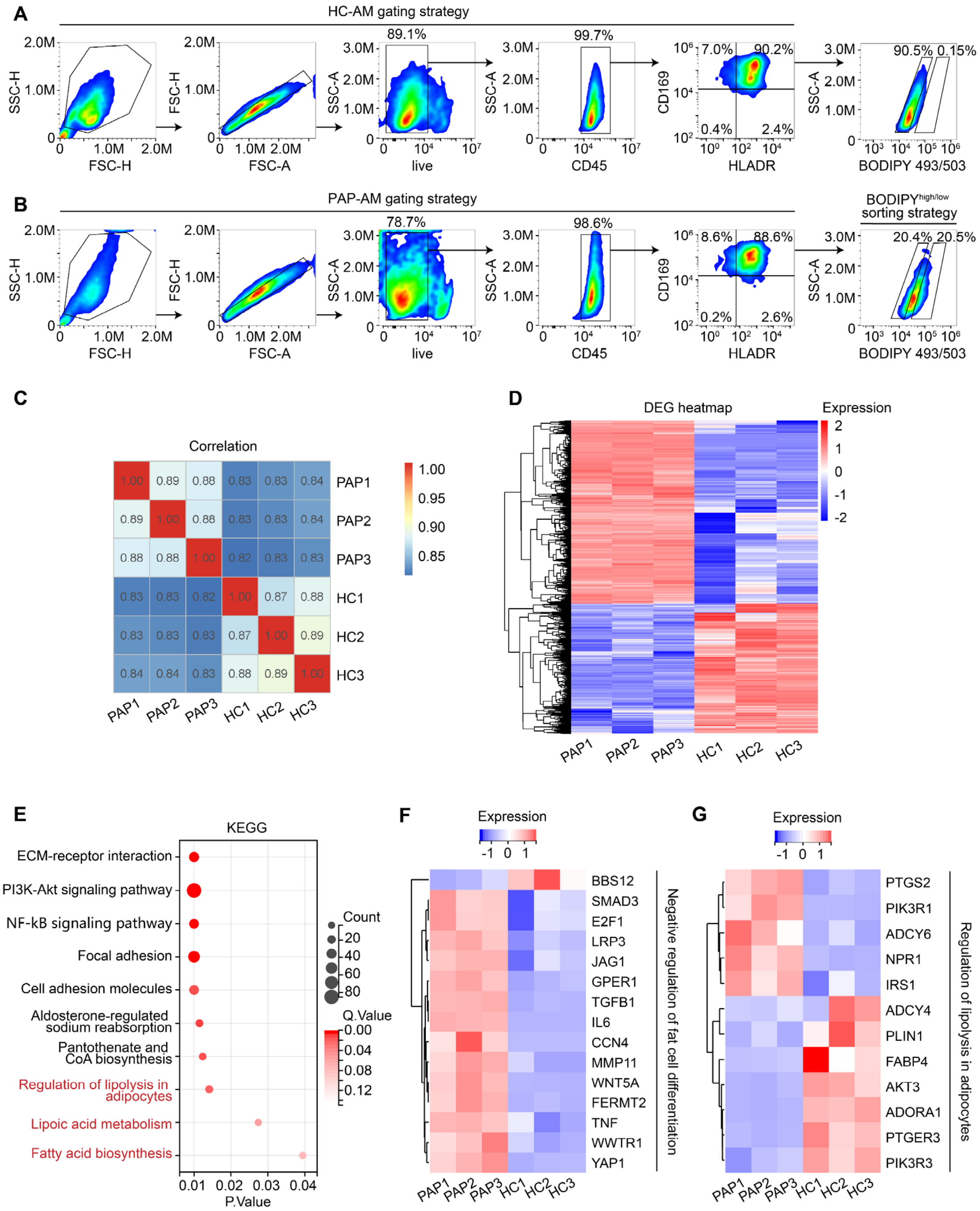

Supplementary Figure 2. Transcriptomic analysis of AMs in PAP patients and control donors.

(A, B) Gating strategy for AM isolation and sorting. Representative flow cytometry plots showing the sorting strategy for HC-AMs (A) and PAP-AMs (B). Viable singlet cells were gated for the AM lineage ($CD45^{+}HLA\text{-}DR^{+}CD169^{+}$). For PAP samples (B), cells were further sorted into $BODIPY^{high}$ and $BODIPY^{low}$ populations based on sample-specific top/bottom 20% fluorescence thresholds. (C) Correlation heatmap

showing transcriptomic similarity across groups. (D) Hierarchical clustering heatmap of differentially expressed genes. (E) KEGG pathway enrichment analysis of differentially expressed genes shown as a bubble plot. (F, G) Heatmaps showing the expression patterns of gene sets related to lipid metabolism across different groups.

Supplementary figure 3

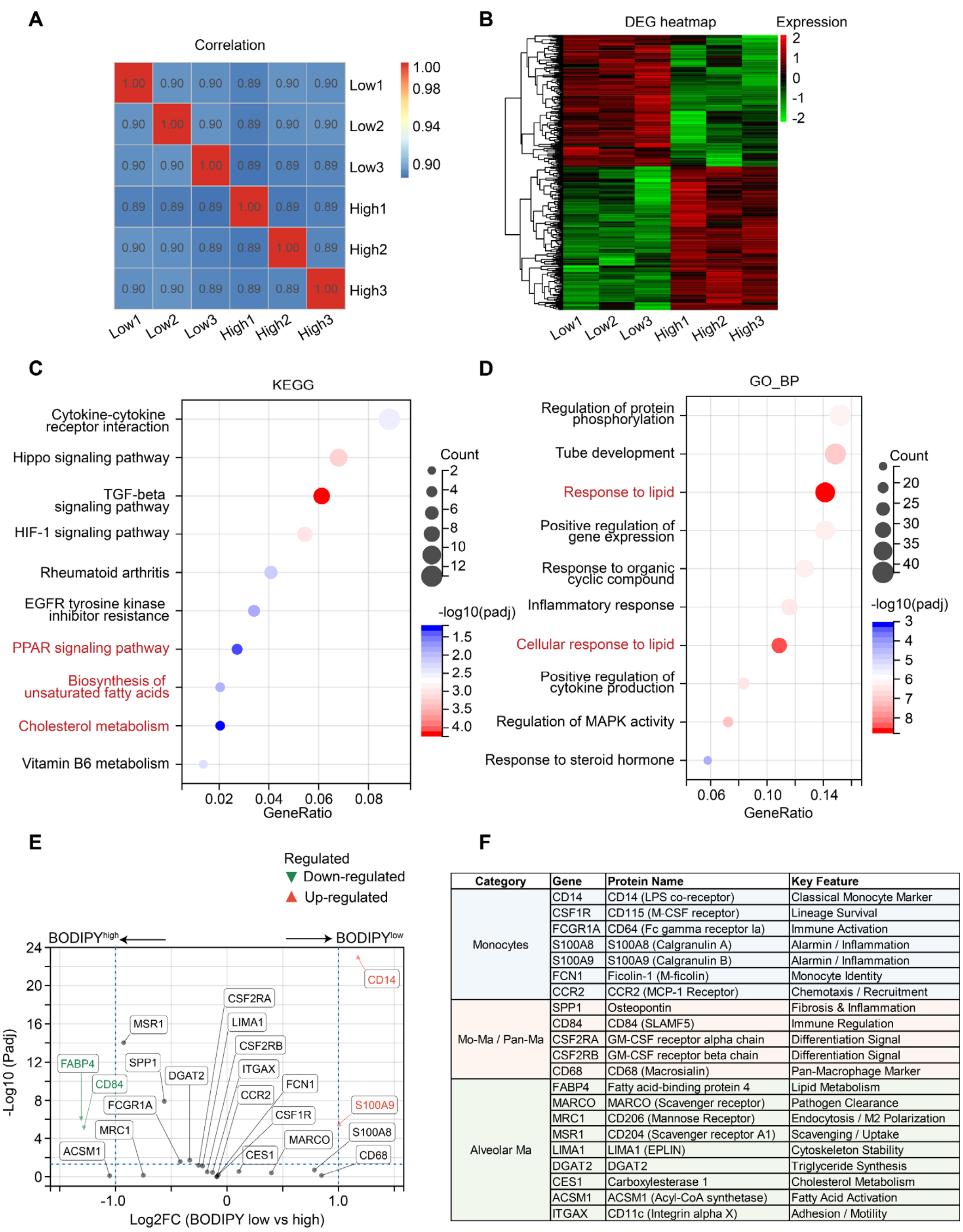

Category	Gene	Protein Name	Key Feature
Monocytes	CD14	CD14 (LPS co-receptor)	Classical Monocyte Marker
	CSF1R	CD115 (M-CSF receptor)	Lineage Survival
	FCGR1A	CD64 (Fc gamma receptor Ia)	Immune Activation
	S100A8	S100A8 (Calgranulin A)	Alarmin / Inflammation
	S100A9	S100A9 (Calgranulin B)	Alarmin / Inflammation
	FCN1	Ficolin-1 (M-ficolin)	Monocyte Identity
	CCR2	CCR2 (MCP-1 Receptor)	Chemotaxis / Recruitment
Mo-Ma / Pan-Ma	SPP1	Osteopontin	Fibrosis & Inflammation
	CD84	CD84 (SLAMF5)	Immune Regulation
	CSF2RA	GM-CSF receptor alpha chain	Differentiation Signal
	CSF2RB	GM-CSF receptor beta chain	Differentiation Signal
	CD68	CD68 (Macrosialin)	Pan-Macrophage Marker
Alveolar Ma	FABP4	Fatty acid-binding protein 4	Lipid Metabolism
	MARCO	MARCO (Scavenger receptor)	Pathogen Clearance
	MRC1	CD206 (Mannose Receptor)	Endocytosis / M2 Polarization
	MSR1	CD204 (Scavenger receptor A1)	Scavenging / Uptake
	LIMA1	LIMA1 (EPLIN)	Cytoskeleton Stability
	DGAT2	DGAT2	Triglyceride Synthesis
	CES1	Carboxylesterase 1	Cholesterol Metabolism
	ACSM1	ACSM1 (Acyl-CoA synthetase)	Fatty Acid Activation
	ITGAX	CD11c (Integrin alpha X)	Adhesion / Motility

Supplementary Figure 3. Transcriptomic analysis of BODIPY$^{high/low}$AMs in PAP patients.

(A) Correlation heatmap showing transcriptomic similarity across sorted groups. (B) Hierarchical clustering heatmap of differentially expressed genes. (C) KEGG pathway enrichment analysis of differentially expressed genes shown as a bubble plot. (D) GO enrichment analysis of differentially expressed genes presented as a bubble plot. (E) Volcano plot showing the differential expression of monocyte, AM and mo-Ma marker genes between BODIPY^{high} and BODIPY^{low} populations. (F) Annotation of selected monocyte, AM and mo-Ma marker genes shown in (E).

Supplementary figure 4

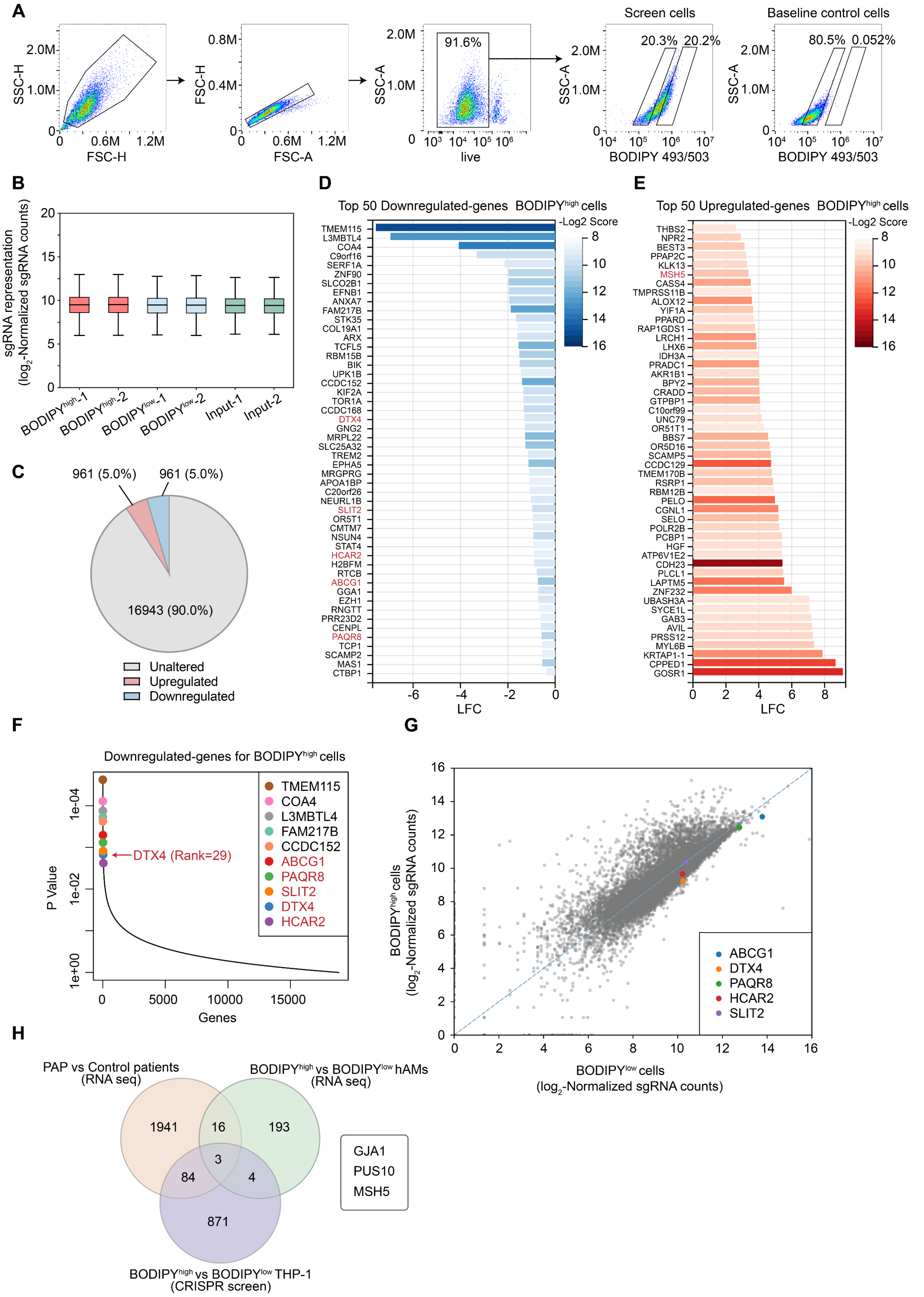

66

67 **Supplementary Figure 4. CRISPR screen identifies regulators of cholesterol efflux**
68 **in AMs.**

(A) CRISPR screen sorting strategy. Viable singlet THP-1 cells were gated and sorted into BODIPY^{high} (top 20%) and BODIPY^{low} (bottom 20%) populations for subsequent library sequencing. (B) Box plot showing the distribution of sgRNA counts across different experimental groups. (C) Pie chart illustrating the proportion of significantly upregulated and downregulated genes in the BODIPY^{high} group. (D, E) Bar plots displaying the top 50 upregulated (D) and downregulated (E) genes in the BODIPY^{high} group. (F) Hockey-stick plot depicting the statistical significance (*P* values) of top down-regulated genes in BODIPY^{high} macrophages, as identified by MAGeCK analysis. (G) Scatter plot comparing normalized sgRNA counts between BODIPY^{high} and BODIPY^{low} groups. (H) Venn diagram showing the overlap of upregulated genes identified across three independent transcriptomic datasets.

Supplementary figure 5

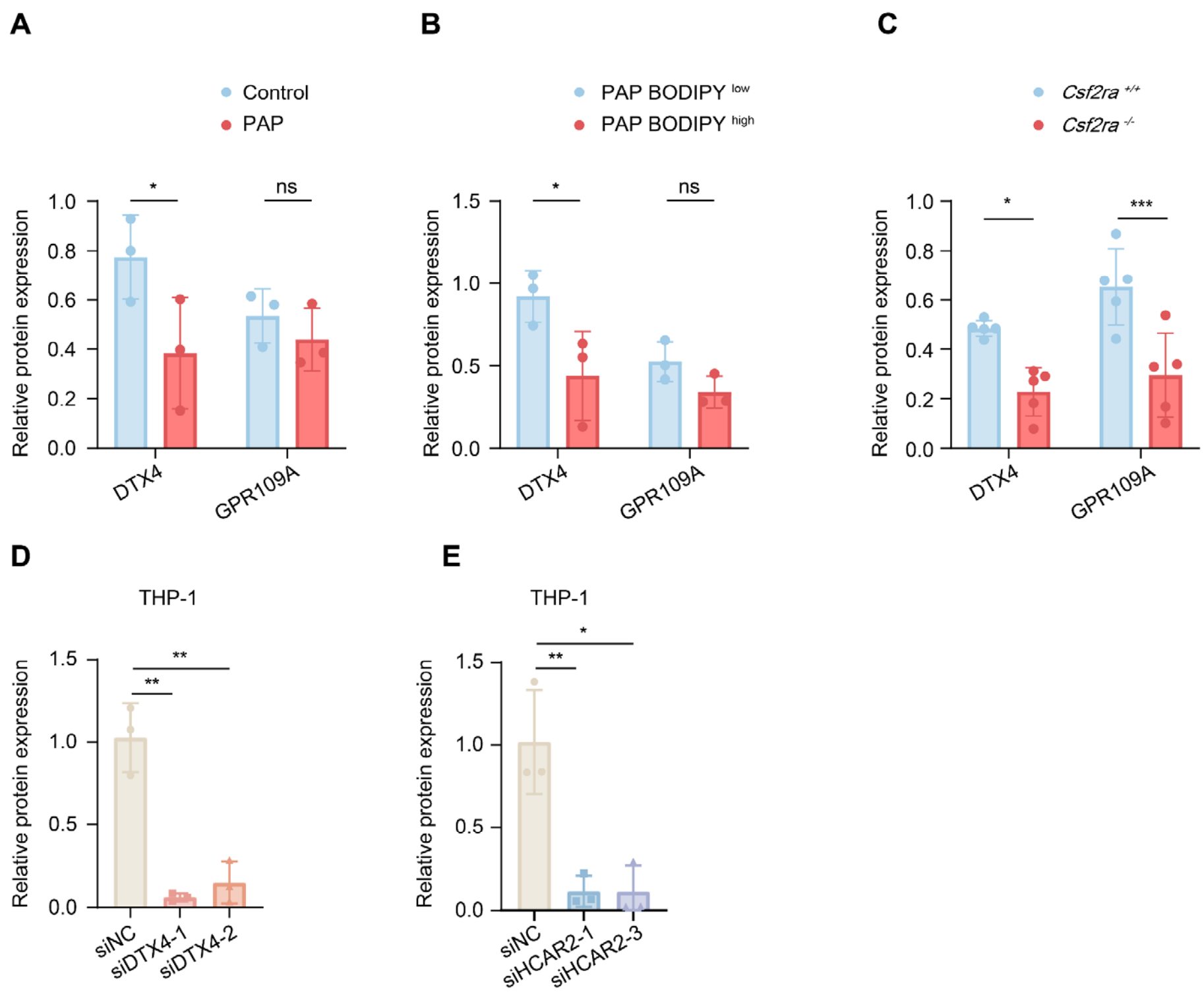

Supplementary Figure 5. Validation of DTX4 as the regulator of cholesterol efflux.

(A-C) Quantification of Western blot analysis of the indicated proteins in AMs from PAP patients and healthy donors (A), BODIPY^{high} and BODIPY^{low} PAP-AMs (B), and AMs from *Csf2ra*$^{+/+}$ and *Csf2ra*$^{-/-}$ mice (C). GAPDH as a loading control (n = 3-5 biological replicates). (D, E) Quantification of Western blot analysis of the indicated proteins in THP-1 cells following siRNA-mediated knockdown of DTX4 (D) and

HCAR2 (E). GAPDH as a loading control (n = 3 biological replicates). Statistical significance was determined by one-way ANOVA with Tukey's post hoc test or a two-tailed unpaired t-test (*$p < 0.05$, **$p < 0.01$, ***$p < 0.001$, ns, not significant).

Supplementary figure 6

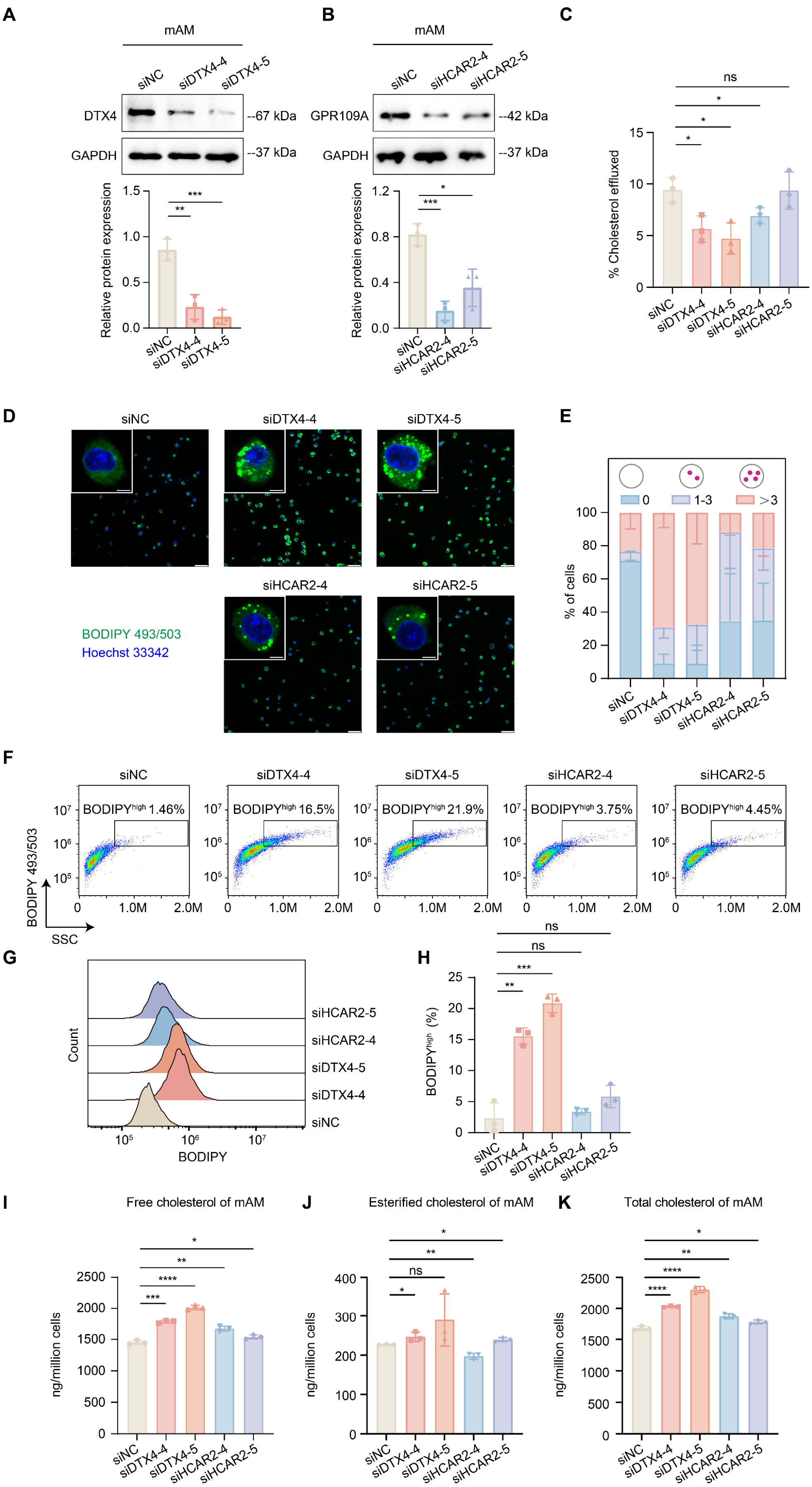

Supplementary Figure 6. DTX4 knockdown impairs cholesterol efflux in primary mAMs *in vitro*.

(A, B) Western blot analysis (A) and quantification (B) of indicated proteins in differently treated mAMs (n = 3 biological replicates). GAPDH was used as a loading control. (C) Quantification of cholesterol efflux using the BODIPY-cholesterol assay (n = 3 biological replicates). (D, E) Representative images (D) and quantification (E) of BODIPY staining showing intracellular lipid droplet accumulation under different treatment conditions (n = 3 biological replicates). Scale bar, 5 μm (up), 50 μm (down). (F-H) Flow cytometry analysis showing BODIPY fluorescence in mAMs across treatment groups, including density plots (F), ridgeline plot (G)and quantification of BODIPY^{high} cell proportions (H) (n = 3 biological replicates). (I-K) Quantification of intracellular free cholesterol (I), cholesteryl esters (J), and total cholesterol (K) in mAMs under different treatments (n = 3 biological replicates). Statistical analysis was performed using one-way ANOVA followed by Tukey's post hoc test for multiple comparisons (*p < 0.05, **p < 0.01, ***p < 0.001, ****p < 0.0001, ns, not significant)

Supplementary figure 7

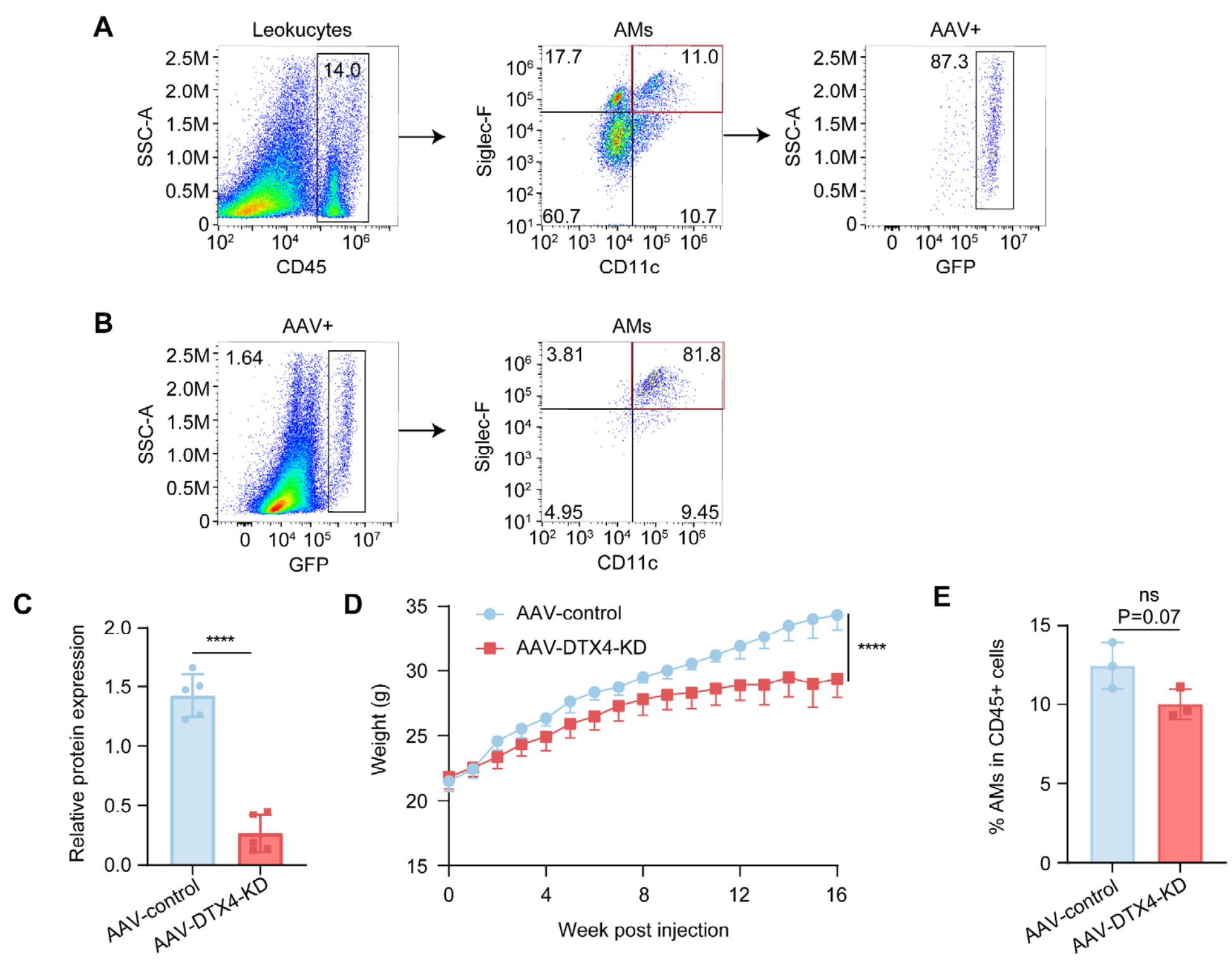

Supplementary Figure 7. Evaluation of AAV-mediated DTX4 knockdown

efficiency and physiological outcomes in mice.

(A, B) Flow cytometry gating strategy to identify AMs from lung tissues of AAV-treated mice. (C) Quantification of Western blot analysis of the indicated proteins in AMs isolated from mice treated with different AAV constructs. GAPDH was used as a loading control (n=5). (D) Body weight curves of mice in each treatment group monitored over time (n=8). (E) Flow cytometric analysis of the proportion of AMs in lung tissues following AAV treatment (n=3). Statistical comparisons were performed using a two-tailed unpaired t-test; p-values are indicated (****p < 0.0001, ns, not significant).

Supplementary figure 8

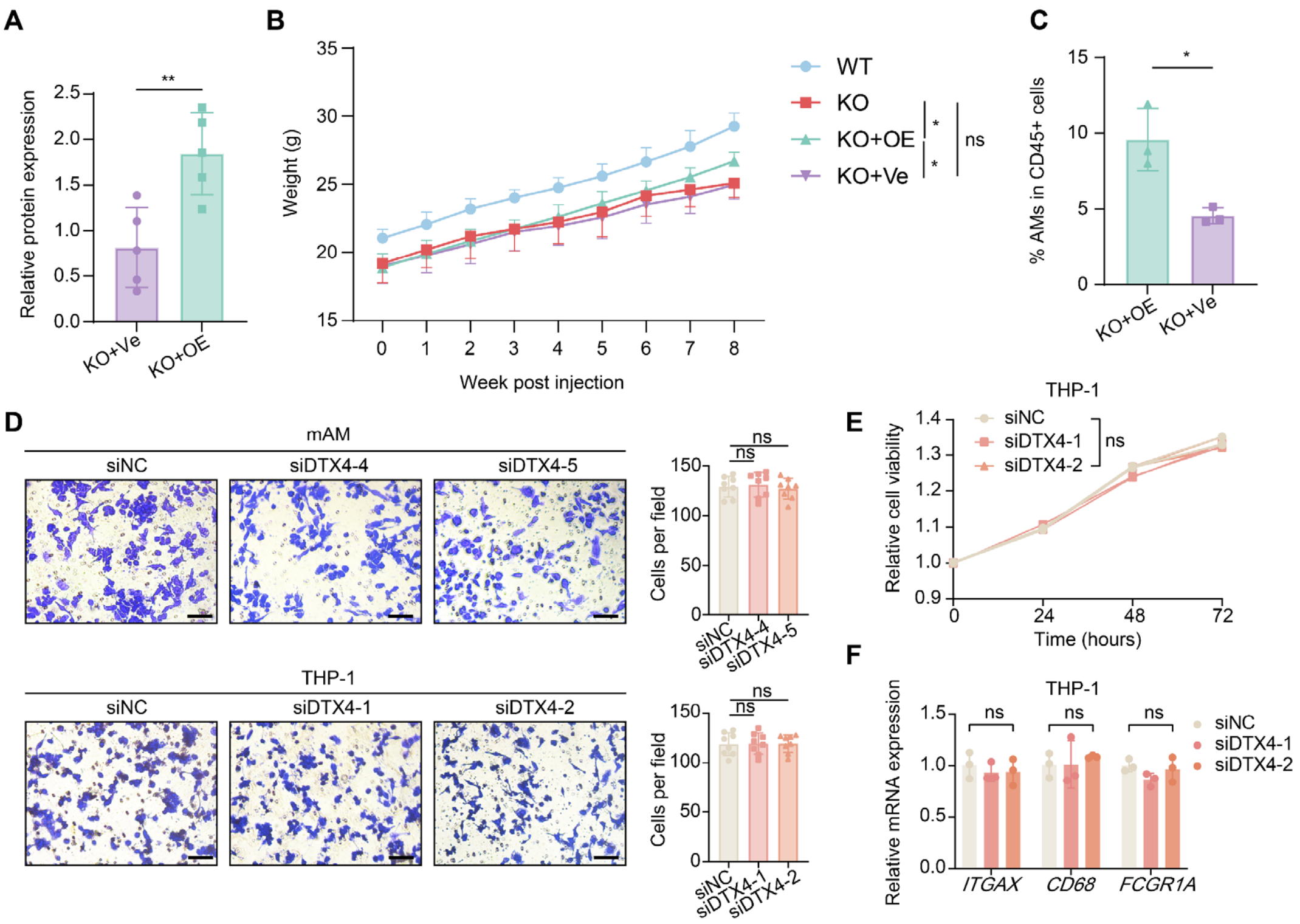

Supplementary Figure 8. Evaluation of AAV transduction efficiency, physiological parameters, and cellular functions following DTX4 modulation.

(A) Quantification of Western blot analysis of the indicated proteins in AMs isolated from mice treated with different AAV constructs (n=5). GAPDH was used as a loading control. (B) Body weight curves of mice in each treatment group over time (n=8). (C) Flow cytometric analysis of the proportion of AMs in lung tissues following AAV

treatment (n=3). (D) Representative transwell migration images and quantification of mAMs (up) and THP-1 cells (down) following DTX4 silencing (n = 8). Scale bar, 50 μm. (E) Cell viability analysis of DTX4-KD THP-1 cells assessed by CCK-8 assay (n = 3 biological replicates). (F) Relative mRNA expression levels of indicated genes in DTX4-knockdown THP-1 cells (n = 3 biological replicates). Statistical comparisons were made using one-way ANOVA followed by Tukey's post hoc test for multiple comparisons, or a 2-tailed unpaired Student's t-test for comparisons between 2 groups (*p < 0.05, **p < 0.01, ns, not significant).

Supplementary figure 9

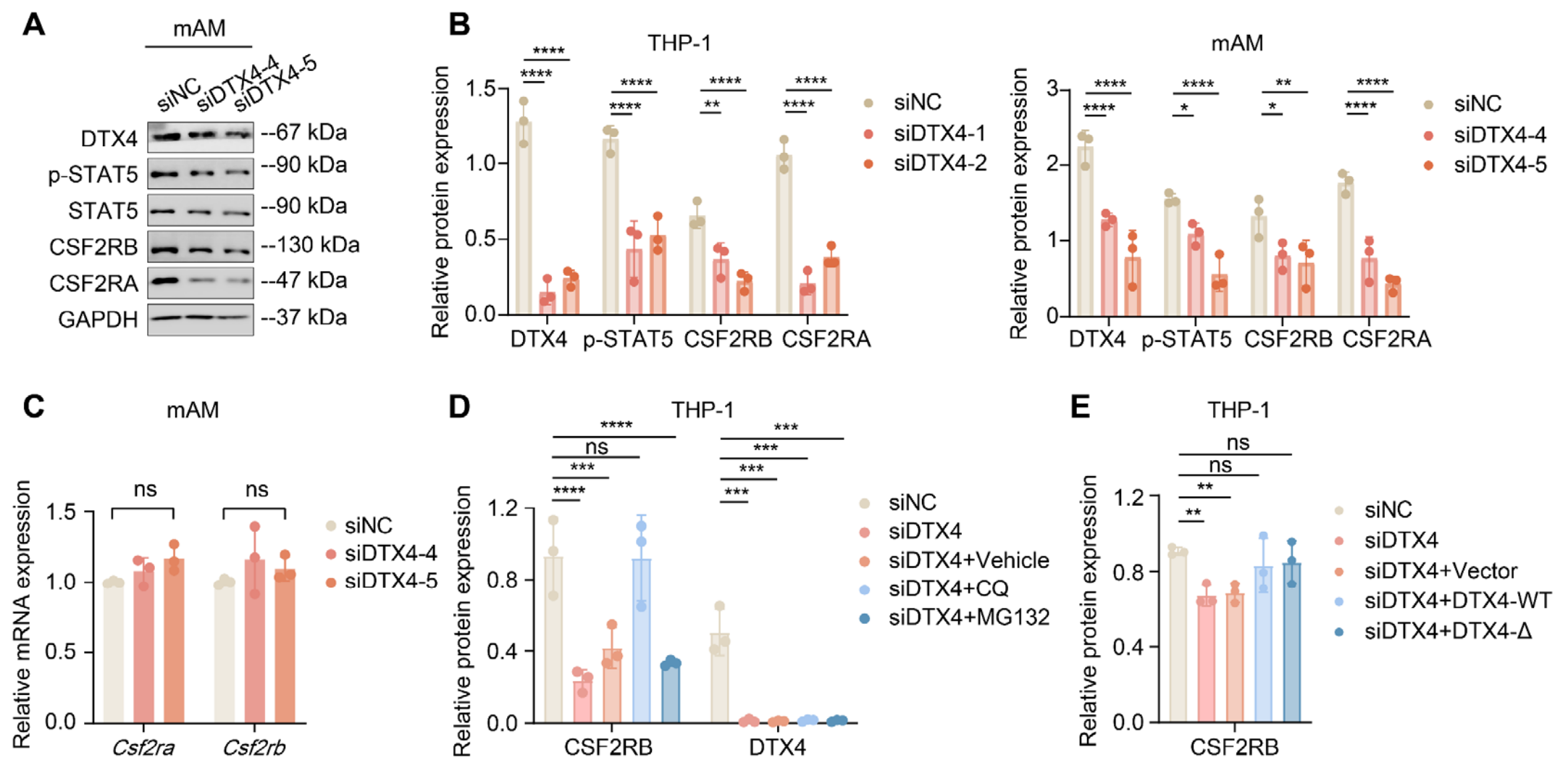

Supplementary Figure 9. DTX4 protects CSF2RB from lysosomal degradation.

(A, B) Western blot analysis (A) and quantification (B) of CSF2RA, CSF2RB, and signaling intermediates in DTX4-KD mAMs and THP-1 cells (n = 3 biological replicates). (C) RT-qPCR analysis of *Csf2ra* and *Csf2rb* mRNA levels in DTX4-KD mAMs (n = 3 biological replicates). (D, E) Quantification of indicated protein levels in DTX4-KD THP-1 cells following treatment with chloroquine (CQ, 50 μM) or MG132 (20 μM) (D), or upon re-expression of DTX4-WT or DTX4-Δ (E) (n = 3 biological replicates). Statistical analysis was performed using one-way ANOVA followed by Tukey's post hoc test for multiple comparisons (*p < 0.05, **p < 0.01, ***p < 0.001, ****p < 0.0001; ns, not significant).

Supplementary figure 10

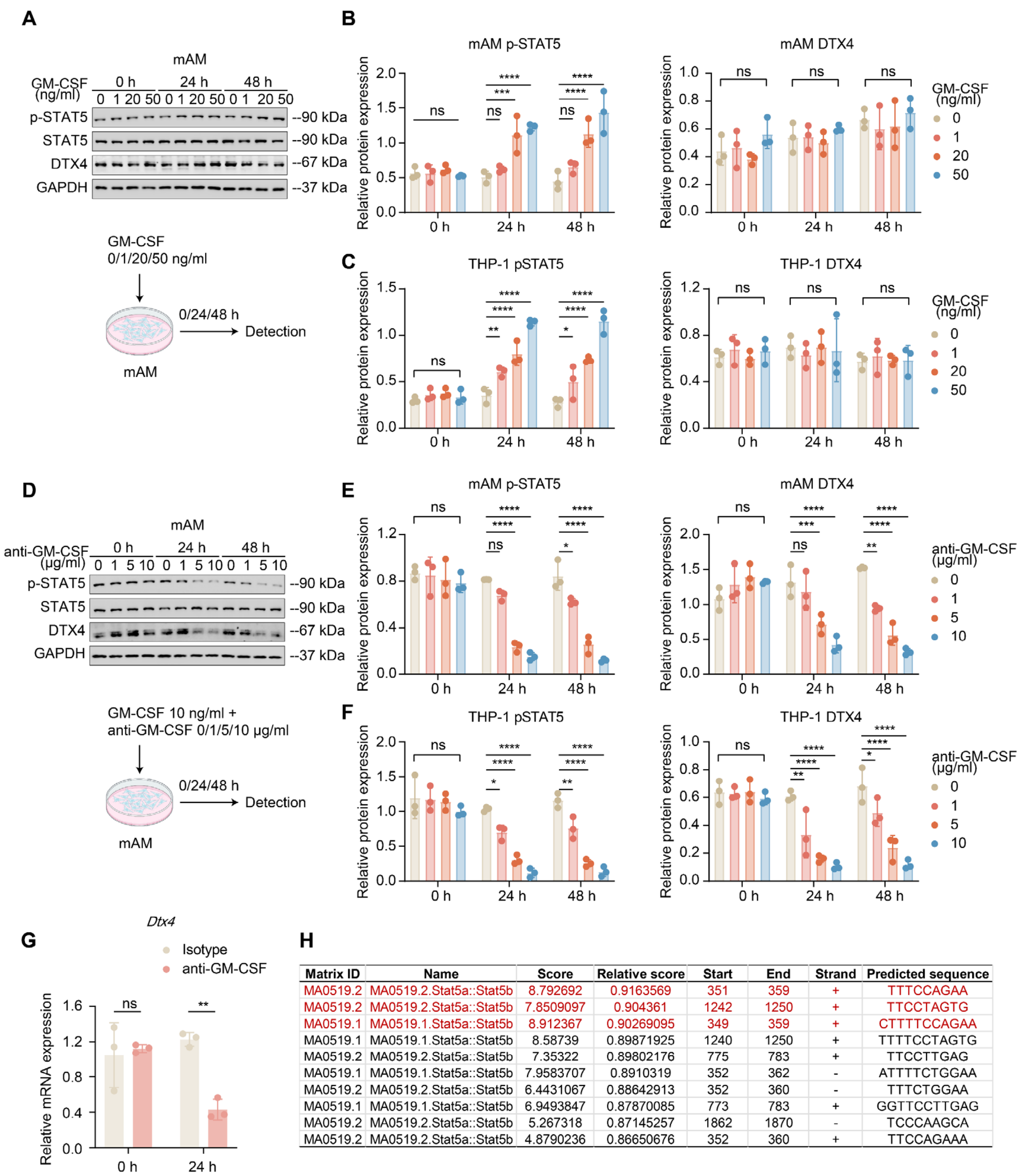

Matrix ID	Name	Score	Relative score	Start	End	Strand	Predicted sequence
MA0519.2	MA0519.2.Stat5a::Stat5b	8.792692	0.9163569	351	359	+	TTTCCAGAA
MA0519.2	MA0519.2.Stat5a::Stat5b	7.8509097	0.904361	1242	1250	+	TTCCTAGTG
MA0519.1	MA0519.1.Stat5a::Stat5b	8.912367	0.90269095	349	359	+	CTTTTCCAGAA
MA0519.1	MA0519.1.Stat5a::Stat5b	8.58739	0.89871925	1240	1250	+	TTTTCCTAGTG
MA0519.2	MA0519.2.Stat5a::Stat5b	7.35322	0.89802176	775	783	+	TTCCTTGAG
MA0519.1	MA0519.1.Stat5a::Stat5b	7.9583707	0.8910319	352	362	-	ATTTTCTGGAA
MA0519.2	MA0519.2.Stat5a::Stat5b	6.4431067	0.88642913	352	360	-	TTTCTGGAA
MA0519.1	MA0519.1.Stat5a::Stat5b	6.9493847	0.87870085	773	783	+	GGTTCCTTGAG
MA0519.2	MA0519.2.Stat5a::Stat5b	5.267318	0.87145257	1862	1870	-	TCCCAAGCA
MA0519.2	MA0519.2.Stat5a::Stat5b	4.8790236	0.86650676	352	360	+	TTCCAGAAA

Supplementary Figure 10. GM-CSF signaling maintains DTX4 expression.

(A–C) Western blot analysis (A) and quantification (B, C) of DTX4 and downstream signaling proteins in mAMs and THP-1 cells following recombinant GM-CSF stimulation (n = 3 biological replicates). (D–F) Western blot analysis (D) and quantification (E, F) of DTX4 and downstream signaling proteins in mAMs and THP-1 cells treated with anti-GM-CSF neutralizing antibody (n = 3 biological replicates). (G) RT-qPCR analysis of *Dtx4* mRNA levels in mAMs upon GM-CSF neutralization (n = 3 biological replicates). (H) JASPAR prediction scores for high-confidence STAT5

binding sites within the DTX4 promoter region. Statistical analysis was performed using unpaired t-test or one-way ANOVA with Tukey's post hoc test (*$p < 0.05$, **$p < 0.01$, ***$p < 0.001$, ****$p < 0.0001$; ns, not significant).

Supplementary figure 11

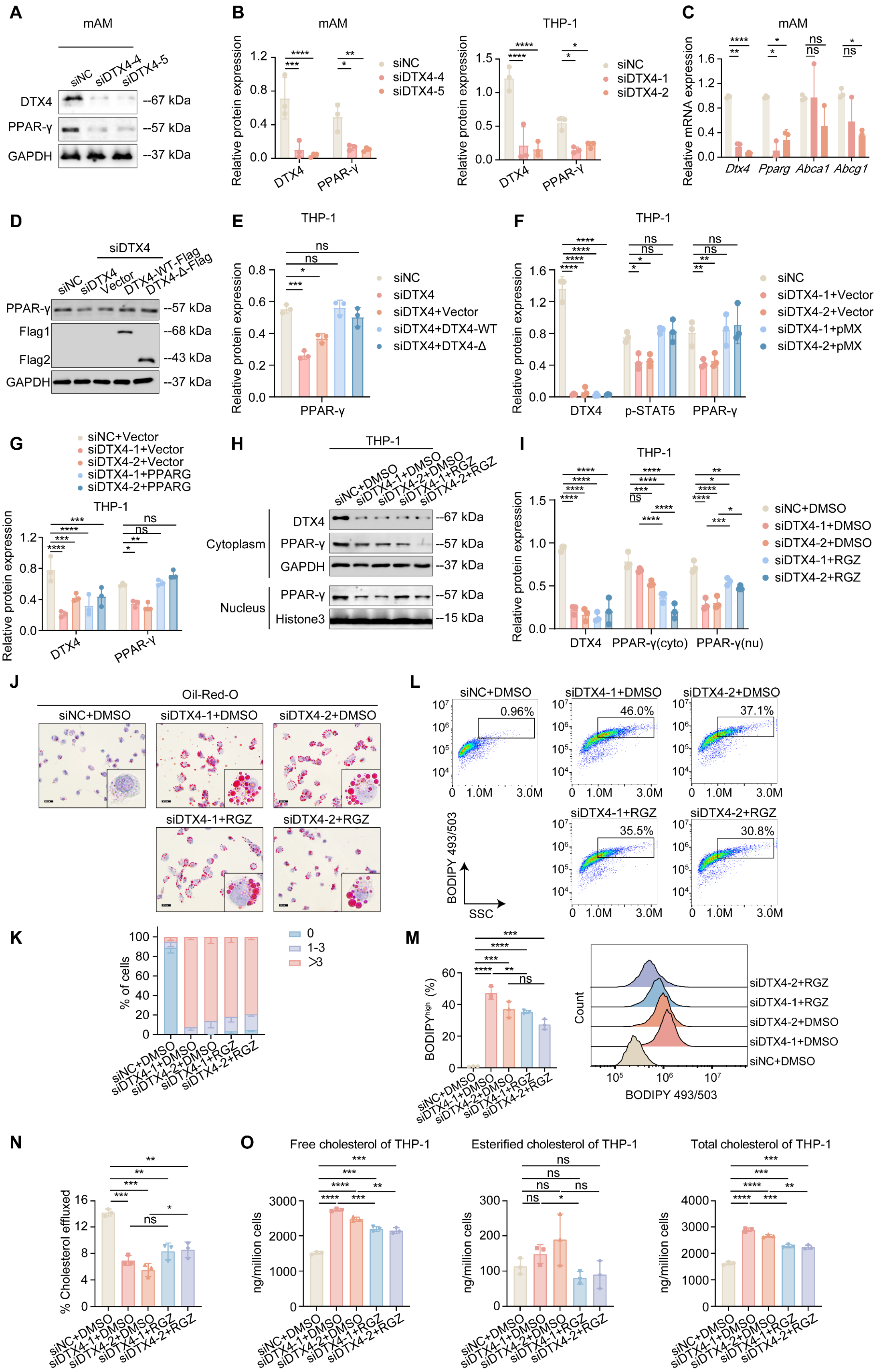

157

Supplementary Figure 11. DTX4 regulates PPAR-γ expression and cholesterol efflux via the GM-CSF–STAT5 axis.

(A, B) Western blot analysis (A) and quantification (B) of indicated proteins in DTX4-knockdown mAMs and THP-1 cells (n = 3 biological replicates). (C) RT-qPCR analysis of indicated genes in DTX4-knockdown mAMs (n = 3 biological replicates). (D–I) Quantification of Western blot analysis for indicated proteins in DTX4-knockdown THP-1 cells following: rescue with DTX4-WT or DTX4-Δ (E, from blot D); overexpression of constitutively active STAT5 (pMX-STAT5A1*6) (F); overexpression of PPAR-γ (G); or treatment with rosiglitazone (I, from blot H) (n = 3 biological replicates). (J–M) Assessment of intracellular lipid accumulation in DTX4-knockdown THP-1 cells treated with rosiglitazone. Shown are representative Oil Red O staining (J) with quantification (K), and flow cytometric analysis of BODIPY 493/503 fluorescence presented as density plots (L) and quantification/ridgeline plots (M) (n = 3 biological replicates). Scale bar, 100 µm. (N, O) Analysis of BODIPY-cholesterol efflux capacity (N) and intracellular cholesterol content (O) under the indicated conditions (n = 3 biological replicates). Statistical analysis was performed using one-way ANOVA followed by Tukey's post hoc test for multiple comparisons (*$p < 0.05$, **$p < 0.01$, ***$p < 0.001$, ****$p < 0.0001$, ns, not significant).

Supplementary Table 1. Clinical Characteristics of PAP Patients and Control Donors

**Variable**	**PAP Patients**	**Control Donors**
Number of subjects	6	6
Age (mean ± SD, years)	50.5 ± 12.6	49.7 ± 8.5
Sex (Male / Female)	3 / 3	4 / 2
PAP subtype		
– Autoimmune PAP (GM-CSF autoantibody positive)	4	NA
– Hereditary PAP (CSF2RA / CSF2RB mutation)	2	NA
BALF samples used for RNA-seq 1 (PAP vs Con)	Yes (aPAP, n = 3)	Yes (n = 3)
BALF samples used for RNA-seq 2 (BODIPY ^{high} vs BODIPY ^{low})	Yes (aPAP, n = 3)	No
BALF samples used for experimental validation	Yes (aPAP, n = 1; hPAP, n = 2)[*]	Yes (n = 3)[*]

[*] Note: Samples used for experimental validation were independent from those used for RNA sequencing.

Supplementary Table 2. Related to Methods, primer sequences.

Primer Name	Sequence (5' to 3')
Csfr2a-KO-F1	AAGTGGGGACTGGAGAAAAGGAA
Csfr2a-KO-R1	GACATCAATGTCGTTAAACATGCC
Csfr2a-KO-R2	CACCTAGCGTCACTAACCCAGAA
H-*DTX4*-F	GAATGGCTGAACGAGCACG
H-*DTX4*-R	CCGAGGAGGGGTCGTAGTA
H-*SLIT2*-F	GCGAAGCTATACAGGCTTGAT
H-*SLIT2*-R	TGCAGTCGAAAAGTCCTAAGTTT
H-*PAQR8*-F	CAGCAGGTCTGGCTTTTATCC
H-*PAQR8*-R	GGGAGAGCGTACAGATGGACA
H-*HCAR2*-F	ATGTTGGCTATGAACCGCCAG
H-*HCAR2*-R	GCTGCTGTCCGATTGGAGA
M-*Dtx4*-F	TGTGCCTGTGAAAAACTTGAATG
M-*Dtx4*-R	TGGGATGGACTTTATCTCACTCT
M-*Slit2*-F	GGCAGACACTGTCCCTATCG
M-*Slit2*-R	ATCTATCTTCGTGATCCTCGTGA
M-*Paqr8*-F	ATGACGACTGCCATCCTAGAG
M-*Paqr8*-R	GCTCCCTAAAGAGCTGGGG
M-*Hcar2*-F	CTGGAGGTTCGGAGGCATC
M- *Hcar2*-R	TCGCCATTTTTGGTCATCATGT
H-*PPARG*-F	GGGATCAGCTCCGTGGATCT

H-*PPARG*-R	TGCACTTTGGTACTCTTGAAGTT
M-*Pparg*-F	TCGCTGATGCACTGCCTATG
M-*Pparg*-R	GAGAGGTCCACAGAGCTGATT
H-*ABCA1*-F	ACCCACCCTATGAACAACATGA
H-*ABCA1*-R	GAGTCGGGTAACGGAAACAGG
M-*Abca1*-F	GCTTGTTGGCCTCAGTTAAGG
M-*Abca1*-R	GTAGCTCAGGCGTACAGAGAT
H-*ABCG1*-F	CGTGCGCTTTGTGCTGTTT
H-*ABCG1*-R	CCACTGTAGGTACGTGGGGAT
M-*Abcg1*-F	CTTTCCTACTCTGTACCCGAGG
M-*Abcg1*-R	CGGGGCATTCCATTGATAAGG
H-*ITGAX*-F	GGGATGCCGCCAAAATTCTC
H-*ITGAX*-R	ATTGCATAGCGGATGATGCCT
H-*CD68*-F	TGGGGCAGAGCTTCAGTTG
H-*CD68*-R	TGGGGCAGGAGAAACTTTGC
H-*FCGR1A*-F	GCATGGGAAAGCATCGCTAC
H-*FCGR1A*-R	GCAAGAGCAACTTTGTTTCACA
H-*CSF2RA*-F	AGGAGGGAGATCCGGTGTC
H-*CSF2RA*-R	TTGCGAGACGTTAATCCTGAC
H-*CSF2RB*-F	CCATCCCTCAACGTGACCAAG
H-*CSF2RB*-R	GGCCGTGTCTTTCCTGTACTG
M-*Csf2ra*-F	CTGCTCTTCTCCACGCTACTG

M-*Csf2ra*-R	GAGACTCGCCGGTGTATCC
M-*Csf2rb*-F	AAAAACAGCCAGTGTCCTGTG
M-*Csf2rb*-R	GATGCTGACGTTCTTGGGAAG
H-*ACTB*-F	CATGTACGTTGCTATCCAGGC
H-*ACTB*-R	CTCCTTAATGTCACGCACGAT
M-*Actb*-F	GGCTGTATTCCCCTCCATCG
M-*Actb*-R	CCAGTTGGTAACAATGCCATGT

Supplementary Table 3. Related to Methods, Antibodies, Chemicals, and Kits.

**Antibodies, Chemicals, Kits**	**Source**	**Catalogue#**
DTX4	Abmart	TD12963
GRP109A	Abmart	PA6195
PPARγ	Cell Signaling Technology	2435
Flag	Cell Signaling Technology	14793
HA	Abmart	M20003
CSF2RA	Abmart	PA3877
CSF2RB	Abmart	MK53542
CSF2RB (For IP)	Abmart	MT13790
STAT5	Cell Signaling Technology	94205
Phospho-Stat5 (Tyr694)	Cell Signaling Technology	9351
GAPDH	Cell Signaling Technology	2118
Histone H3	Cell Signaling Technology	9715

APC anti-mouse CD45	Biolegend	103112
PE anti-mouse Siglec-F	BD Pharmingen	552126
PE-CY7 anti-mouse CD11c	BD Pharmingen	558079
PE-CY5 anti-mouse CD11b	BD Pharmingen	550993
FITC anti-human CD45	Biolegend	304006
APC anti-human HLA-DR	BD Pharmingen	559868
PerCP/Cyanine5.5 anti-human CD169	Biolegend	346020
ApoA1	Fitzgerald	118-30-1047S
Blasticidin S	Solarbio	B9300
BODIPY-Cholesterol	MCE	HY-125746
BODIPY 493/503	MCE	HY-W090090
cAMP	Sigma-Aldrich	C8988
Cholesterol	MCE	HY-N0322
Hoechst33342	Solarbio	C0031
Namilumab (anti-human GM-CSF antibody)	MCE	AMG-203
Anti-mouse GM-CSF antibody	SinoBiological	51048-RP01
oxLDL	Yeasen	20605ES10
PMA	Sigma-Aldrich	P1585
rhGM-CSF	Amoytop Biotech	NA
rmGM-CSF	R＆D systems	415-ML

Rosiglitazone	Sigma-Aldrich	R2408
Sandoz 58-035	Sigma-Aldrich	S9318
TGF-β1	R＆D systems	7666-MB
Amplex Red Cholesterol and Cholesteryl Ester Assay Kit	Beyotime	S0211
Phospholipids Assay Kit	Mlbio	M1243L96
Oil Red O staining kit	Beyotime	C0157
Mouse MCP-1 ELISA kit	Beyotime	PC125
Mouse GM-CSF ELISA kit	Mlbio	ml037645
Mouse M-CSF ELISA kit	Mlbio	ml002129
HiScript II Reverse Transcriptase kit	Vazyme	R323-01
ChamQ Universal SYBR qPCR Master Mix	Vazyme	Q711-02
