# Supplementary material for: CRISPR screening identifies DTX4 governing alveolar macrophage cholesterol efflux in pulmonary alveolar proteinosis
Source: JCI Insight. 2026 Jul 22;11(14):e201129. doi: 10.1172/jci.insight.201129 (PMC13461153; doi:10.1172/jci.insight.201129)

Fig. 2H

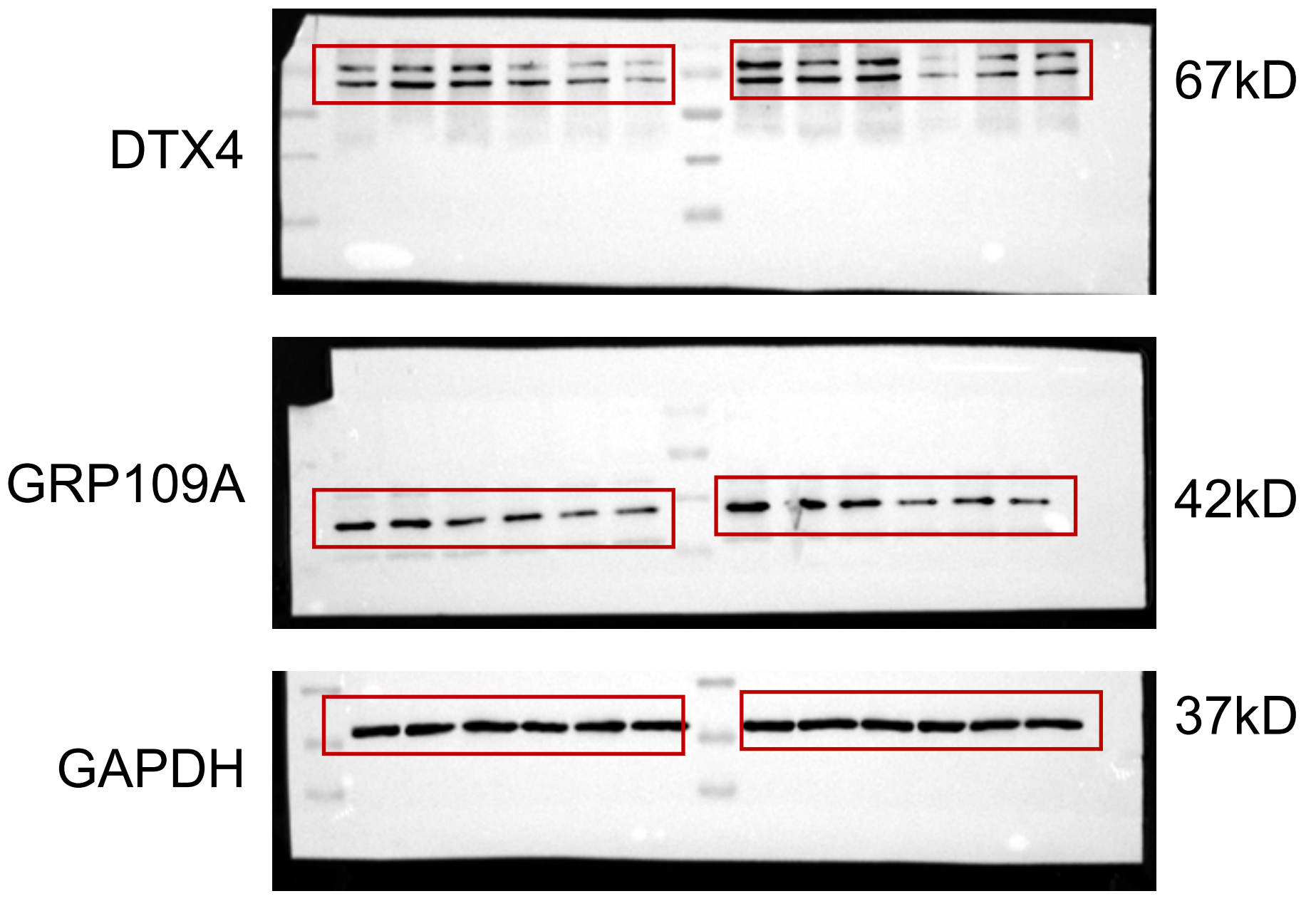
DTX4
67kD
GRP109A
42kD
GAPDH
37kD

Fig. 2I

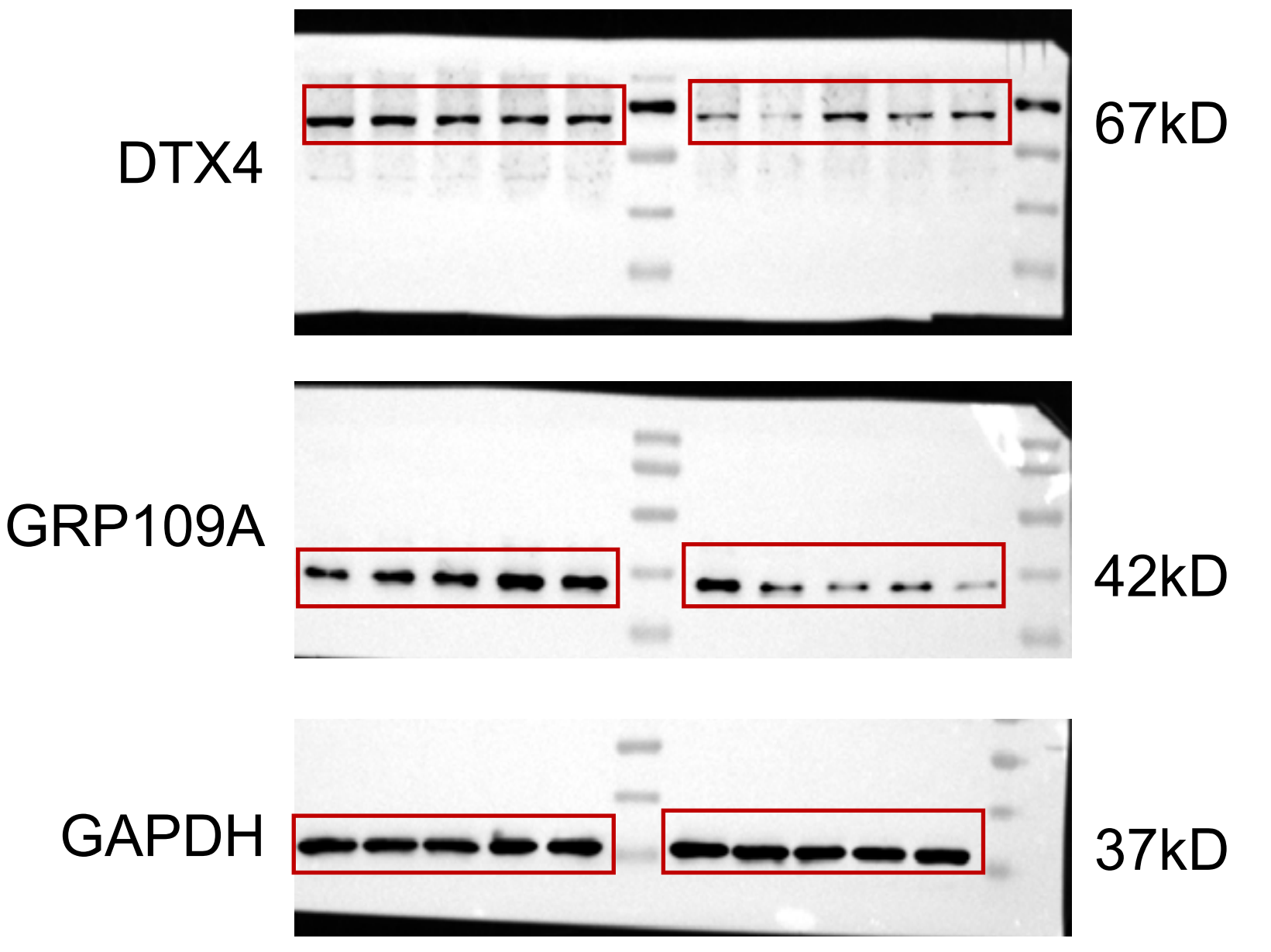
DTX4
67kD
GRP109A
42kD
GAPDH
37kD

Fig. 3A

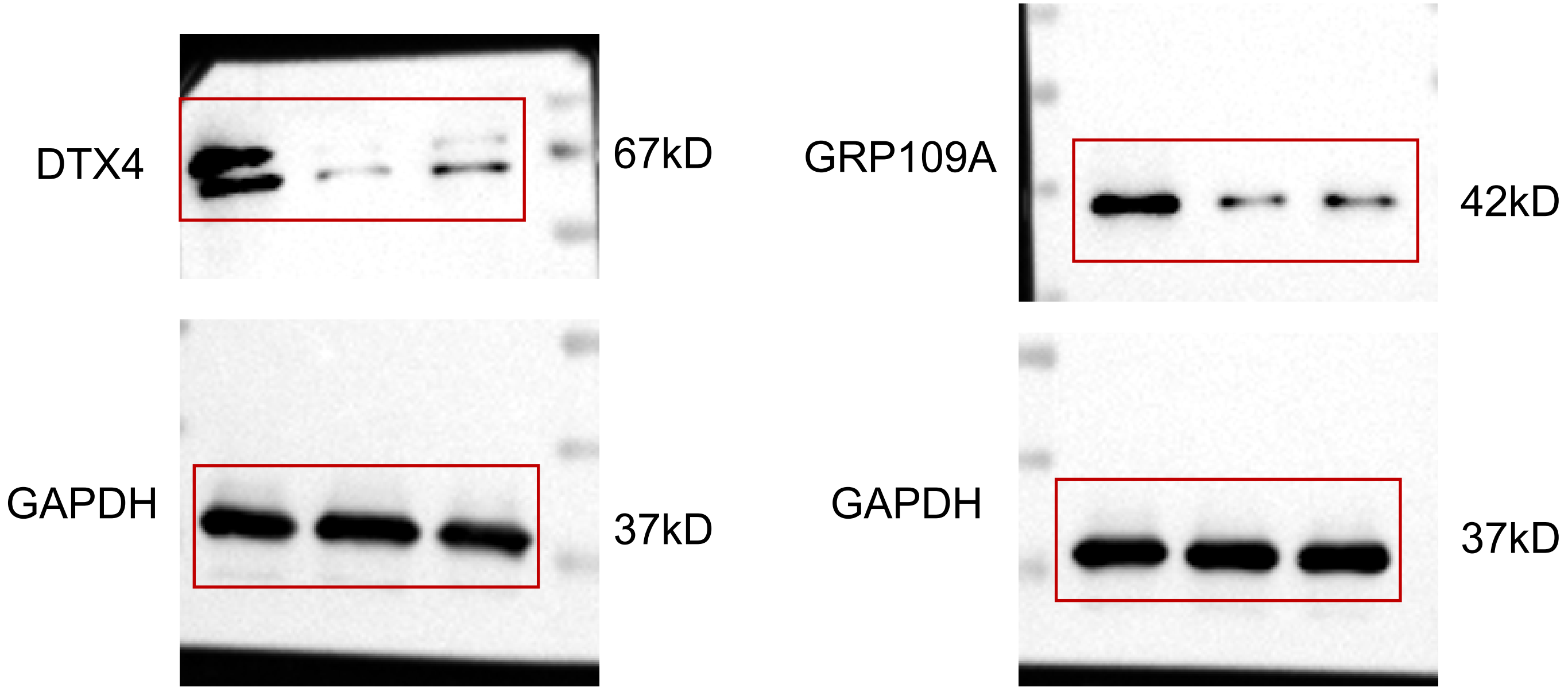

Fig. 4D

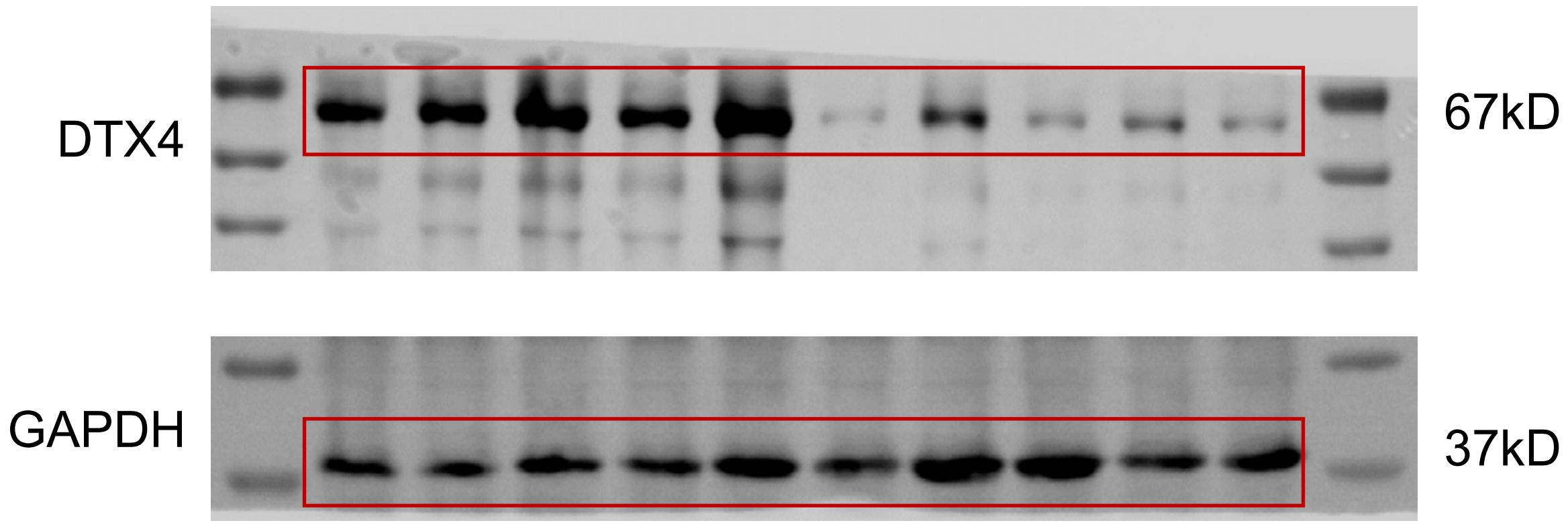

Fig. 5C

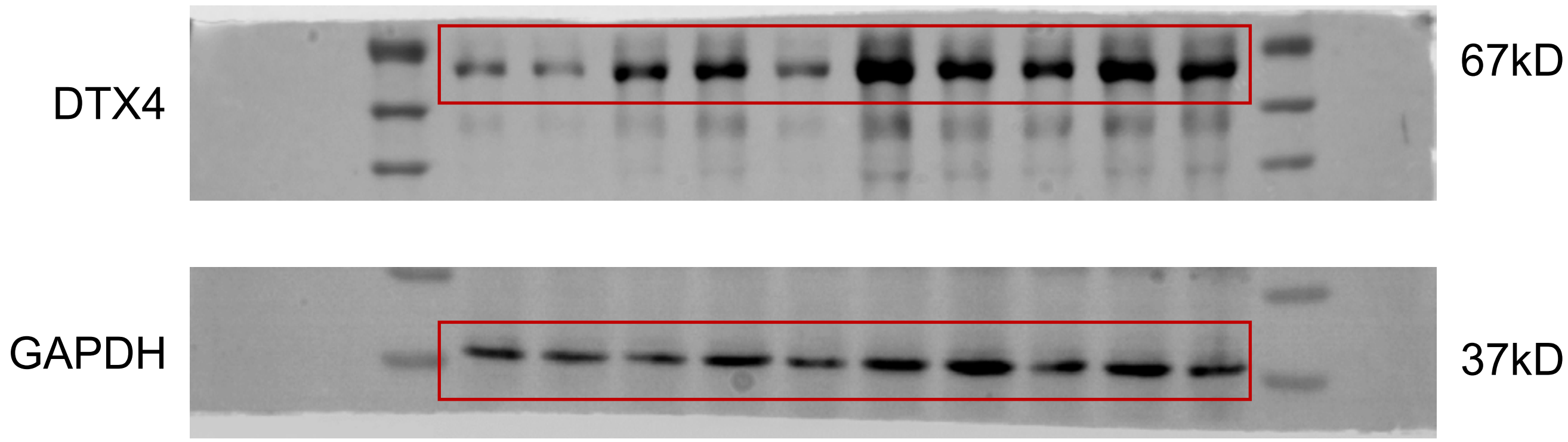

Fig. 6A

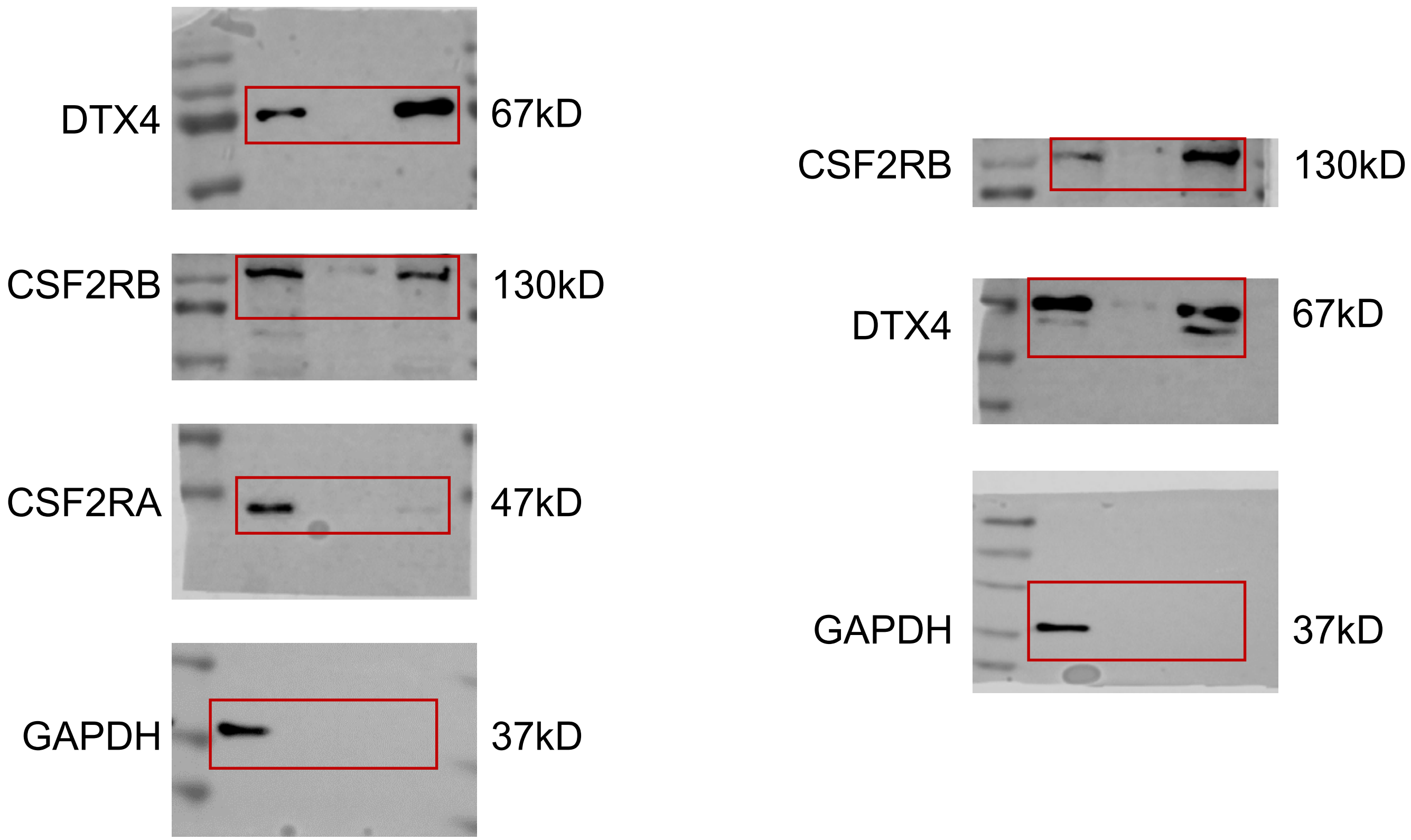

DTX4
67kD
CSF2RB
130kD
CSF2RA
47kD
GAPDH
37kD
CSF2RB
130kD
DTX4
67kD
GAPDH
37kD

Fig. 6C

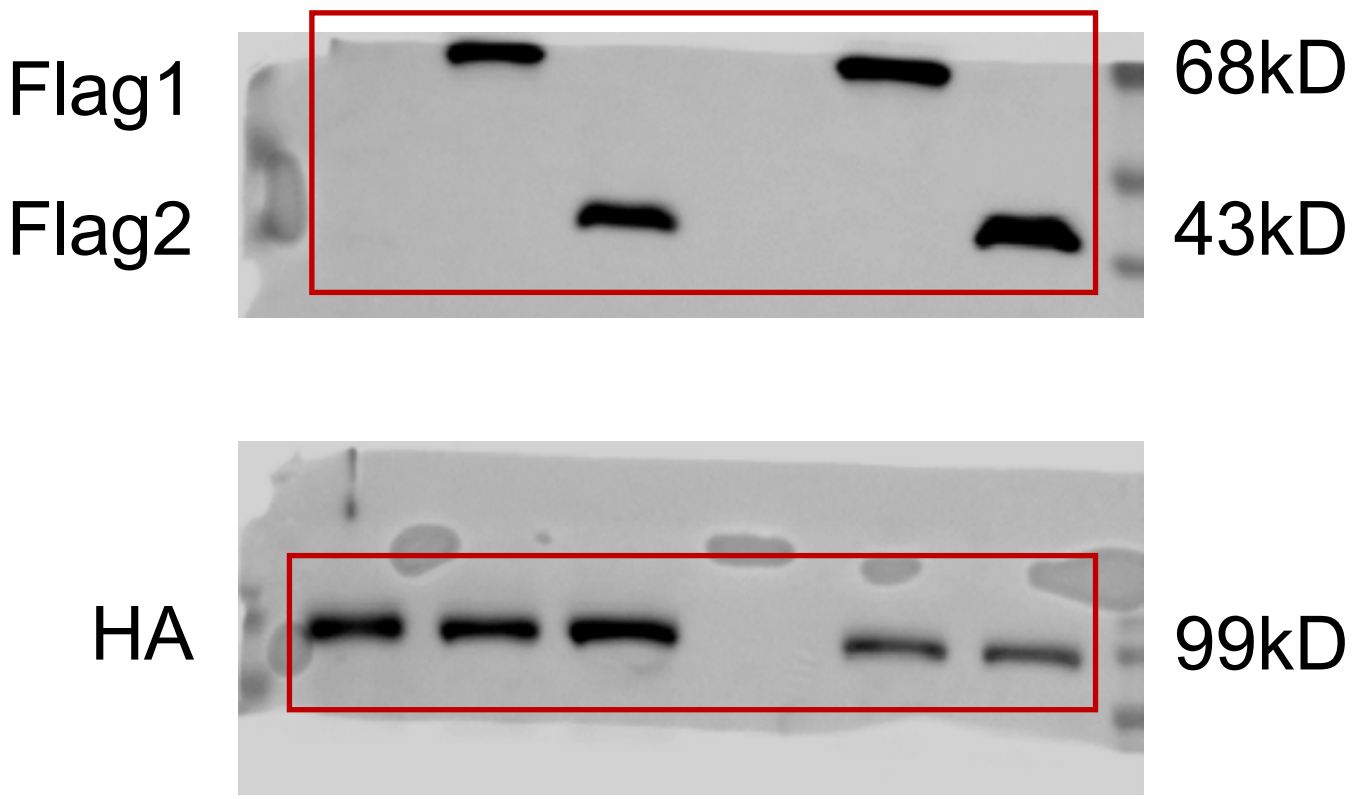

Fig. 6D

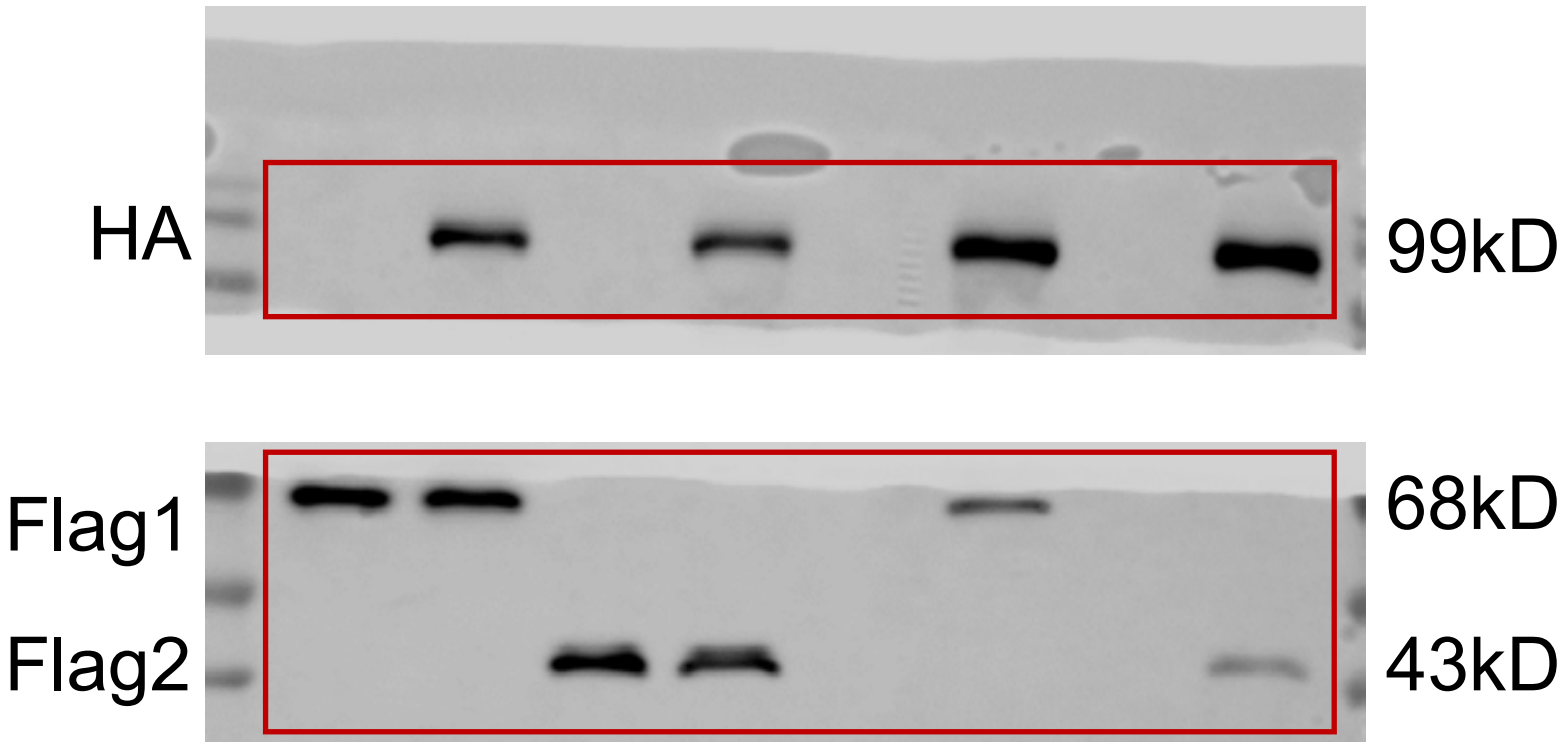

Fig. 6E

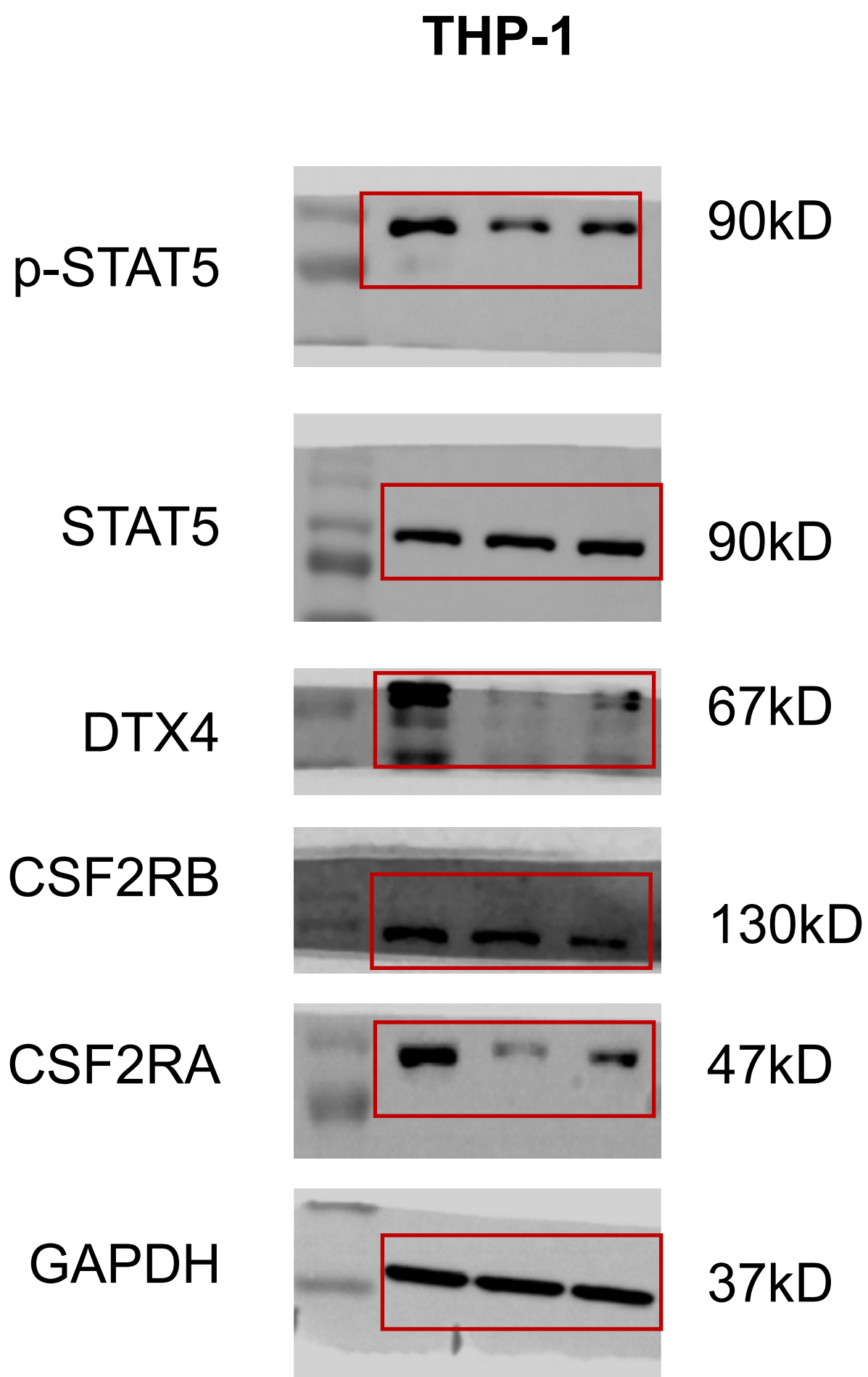
THP-1
p-STAT5
90kD
STAT5
90kD
DTX4
67kD
CSF2RB
130kD
CSF2RA
47kD
GAPDH
37kD

Fig. 6G

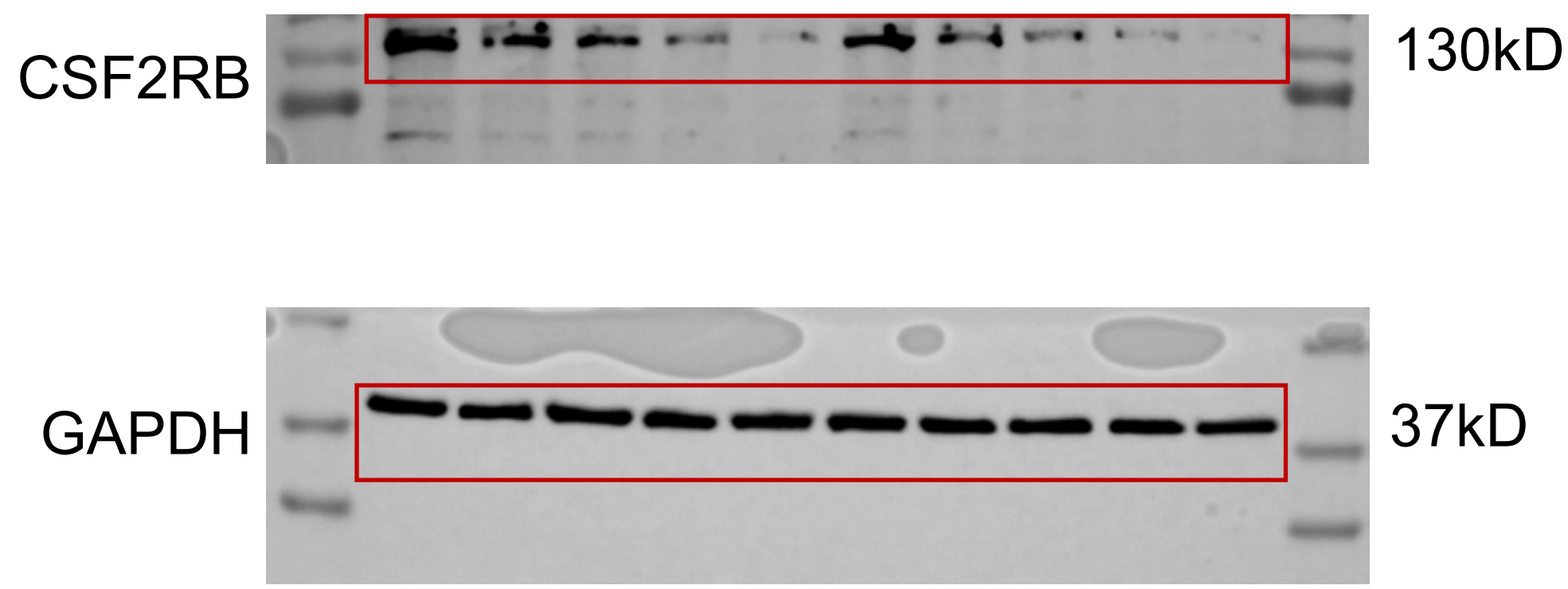
CSF2RB
130kD
GAPDH
37kD

Fig. 6H

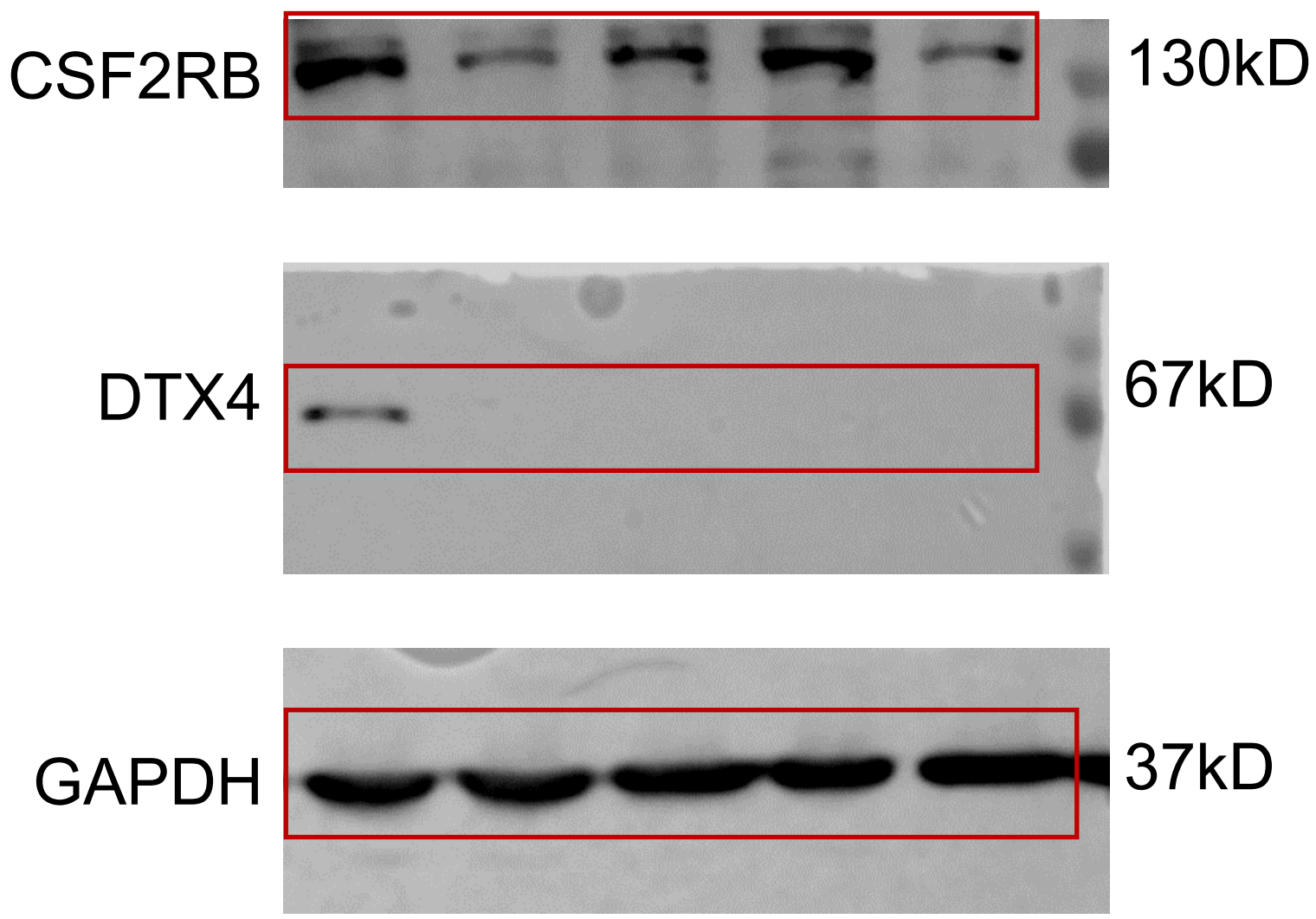
CSF2RB
130kD
DTX4
67kD
GAPDH
37kD

Fig. 6I

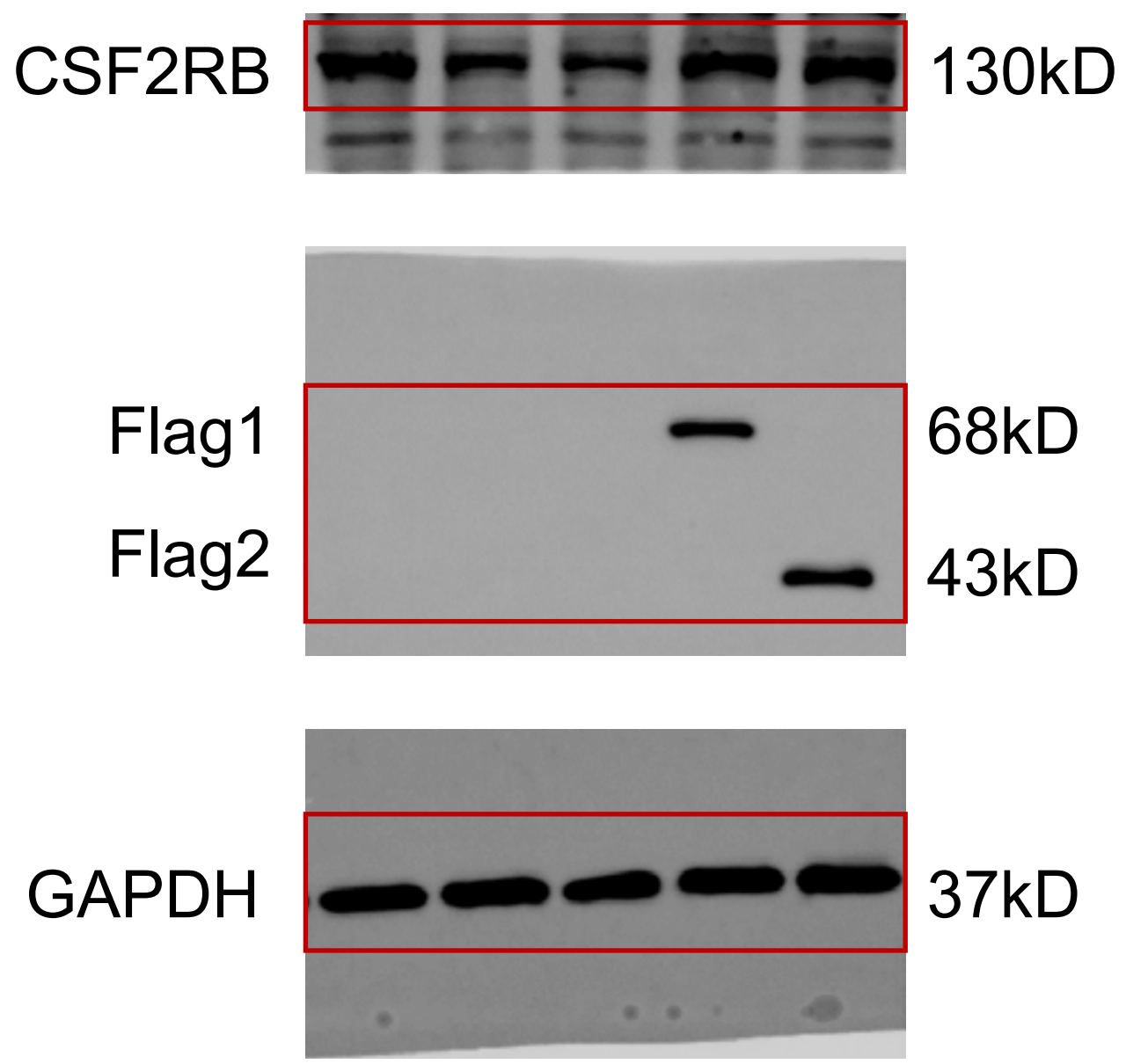
CSF2RB
130kD
Flag1
68kD
Flag2
43kD
GAPDH
37kD

Fig. 6J

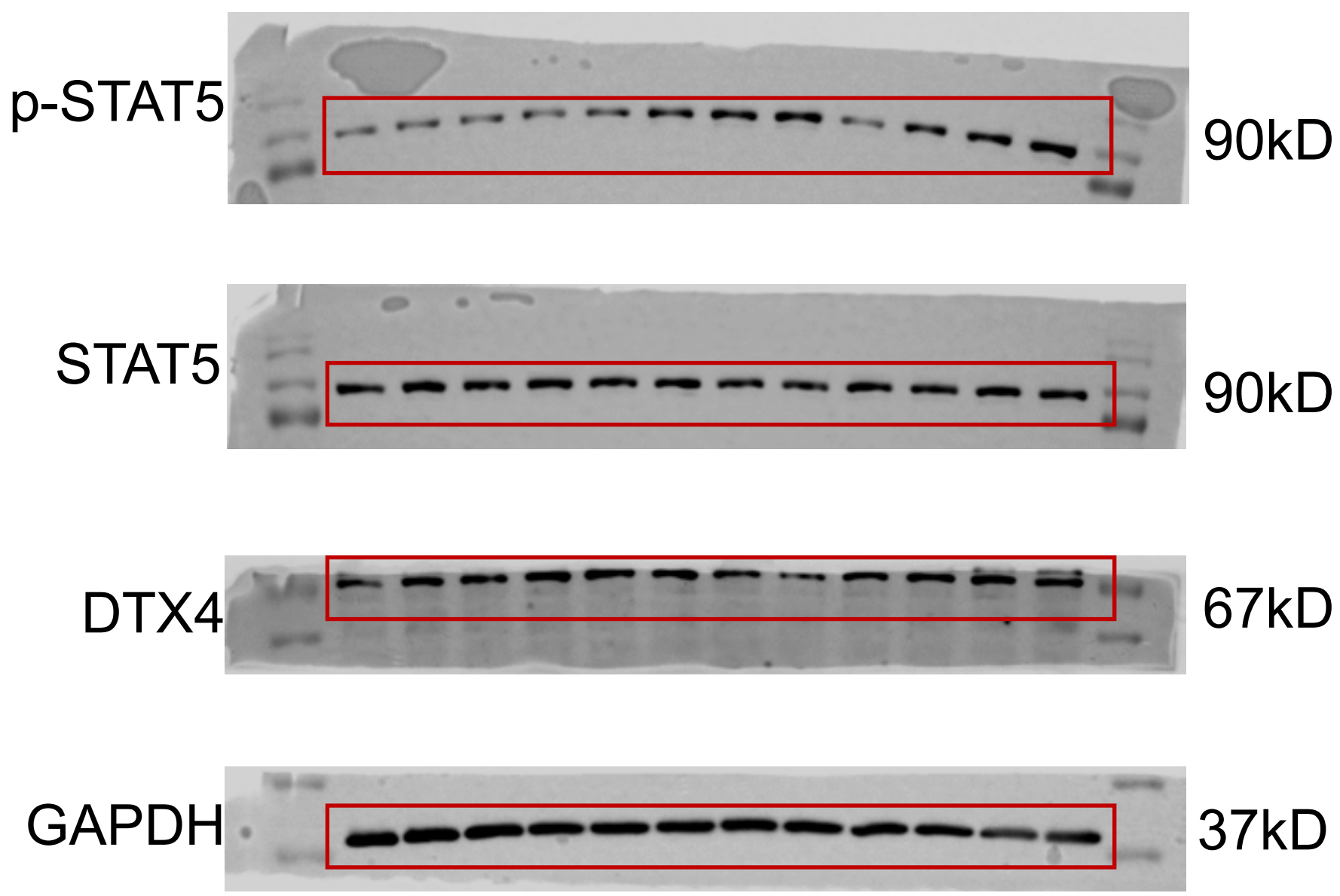
p-STAT5
90kD
STAT5
90kD
DTX4
67kD
GAPDH
37kD

Fig. 6K

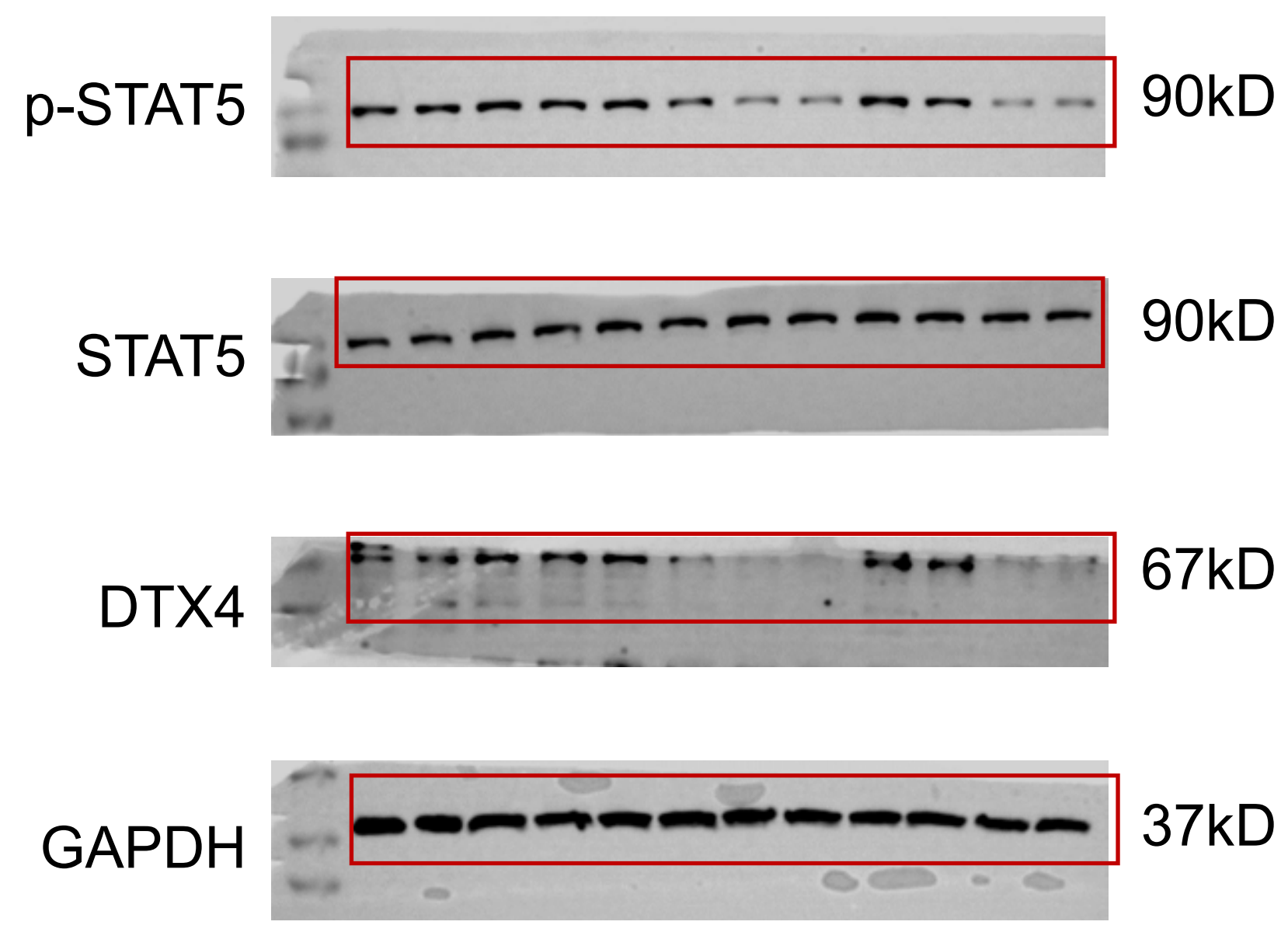
p-STAT5
90kD
STAT5
90kD
DTX4
67kD
GAPDH
37kD

Fig. 7A

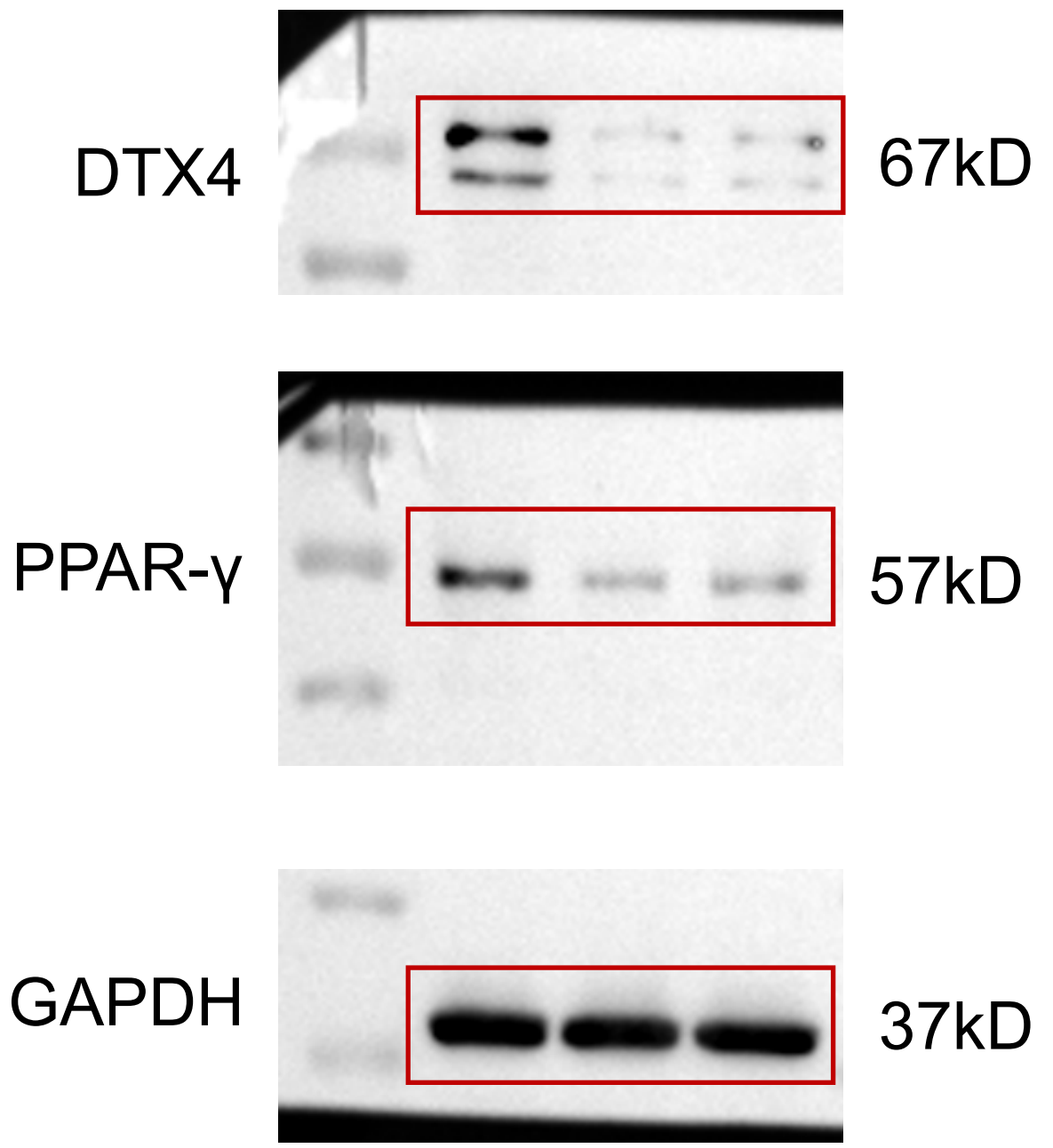

Fig. 7C

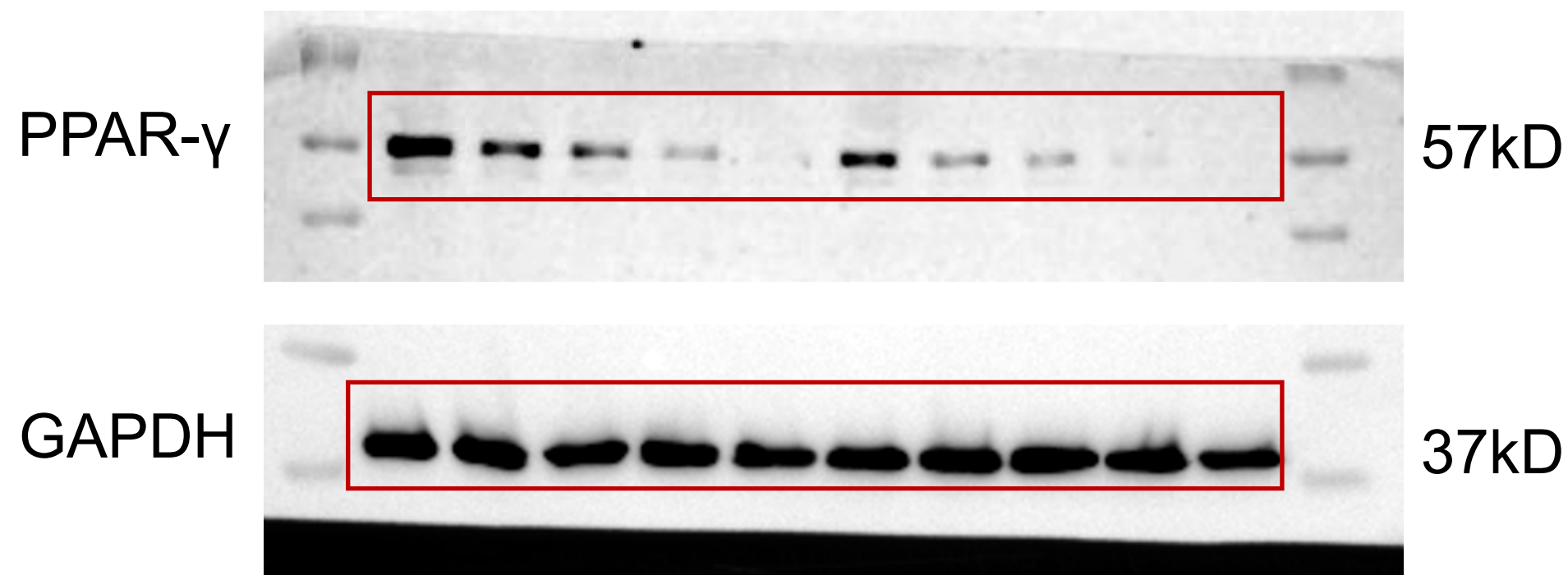

Fig. 7D

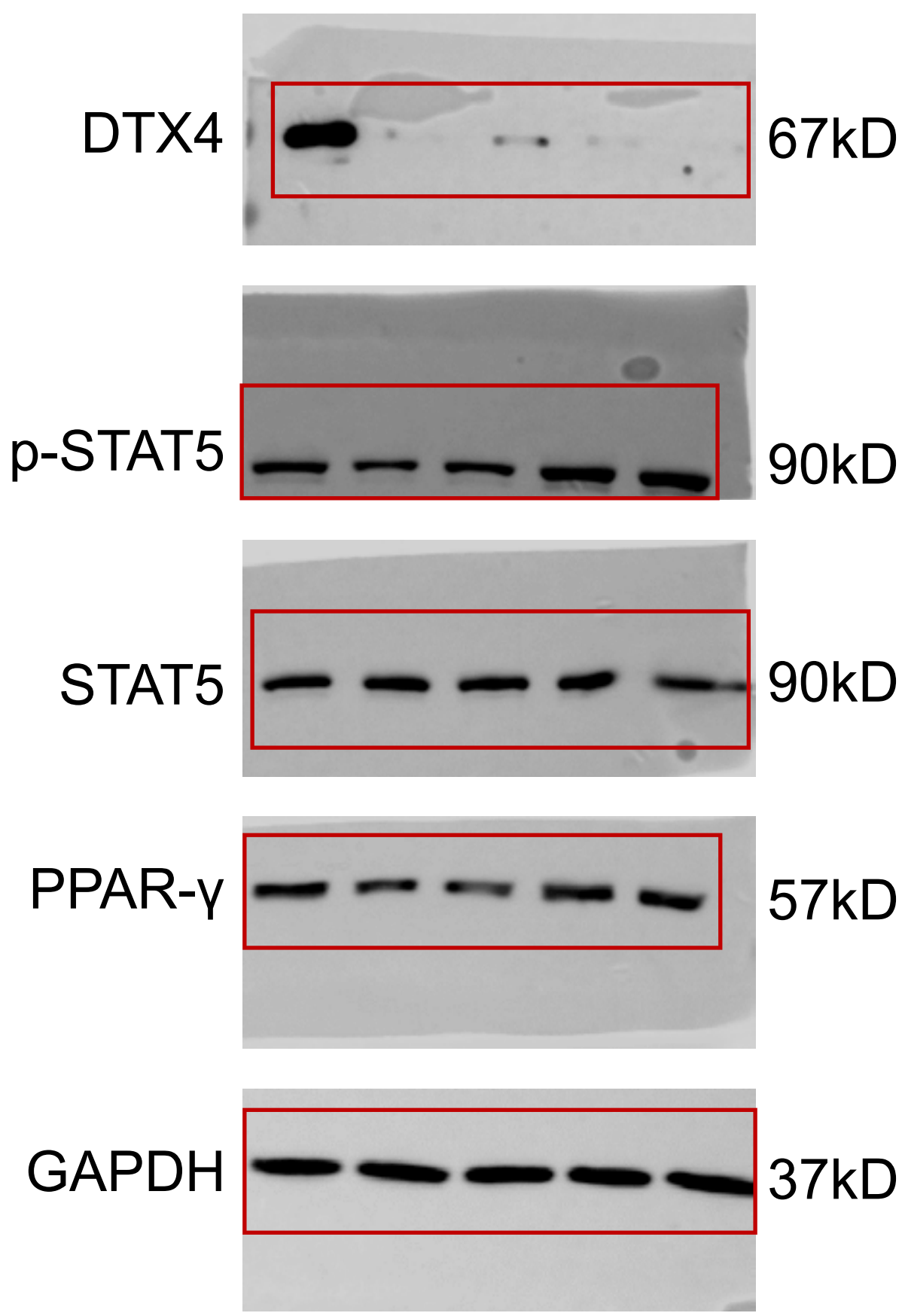
DTX4
67kD
p-STAT5
90kD
STAT5
90kD
PPAR-γ
57kD
GAPDH
37kD

Fig. 7E

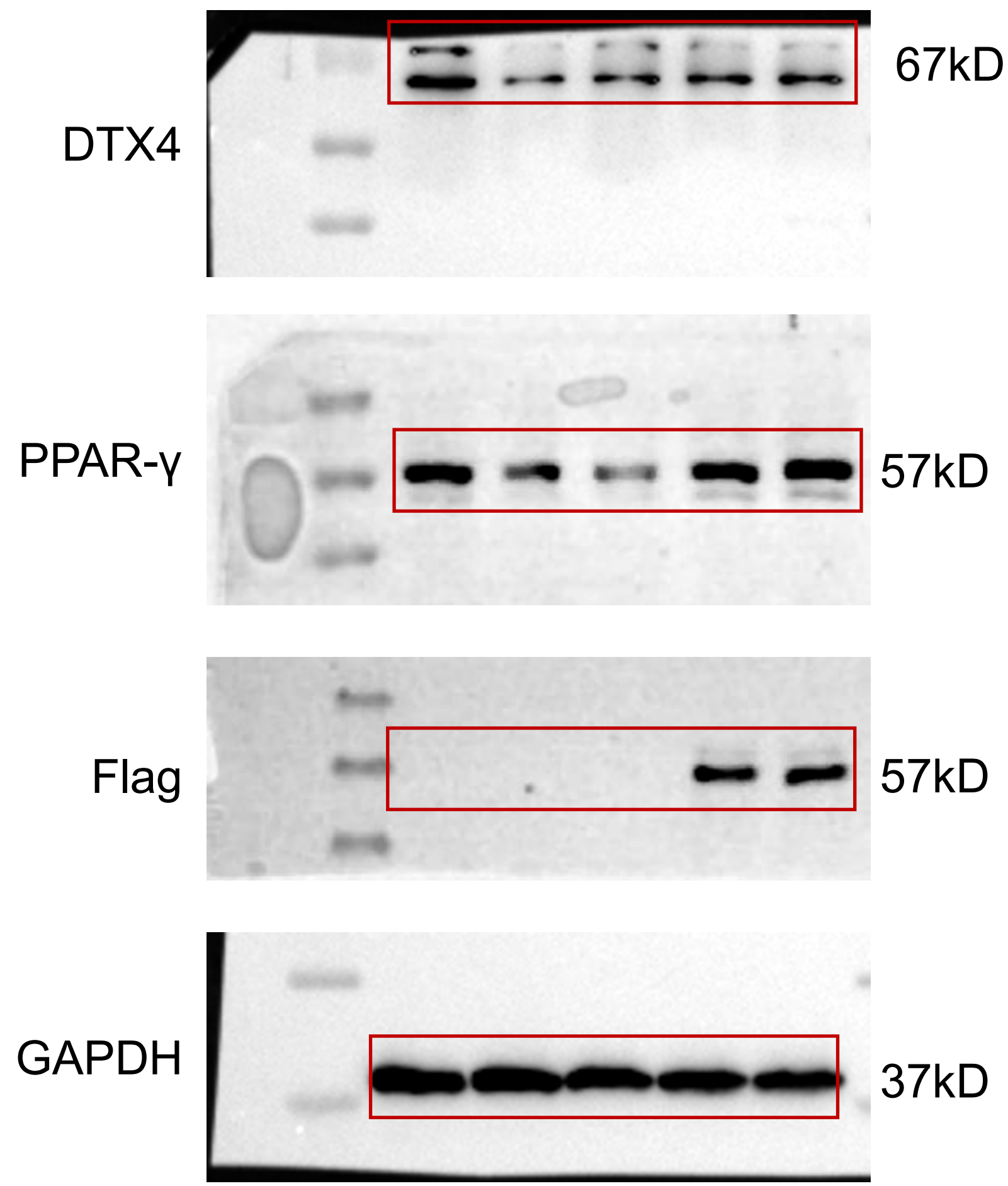
DTX4
67kD
PPAR-γ
57kD
Flag
57kD
GAPDH
37kD

Supplementary Fig. 6A

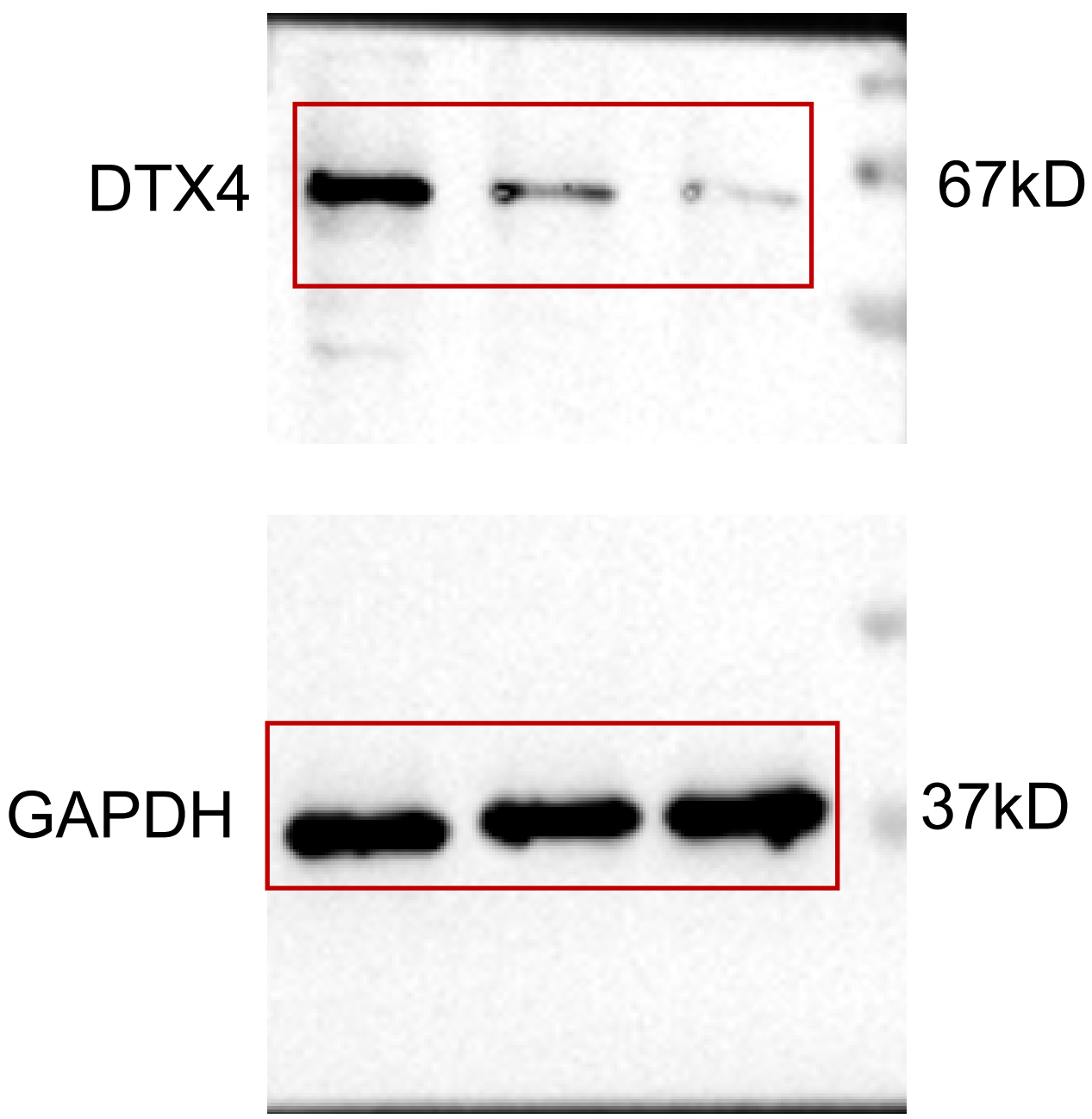

Supplementary Fig. 6B

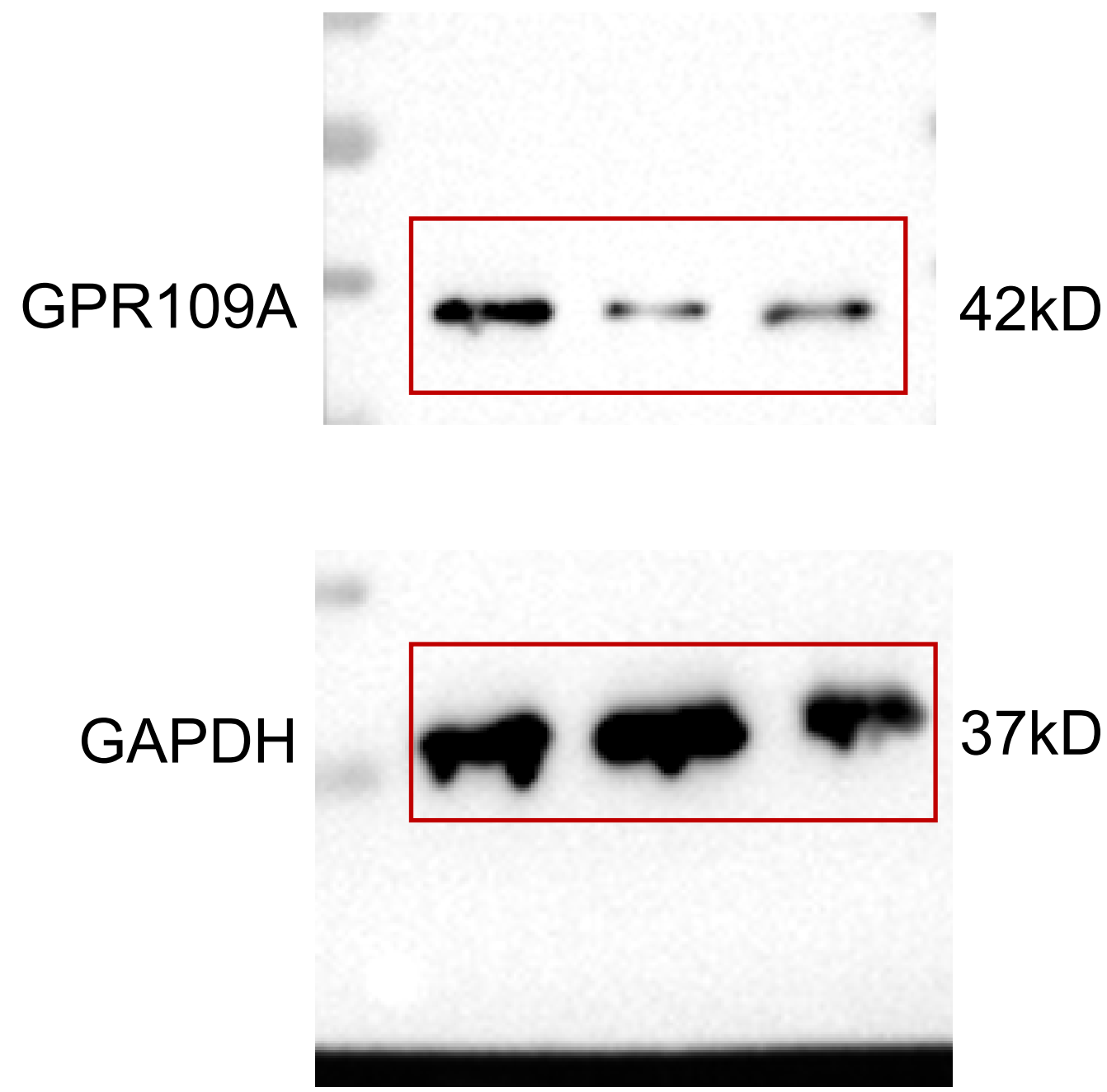

Supplementary Fig. 9A

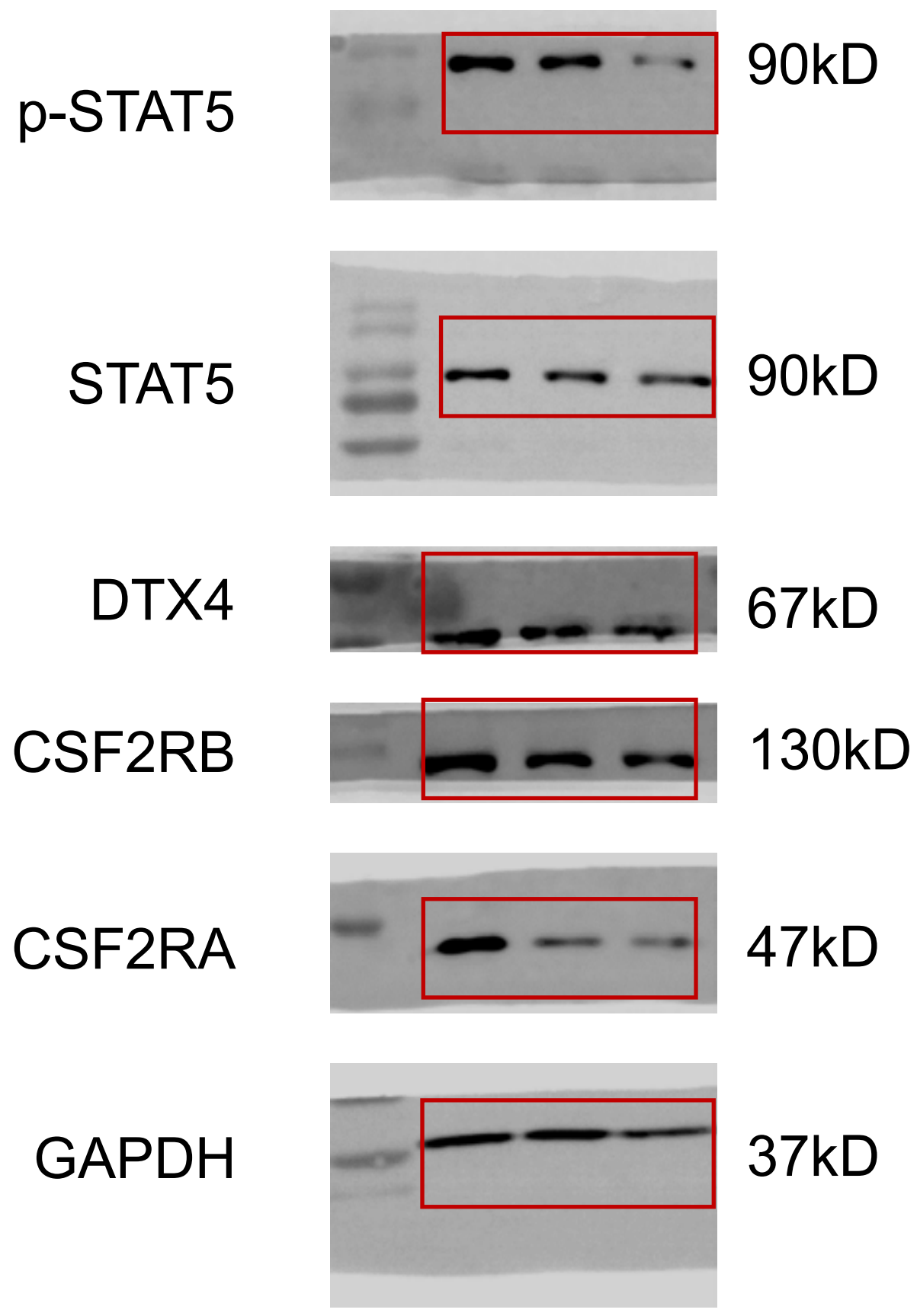

Supplementary Fig. 10A

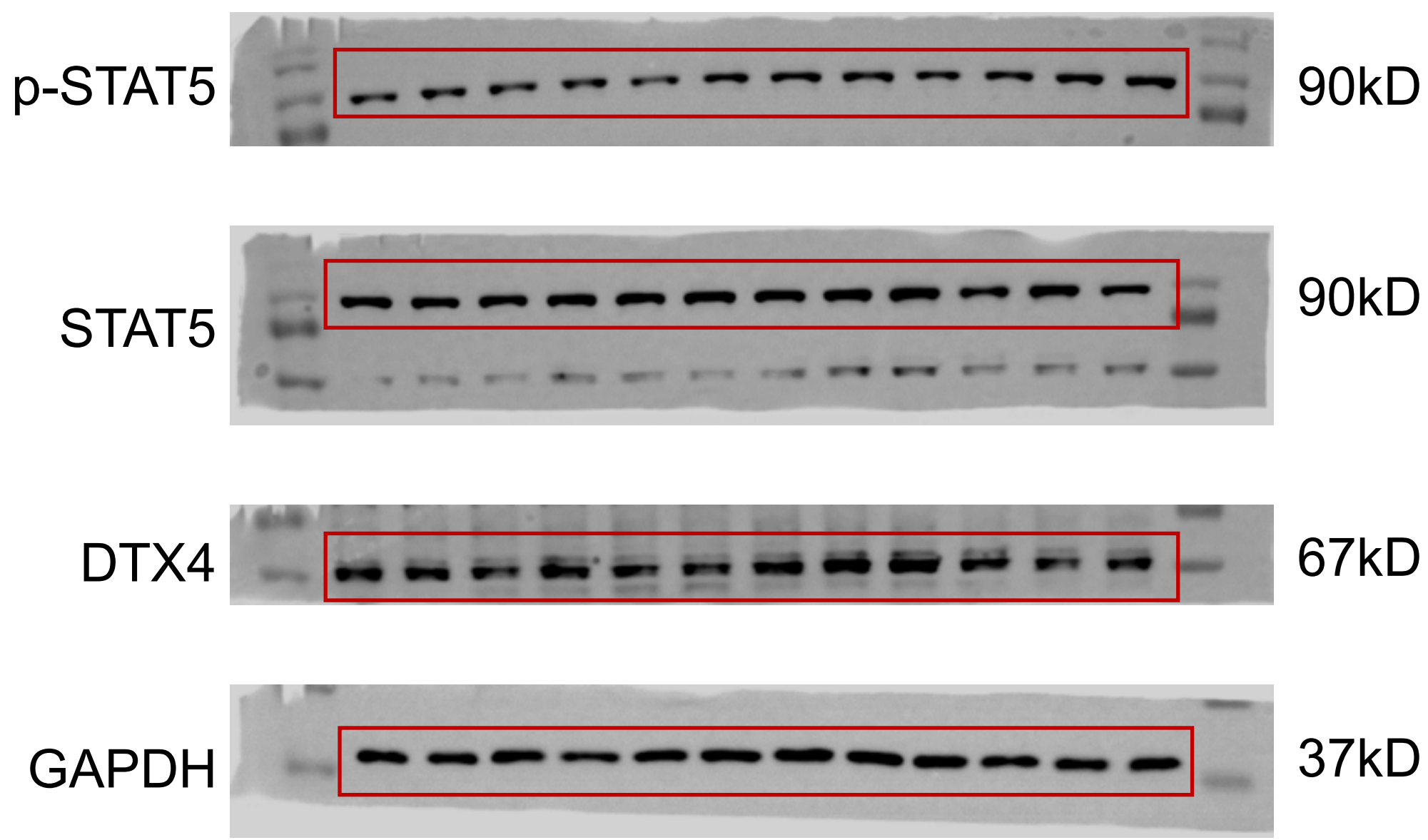

Supplementary Fig. 10B

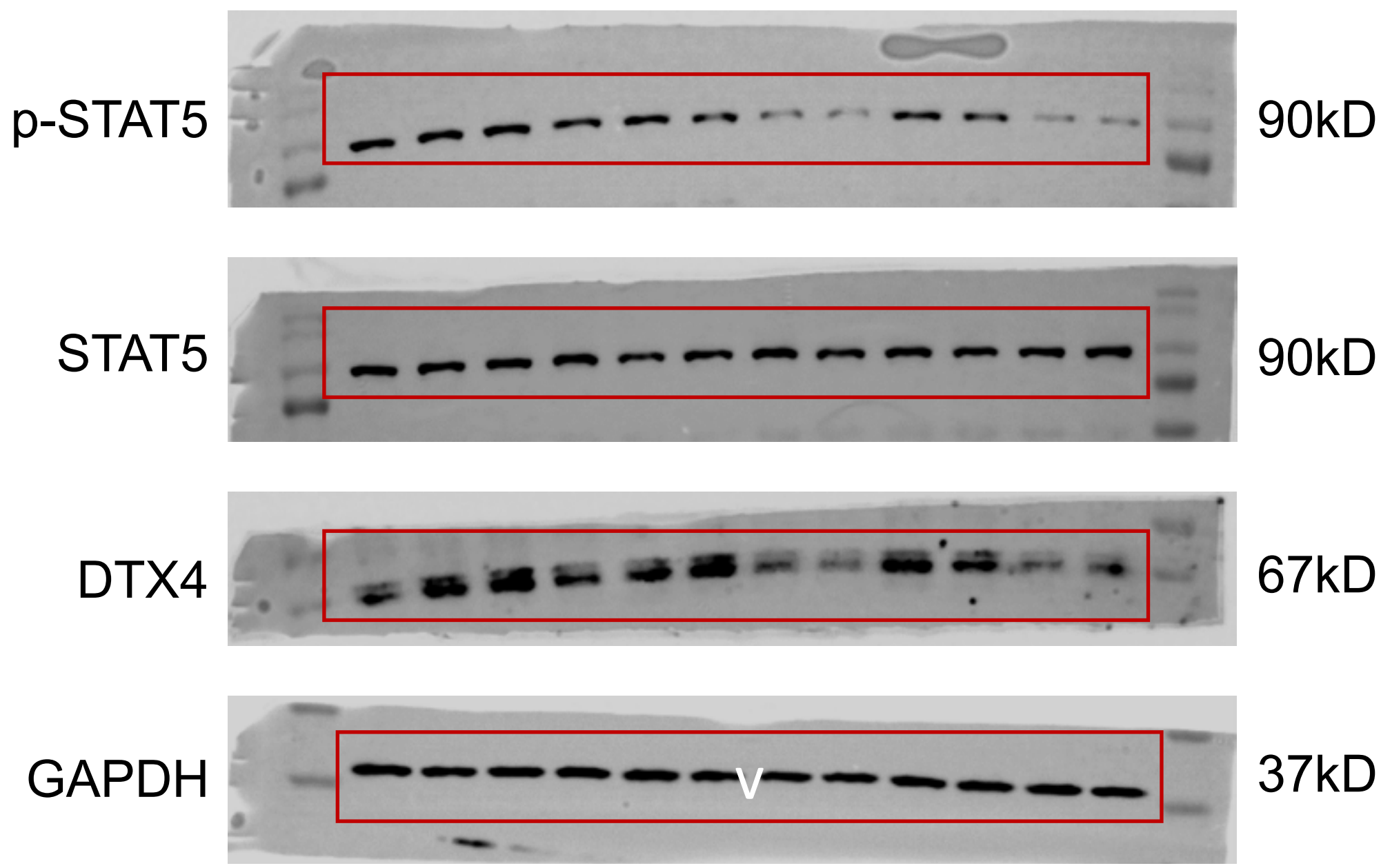

Supplementary Fig. 11A

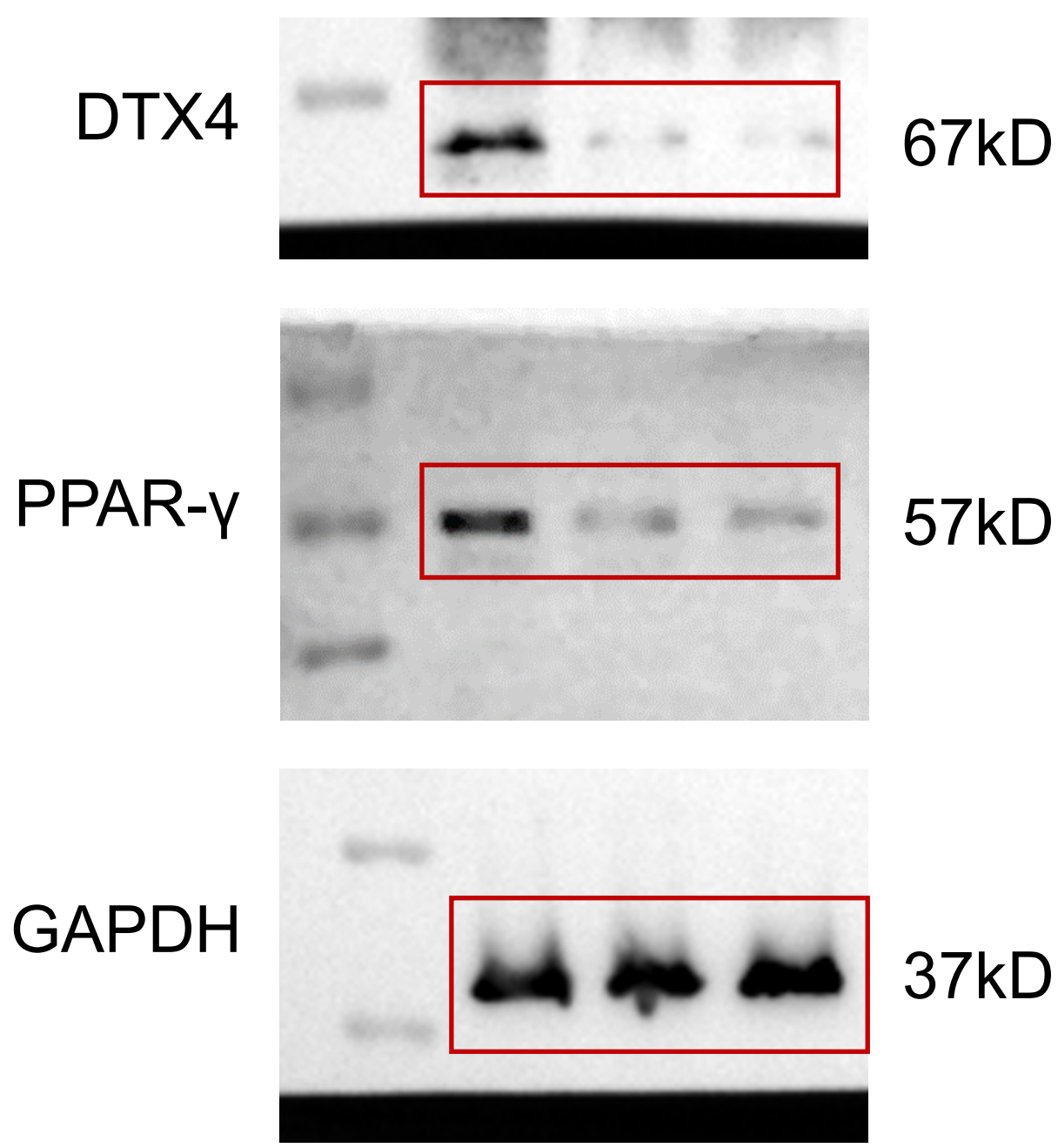

Supplementary Fig. 11D

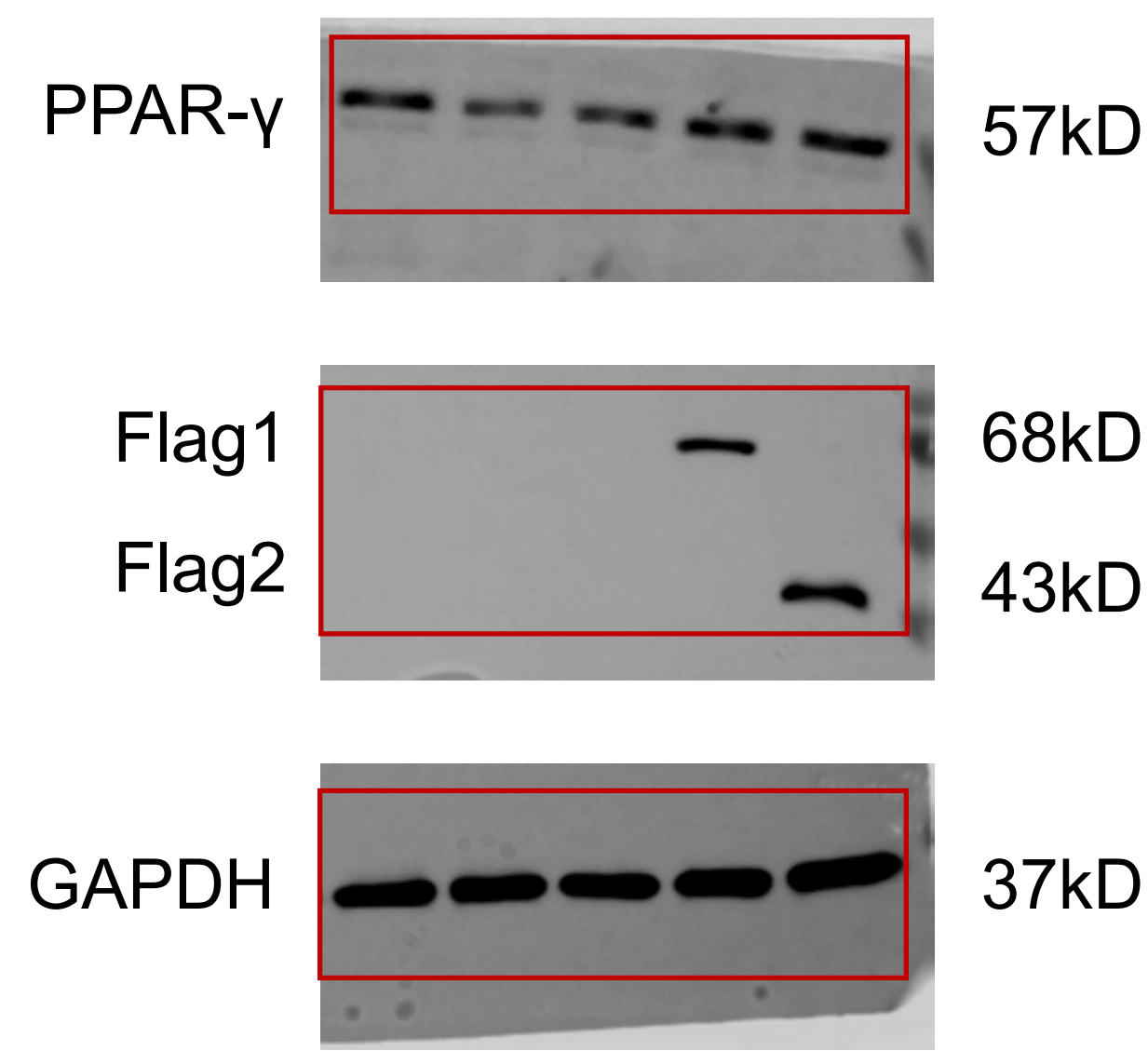

Supplementary Fig. 11H

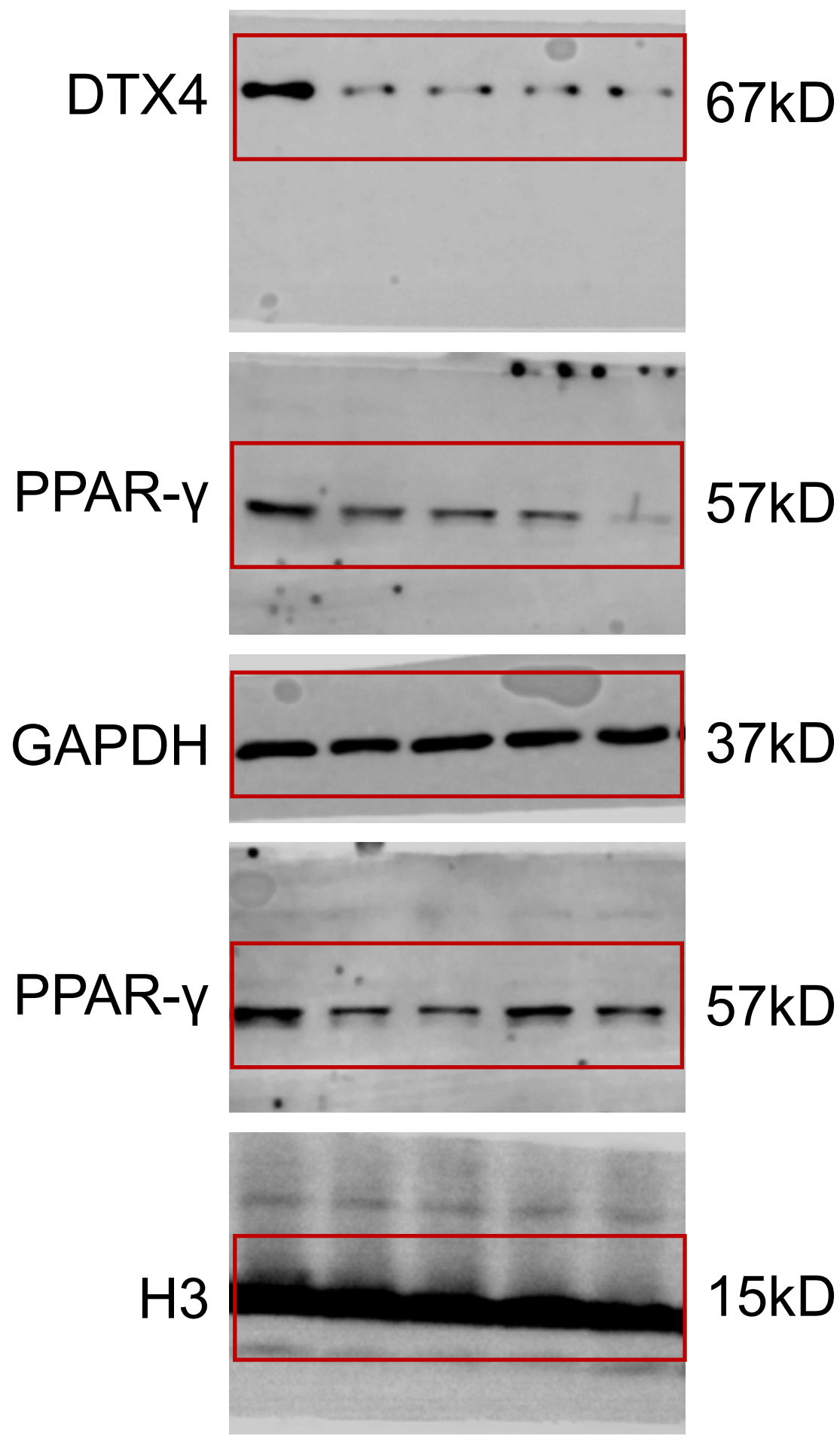

Supplement: Supplementary file 2 [file jciinsight-11-201129-s009.pdf]
